# Rapid chemiexcitation of phenoxy-dioxetane luminophores yields ultrasensitive chemiluminescence assays†
†Electronic supplementary information (ESI) available: Chemiluminescence measurements protocols and data, computational methods and details, synthetic procedures and spectra of key compounds. See DOI: 10.1039/c8sc04280b


**DOI:** 10.1039/c8sc04280b

**Published:** 2018-11-19

**Authors:** Nir Hananya, Jolene P. Reid, Ori Green, Matthew S. Sigman, Doron Shabat

**Affiliations:** a School of Chemistry , Faculty of Exact Sciences , Tel Aviv University , Tel Aviv 69978 , Israel . Email: chdoron@post.tau.ac.il; b Department of Chemistry , University of Utah , 315 South 1400 East , Salt Lake City , Utah 84112 , USA . Email: sigman@chem.utah.edu

## Abstract

The utility of dioxetane-based chemiluminescent probes in biosensing and bioimaging is being increasingly recognized. While phenoxy-dioxetane luminophores with fast chemiexcitation kinetics are highly desired, current luminophores suffer from slow chemiexcitation. Herein we describe a rational, computationally-supported design of phenoxy-dioxetanes with fast chemiexcitation kinetics. These new luminophores were designed to contain a substituent that promotes rapid chemiexcitation, emitting light up to 100-fold faster than currently known dioxetanes. We demonstrate the superiority of the new phenoxy-dioxetanes by preparing three chemiluminescent probes for NAD(P)H, which differ from each other in the rate of the chemiexcitation. Comparison of these probes reveals a correlation between the chemiexcitation rate and the probe sensitivity. We anticipate that these new phenoxy-dioxetanes could serve as an ideal platform for designing chemiluminescence probes with enhanced sensitivity for numerous bioassays.

## Abstract 2

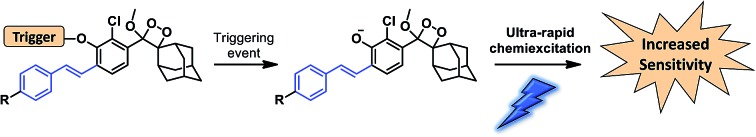
Rational design of phenoxy-dioxetane luminophores with rapid chemiexcitation is described; these next generation luminophores yielded chemiluminescent probes with considerably increased sensitivity.

## Introduction

**Scheme 1:**
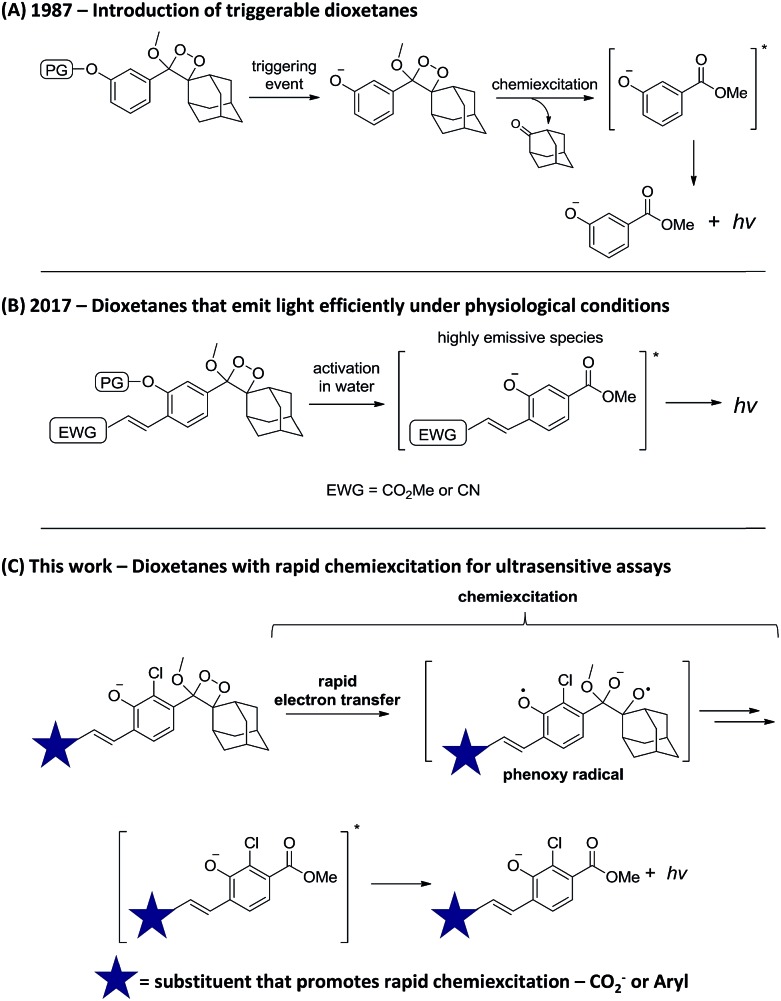
Progress of the chemistry of chemiluminescent dioxetanes.

Chemiluminescence (CL) is increasingly recognized as a powerful tool for sensing and imaging.^1^,^2^ Its main advantage over fluorescence lies in the fact that irradiation by an external light source is not required, thus the background signal is extremely low and the obtained sensitivity is considerably enhanced.^3^,^4^ Two major breakthroughs in the chemistry of chemiluminescent compounds have established this strategy as a general and efficient tool for bioimaging. The first, which took place >30 years ago, was the introduction of triggerable dioxetanes by Schaap.^5^ With these compounds, light emission is a consequence of phenolate formation following phenol deprotection. Thus, by selecting the appropriate phenol-protecting group, one can directly determine the event that triggers light emission (Scheme 1A). This modularity of the dioxetanes enabled the preparation of numerous dioxetane-based chemiluminescent probes;^6^–^8^ the most recognized of which is the alkaline phosphatase substrate CDP-Star®.^9^,^10^ The second breakthrough was reported last year by our group, when a subtle change in the phenoxy-dioxetanes structure enabled the development of remarkably efficient emitters under physiological conditions.^11^ Specifically, it was demonstrated that by introducing an electron-withdrawing acrylic group at the *ortho* position of the phenol donor, a 3000-fold increase in CL quantum yield (*Φ*_CL_) could be obtained (Scheme 1B). This new class of phenoxy-dioxetane-based luminophores has been applied by us and others to construct CL probes for detection and imaging of various enzymes and analytes.^12^–^18^ Herein, we present next generation phenoxy-dioxetanes, which through a rational, computationally-supported design were equipped with substituents that promote an increased rate of chemiexcitation (Scheme 1C).

Rapid chemiexcitation is highly desirable, as it is anticipated to improve the sensitivity of chemiluminescent analytical bioassays. As photons are being released within a short period of time, signal to background ratio (S/B) is higher and sensitivity is better.^19^ Thus, the new luminophores reported here were specifically designed to undergo fast light-emitting chemiexcitation following phenolate formation. Furthermore, we demonstrate how this improvement of light-emission kinetics yields CL assays with superior sensitivity.

## Results

### Rational design and synthesis of phenoxy-dioxetane luminophores with rapid chemiexcitation

**Fig. 1:**
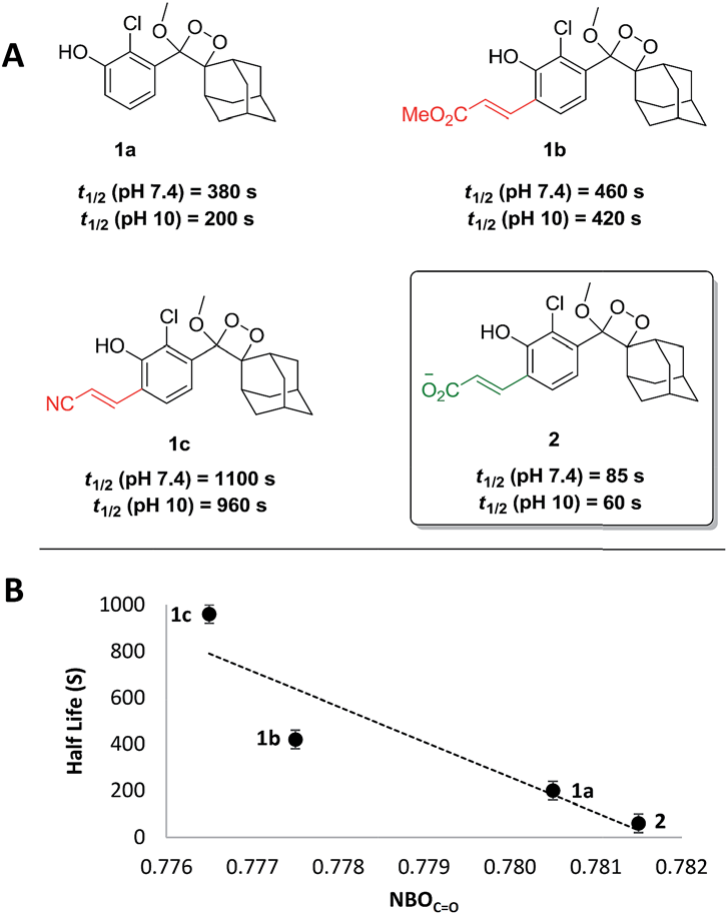
(A) Structure and half-life of the previously reported luminophores **1a–c** and the new luminophore **2** with accelerated chemiexcitation. (B) Half-life of luminophores at pH 10 as a function of substituent's NBO_C=O_. *R*^2^ = 0.83.

As a starting point, the chemiexcitation kinetics of recently reported luminophores **1b** and **1c**,^11^ which are sufficiently bright under physiological conditions, were evaluated.§
§As expected, the decomposition profile of all luminophores described in this paper followed first-order kinetics; thus, the rate constant, as well as *t*_1/2_, were derived from the linear graph of ln(RLU) *vs.* time. See ESI.†
 Noticeably, the observed kinetics are relatively slow, in comparison to the simple unsubstituted luminophore **1a** (Fig. 1A). We therefore sought to rationally modify the electronic structure of the luminophore in order to achieve faster kinetics, based on mechanistic analysis of chemiexcitation. Although the details of the chemiexcitation process remain controversial,^20^–^23^ there is a consensus that its first step is an electron transfer from the phenolate donor to the dioxetane moiety, generating a phenoxy-radical species (see Scheme 1C; see ref. 23 for a comprehensive review). Therefore, the rate of the chemiexcitation is controlled at least by two effects. First, increased electron density of the phenolate should promote faster chemiexcitation, while the converse, presence of an electron-withdrawing substituent, retards this process. This is consistent with the slower chemiexcitation of the substituted phenoxy-dioxetanes **1b** and **1c**, compared to the unsubstituted **1a**. Second, as the radical-stabilizing nature of the phenoxy moiety is increased, the chemiexcitation rate is accelerated.

As such, our initial efforts to enhance chemiexcitation rate were focused on increasing the phenolate electron density. Therefore, phenol-dioxetane **2** was prepared with an acrylic acid *ortho* substituent. Under physiological conditions the acrylic acid exists in the form of acrylate anion, which is the least electron-withdrawing substituent among all other acrylate derivatives. The CL kinetic profile of **2** under physiological conditions was measured, and, as expected, was found to be 5.5-fold faster than **1b** and 13-fold faster than **1c** (Fig. 1A, *t*_1/2_ = 85 s at pH 7.4). Since the active species in the chemiexcitation process is the phenolate anion, chemiexcitation kinetics were further measured at pH 10 (Fig. 1A). In this manner, the chemiexcitation rate of the phenolate species could be evaluated, eliminating the effect of phenol p*K*_a_ differences. Changing the pH from 7.4 to 10 did not significantly impact the general trend among the half-lives of luminophores, thus confirming that the differences in the chemiexcitation rate are intrinsic and do not stem from different p*K*_a_ values.

To further understand the effect of the substituent's electron-withdrawing character on the rate of the chemiexcitation, a study of the phenolate chemiexcitation rate as a function of electronic effects of luminophores was investigated. Hammett values have typically been employed to afford a quantitative measure of the electron withdrawing/donating ability of aromatic substituents.^24^,^25^ Previous efforts have shown that NBO charges and IR carbonyl stretching frequencies can be used as an alternative to empirically derived Hammett values, *σ*_p_.^26^ Considering the substituents under evaluation, we employed the NBO_C=O,_ the charge on the benzoic acid moiety (see ESI for details†).¶
¶We also considered HOMO energies as a descriptor. However, no correlation could be established. As depicted in Fig. 1B, NBO_C=O_ values correlate well with chemiexcitation kinetics; as the substituent is more electron withdrawing, the chemiexcitation rate of the luminophore becomes slower.

**Fig. 2:**
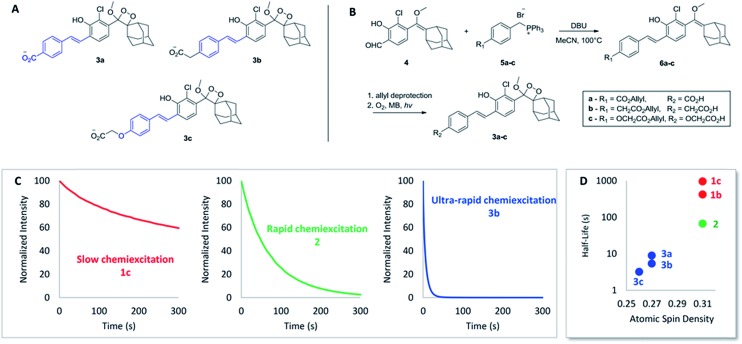
(A) Structure of styryl-substituted luminophores **3a–c**. (B) General synthetic pathway of **3a–c**. (C) CL kinetic profiles of **1c**, **2** and **3b**. 20 μL of luminophore solution in DMSO [10 μM] were injected to 180 μL of aqueous buffer and CL was measured immediately. See ESI for full data.† (D) Relationship between radical-stabilizing character of the luminophores and their chemiexcitation kinetics.

Encouraged by these results, we sought to design luminophores with even faster chemiexcitation kinetics. Replacing the acryl-substituents on the phenoxy-dioxetanes with styryl-substituents (Fig. 2A) was hypothesized to be a handle to improve the performance for the following two reasons. First, the phenyl ring can serve as a modular platform for modifying the electron withdrawing/donating nature of the conjugated substituent. Second, extended conjugation of the styryl substituent is anticipated to increase the radical stabilizing nature of the phenoxy moiety. As noted above, a more stabilized phenoxy radical should lead to faster chemiexcitation (see Scheme 1C). To test this hypothesis, luminophores **3a–c** were synthesized, based on a Wittig reaction between aldehyde **4** and substituted benzyltriphenyl-phosphonium bromides **5a–c** (Fig. 2B, see ESI for synthetic details†). A carboxylic acid was incorporated in each luminophore, to increase aqueous solubility. The CL kinetic profiles of luminophores **3a–c** were measured and compared to those of luminophores **1a–c** and **2**. Remarkably, chemiexcitation of the styryl derivatives occurred extremely rapidly. For example, **3b** (as a median representative of the styryl derivatives) has a half-life shorter by more than 200-fold than that of **1c**, and by more than 15-fold than that of **2** (Fig. 2C). It should be noted that this outstanding performance of luminophores **3a–c** appears not only at pH 10 but also under physiologically relevant conditions (pH 7.4; see Table 1 below. For full kinetic data see ESI†).

**Table 1:** Experimental and computational properties of luminophores

	λ_max_ (nm)	*t*_1/2,7.4_ (s)	*t*_1/2,10_ (s)	*Φ*_CL_ (%)	NBO_C=O_	O–H BDE^*a*^ (kcal mol^–1^)	Spin Density^*a*^
**1a**	470	380	200	0.003	0.780	88.1	0.34
**1b**	550	460	420	2.3	0.777	86.7	0.31
**1c**	525	1100	960	13.8	0.776	86.9	0.31
**2**	510	85	60	9.8	0.781	85.0	0.31
**3a**	535	8.4	7.0	4.2	0.780	84.9	0.27
**3b**	500	5.1	4.2	1.4	0.779	83.8	0.27
**3c**	490	3.2	2.7	0.6	0.780	82.6	0.26

^*a*^Simplified compounds were used for calculations. See ESI for details.

To further explore the relationship between the phenoxy-radical stability and the chemiexcitation kinetics, radical stabilizing properties of the acryl- and the styryl-substituted dioxetanes were examined using DFT calculations. We used a slightly simplified structural surrogate in our calculations, focusing on the conserved phenol conjugated π-system wherein the methoxy-dioxetane-adamantane portion was replaced with a hydrogen (see ESI†). The O–H bond dissociation energies (BDEs) of these compounds were calculated at the SMD-water-UM06-2X/def2-TZVP level of theory (see ESI for details†). It was determined that the introduction of a phenyl group has a dramatic radical stabilizing effect, as the styryl-substituted phenoxy-radicals (BDE = 82.6–84.9 kcal mol^–1^) were found to be more stable than the acryl-substituted series (BDE = 85.0–86.9 kcal mol^–1^). To further clarify and contextualize these energetic results, natural bond orbitals were used to evaluate the atomic spin distribution in the molecule. As expected, the atomic spin density at the radical center for the acrylate series (0.31) is significantly higher than those derived from the styryl-substituted compounds (0.26–0.27), consistent with the BDE results. Altogether the calculations support our assumption that the styryl substituents better stabilize the phenoxy radical, compared to the acryl substituents. An evident relationship is revealed between the chemiexcitation kinetics and the radical stability, supporting our hypothesis that the styryl substituents promote rapid chemiexcitation by stabilizing the radical to a greater extent, as opposed to the acryl derivatives (Fig. 2D).

Table 1 summarizes the CL properties and the computational parameters of acryl- and styryl-substituted luminophores. Overall, the combination of experiment and calculation suggest that radical stability can explain the differences between the kinetics of acryl- and styryl-substituted luminophores. However, it is also clear that radical stability is not the sole structural effect that determines chemiexcitation kinetics. For example, BDEs and atomic spin density calculations clearly show that the unsubstituted derivative **1a** stabilizes the radical the least (BDE = 88.1 kcal mol^–1^, spin density = 0.34); however, its chemiexcitation rate is faster than those of **1b** and **1c** with greater radical stabilizing properties (BDE = 86.7–86.9 kcal mol^–1^, spin density = 0.31). As proposed above, polar effects (electron donation/withdrawal) also have an impact on the kinetics, and this can explain the trend well when radical stabilizing groups are not present (see Fig. 1). Both effects are difficult to delineate, since BDEs and NBO_C=O_ values are sensitive to similar structural components in many cases. In sum, the performed calculations clearly support our rational design of dioxetanes with rapid chemiexcitation.

Our strategy successfully resulted in phenoxy-dioxetanes with improved chemiexcitation kinetics. However, for these luminophores to be effective in the construction of chemiluminescent probes, it was essential to verify that they maintain sufficiently high *Φ*_CL_. *Φ*_CL_ of the luminophores was examined by measuring their total light emission. All new luminophores exhibit *Φ*_CL_ at least 200-fold higher than that of Schaap's dioxetane **1a** (Table 1); luminophore **2** has *Φ*_CL_ of approximately 10%, and luminophores **3a–c** are somewhat less bright with their *Φ*_CL_ ranges from 0.6% to 4.2%. However, as will be discussed further below, the absolute *Φ*_CL_ is not a crucial parameter when using the dioxetanes as chemiluminescent probes in test-tube bioassays (as long as the luminophore stands at a minimum threshold of brightness). This is because the key value in these assays is the signal to background ratio and not the absolute amount of light emitted (see Discussion).

### Ultrasensitive chemiluminescent probes for NAD(P)H

**Fig. 3:**
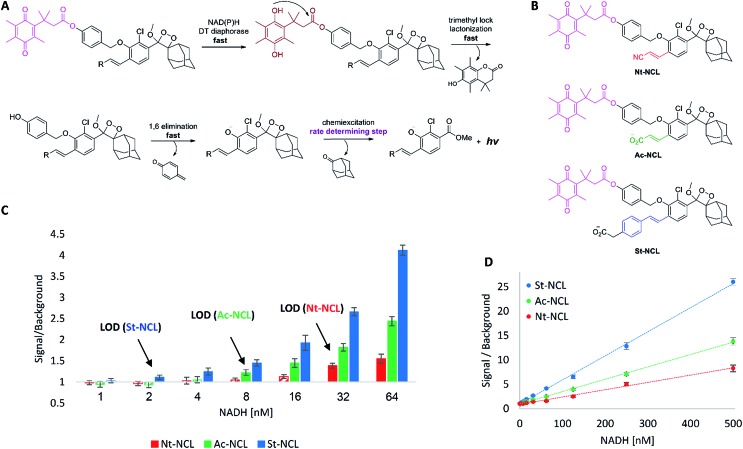
(A) General activation pathway of NAD(P)H chemiluminescent probe. (B) Structures of the NAD(P)H probes. (C) NADH detection by the probes, focus on the lowest concentrations. Probe [50 μM] was incubated with varying concentrations of NADH in the presence of NQO1 enzyme [20 μg mL^–1^], and CL signal was measured. Signal/background value was chosen as the highest value for each assay, which was less than 60 s following NADH addition. LOD was defined as blank + 3SD. Measurements were performed at PBS [100 mM], pH 7.4, 5% DMSO, 25 °C. (D) Signal/background expanded to include higher NADH concentrations.

Next, we sought to demonstrate the advantage of the fast chemiexcitation observed for the new acrylate-anion-substituted dioxetane and the styryl-substituted dioxetane by the generation of CL probes for NADH and NADPH. These dinucleotides are important electron carriers found in all living cells. They participate in multiple redox reactions such as NAD(P)/NAD(P)H-dependent energy metabolic pathways,^27^,^28^ or maintenance of intracellular redox status.^29^ Therefore, the ability to measure NAD(P)H levels quantitatively provides an useful tool for investigating metabolism and for monitoring oxidative stress response. Several methods have been developed for NAD(P)H detection.^30^,^31^ One promising approach relies on an NAD(P)H-dependent quinone reduction, which leads to the release of a reporter moiety in the form of free aniline or phenol (Fig. 3A).^32^ DT diaphorase utilizes NAD(P)H present in the analyzed sample to reduce a quinone substrate. The resulting hydroquinone has a unique “trimethyl-lock” structure, which induces fast lactonization.^33^ Subsequent 1,6-elimination yields free phenol-dioxetane that decompose through chemiexcitation to emit light. At high enzyme concentration, the first three steps should take place within seconds, making the chemiexcitation rate determining.

Three chemiluminescent probes were synthesized (see ESI for synthetic procedures†): acrylonitrile-substituted **Nt-NCL**, acrylate-substituted **Ac-NCL**, and styryl-substituted **St-NCL** (Fig. 3B). The probes were incubated along with DT diaphorase (NQO1) and tested for their ability to detect different NADH concentrations (Fig. 3C and D). **Nt-NCL**, with slow chemiexcitation kinetics following activation by NADH, demonstrated modest sensitivity (LOD = 32 nM). However, **Ac-NCL**, which underwent rapid chemiexcitation following activation, was 4-fold more sensitive with an LOD of 8 nM. Remarkably, **St-NCL**, which exhibited ultra-rapid chemiexcitation, was found as the most sensitive NAD(P)H probe. It was 16-fold more sensitive than **Nt-NCL** and 4-fold more sensitive than **Ac-NCL** (LOD = 2 nM). These results highlight the superior performance of the new rapidly light-emitting dioxetanes.

## Discussion

Chemiluminescence is becoming more and more popular as an imaging tool in bioassays. Compared to fluorescence, it has one major advantage and one major drawback. On the one hand, the background signal in chemiluminescence is significantly lower; since an external light source is not required. On the other hand, chemiluminescent probes generate considerably lower number of photons than fluorescent probes; since fluorescent molecules can be repeatedly excited by the irradiation of external light to emit numerous photons. Consequently, the superiority of chemiluminescence is expressed predominantly in applications in which the absolute amount of light is not decisive.

It is therefore not surprising that chemiluminescence has primarily been used for bioanalytical assays performed in a test-tube setting. For such assays, in which the probe concentration is generally not limited, the absolute amount of light emitted by each molecule does not have substantial implication on the signal/background. As long as the signal is intense enough to be readily quantified by the detector, the effect of *Φ*_CL_ of the chemiluminescent probe on the signal/background is negligible. The reason for this lies in the fact that for dioxetane-based chemiluminescent assays, the background originates almost exclusively from the slow spontaneous hydrolysis of the triggering moiety and not from any external interference. Therefore, luminophore with higher *Φ*_CL_ also generates a higher background.

In light of the above, our main aim in this work was to develop luminophores with faster chemiexcitation rate (as we realized that rapid chemiexcitation will improve the signal/background for such assays). We anticipated that improving the chemiexcitation kinetics will yield probes with higher sensitivity, even if the rapid chemiexcitation comes to a certain extent at the expense of high *Φ*_CL_. Indeed, we were able to develop new phenoxy-dioxetane luminophores with up to 100-fold more rapid chemiexcitation kinetics. Despite the fact that the styryl-substituted dioxetanes are in general less bright than the acryl-substituted derivatives, the styryl-based probe **St-NCL** yields significantly more sensitive assay for NADH than **Nt-NCL** and **Ac-NCL**.

The incorporation of the acrylate anion or styryl substituents increases the reactivity of the phenolate species towards electron transfer-initiated chemiexcitation. However, the stability of the phenol-dioxetanes was not impaired. We found that even the unprotected phenols (*i.e.* luminophores **2**, **3a–c**) are stable for days at room temperature, or for months at –20 °C, with only negligible decomposition. NAD(P)H probes that are based on these dioxetanes found to be stable at aqueous solution (pH 7.4) for several weeks at 4 °C. In addition, these compounds were found to be stable under normal room illumination conditions.

The aqueous solubility of our phenoxy-adamantyl-dioxetane probes is significantly increased, by the presence of a substituent with ionizable carboxylic acid. In the current work we describe a test-tube assay for NADH, in which high probe concentration, of 50 μM, is required to increase the assay sensitivity. In such assay, relatively high DMSO percentage (5%) can be used. For using dioxetane probes in living cells or animals, typically much lower probe concentration is applied (1–5 μM). Under such conditions, biologically compatible concentration of DMSO (0.1%), is sufficient for carboxylate-substituted dioxetane probes, to exhibit satisfactory aqueous solubility.

We believe that the importance of this work extends even beyond the report on the specific phenoxy-dioxetanes with rapid chemiexcitation. The significance of chemiexcitation kinetics as a key parameter was addressed here for the first time. Furthermore, by placing a rational method to control chemiexcitation rate, this work paves the way for the design of other luminophores with higher degree of control over their chemiexcitation properties.

## Conclusions

In summary, we were able to design and synthesize new phenoxy-dioxetanes with up to 100-fold more rapid chemiexcitation kinetics. Our rational design was further supported by computational analysis of the key structural elements. Chemiluminescent probes based on such phenoxy-dioxetanes proved up to 16-fold more sensitive than probes with slower chemiexcitation. Furthermore, the insights placed here regarding the relationship between dioxetane electronic structure and its chemiexcitation kinetics can assist in the design of other luminophores with higher degree of control over their characteristics. We anticipate that our new phenoxy-dioxetanes luminophores will serve as an ideal platform for designing more sensitive chemiluminescent probes for various bioanalytical applications.

## Conflicts of interest

There are no conflicts to declare.

## Supplementary Material

Supplementary information
