# Supplementary material for: Rapid chemiexcitation of phenoxy-dioxetane luminophores yields ultrasensitive chemiluminescence assays
Source: Chem Sci. 2018 Nov 19;10(5):1380–5. doi: 10.1039/c8sc04280b (PMC6354826; doi:10.1039/c8sc04280b)
Supplement: Supplementary file 1 [file SC-010-C8SC04280B-s001.pdf]

Electronic Supplementary Material (ESI) for Chemical Science.
This journal is © The Royal Society of Chemistry 2018

# Rapid Chemiexcitation of Phenoxy-Dioxetane Luminophores Yields Ultrasensitive Chemiluminescence Assays

Nir Hananya,+[a] Jolene P. Reid,+[b] Ori Green,[a] Matthew S. Sigman*[b] and Doron Shabat*[a]

[a] School of Chemistry, Raymond and Beverly Sackler Faculty of Exact Sciences, Tel Aviv University, Tel Aviv 69978, Israel

[b] Department of Chemistry, University of Utah, 315 South 1400 East, Salt Lake City, Utah 84112, United States

+ Equal contribution

## Supporting Information


- Chemiluminescence measurements protocols ........ S2
- Kinetic data of luminophores ........ S3-S6
- Chemiluminescence activation of NAD(P)H probes by NADH ........ S7-S8
- Chemiluminescence spectra ........ S9-S11
- Photophysical properties of benzoates ........ S12-S13
- Full list of authors in the Gaussian09 reference ........ S14
- Computational methods ........ S15
- Hammett Correlations ........ S16
- Cartesian coordinates of the benzoic acids ........ S17-S23
- Cartesian coordinates, energies (in Hartrees), number of imaginary frequencies of the phenols and phenoxy radicals ........ S24-S34
- Atomic spin densities ........ S35-S36
- Synthetic schemes and experimental procedures ........ S37-S55
- NMR and MS spectra of key compounds ........ S56-S66
- References ........ S67

## Chemiluminescence measurements protocols

All absorbance, fluorescence and chemiluminescence were recorded on Molecular Devices SpectraMax i3x.

Chemiluminescence kinetic profiles of luminophores **1a-c**, **2**, and **3a-c**:

Measurements were performed at 1 µM concentration, 10% DMSO in PBS, pH 7.4, or in sodium carbonate buffer [100 mM], pH 10. All measurements were performed at 25°C. 20 µL of luminophore solution in DMSO [10 µM] were injected to 180 µL of aqueous buffer using SpectraMax Injector Cartridge, and chemiluminescence was measured immediately.

Chemiluminescence quantum yield: measurements were performed under the same conditions that were used for the kinetic profile measurement. The quantum yield was determined based on the total light emitted from each luminophore, compared to the known standard luminophore **1b**. As expected, quantum yield was not affected by solution pH.

Chemiluminescence detection of NADH by NAD(P)H probes **Nt-NCL**, **Ac-NCL** and **St-NCL**:

DT Diaphorase (NQO1) human was purchased from Sigma-Aldrich (SKU - D1315). All aqueous solutions were composed of pH 7.4, 0.1 M PBS/0.1 M KCl/0.007% BSA according to Silvers et. al.[1] For NADH detection experiments, 10 µL of NADH solution at specific concentration were added to 190 µL solution of probe [50 µM] and diaphorase enzyme [20 µg/mL], and chemiluminescence was measured immediately. The maximal signal/background ratio was recorded, which in all cases was obtained after 30-60 s. LOD for the three probes was defined as blank + 3SD. For all probes, the aqueous buffer contained 5% DMSO. For **Nt-NCL**, solution contained also 20% of Sapphire-II™ Enhancer solution (Invitrogen™), to assist solubility.

## Kinetic data of luminophores

As expected, the decomposition profile of all luminophores described in this paper followed first-order kinetics; thus the rate constant, as well as $t_{1/2}$, were derived from the linear graph of ln (RLU) Vs time. RLU was normalized to facilitate comparison between graphs. The half-life was further evaluated by measuring the total light emission of each luminophore, and calculating the time required for the emission of half of the photons. In each case, $t_{1/2}$ determined by this method matched $t_{1/2}$ derived from the slope of ln (RLU) Vs time.

**1a**

pH 7.4: $K_{chemiexcitation}$ = 1.8×10$^{-3}$ s$^{-1}$, $t_{1/2}$ = 380 s.

pH 10: $K_{chemiexcitation}$ = 3.5×10$^{-3}$ s$^{-1}$, $t_{1/2}$ = 200 s.

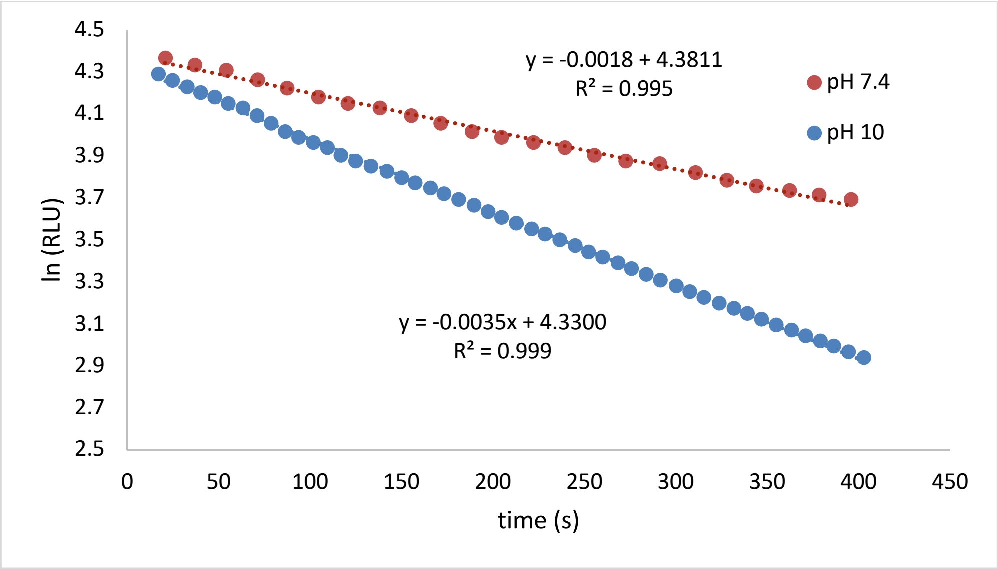

**1b**

pH 7.4: $K_{chemiexcitation}$ = 1.5×10$^{-3}$ s$^{-1}$, $t_{1/2}$ = 460 s.

pH 10: $K_{chemiexcitation}$ = 1.6×10$^{-3}$ s$^{-1}$, $t_{1/2}$ = 420 s.

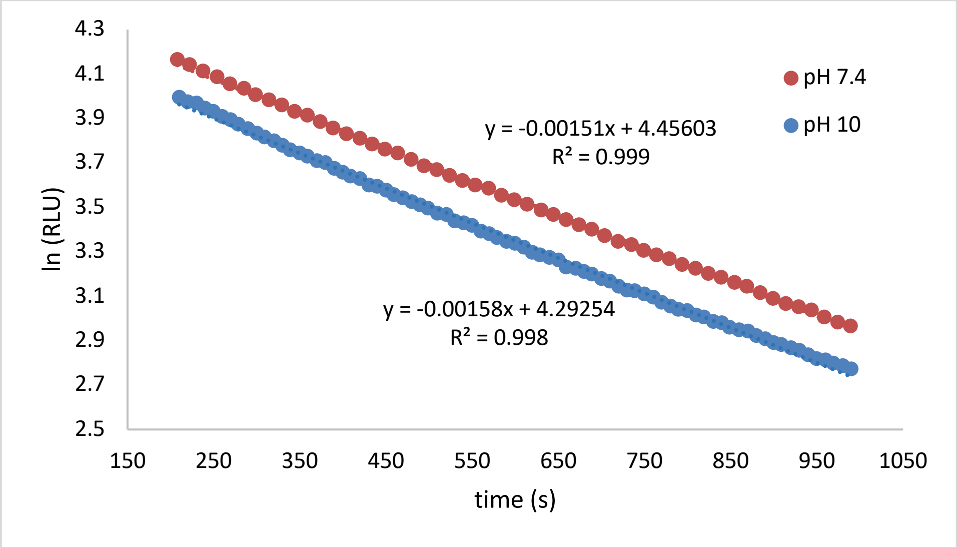


**1c**

pH 7.4: $K_{chemiexcitation}$ = 6.4×10$^{-4}$ s$^{-1}$, $t_{1/2}$ = 1100 s.

pH 10: $K_{chemiexcitation}$ = 7.2×10$^{-4}$ s$^{-1}$, $t_{1/2}$ = 960 s.

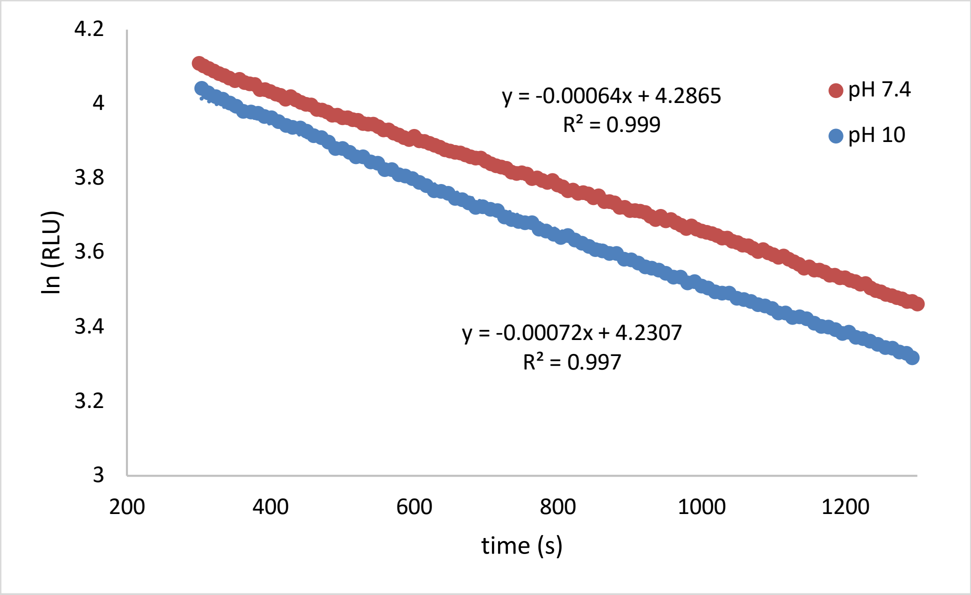

**2**

pH 7.4: $K_{chemiexcitation}$ = 8.1×10$^{-3}$ s$^{-1}$, $t_{1/2}$ = 85 s.

pH 10: $K_{chemiexcitation}$ = 1.2×10$^{-2}$ s$^{-1}$, $t_{1/2}$ = 60 s.

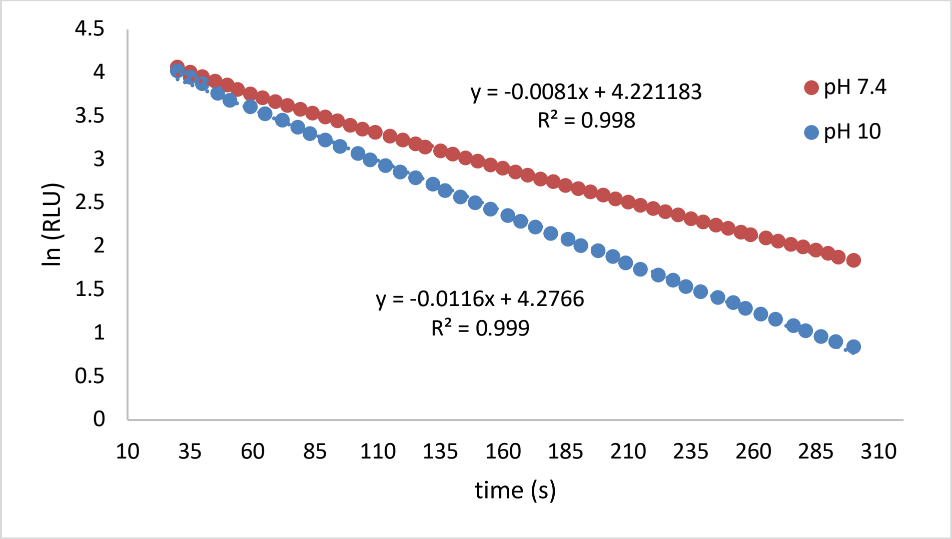


**3a**

pH 7.4: $K_{chemiexcitation}$ = 8.3×10$^{-2}$ s$^{-1}$, $t_{1/2}$ = 8.4 s.

pH 10: $K_{chemiexcitation}$ = 1.0×10$^{-1}$ s$^{-1}$, $t_{1/2}$ = 7.0 s.

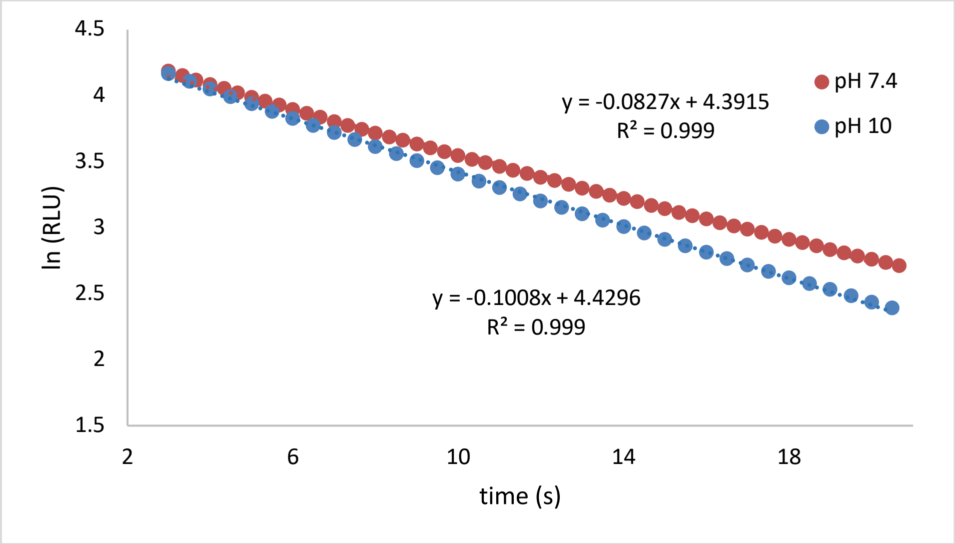

**3b**

pH 7.4: $K_{chemiexcitation}$ = 1.4×10$^{-1}$ s$^{-1}$, $t_{1/2}$ = 5.1 s.

pH 10: $K_{chemiexcitation}$ = 1.6×10$^{-1}$ s$^{-1}$, $t_{1/2}$ = 4.2 s.

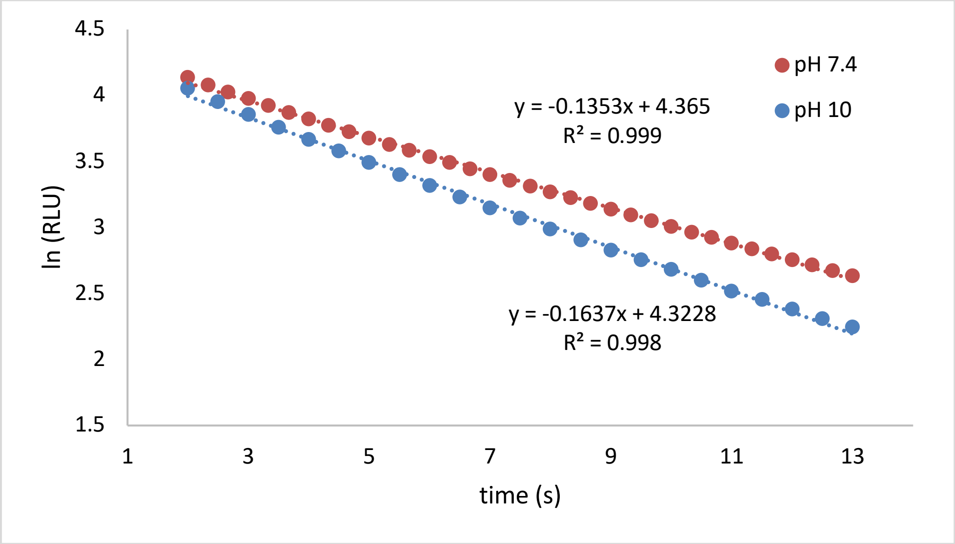


**3c**

pH 7.4: $K_{chemiexcitation}$ = 2.2×10$^{-1}$ s$^{-1}$, $t_{1/2}$ = 3.2 s.

pH 10: $K_{chemiexcitation}$ = 2.6×10$^{-1}$ s$^{-1}$, $t_{1/2}$ = 2.7 s.

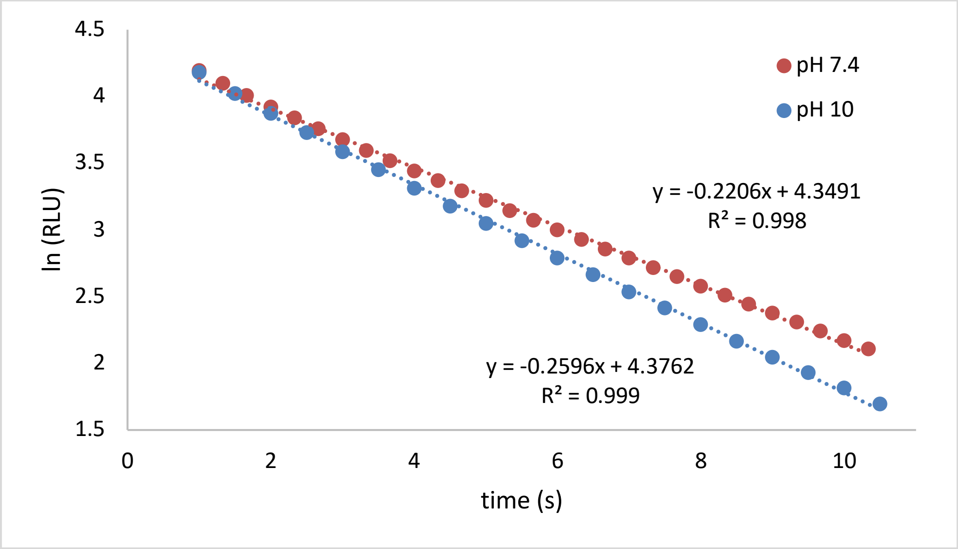

## Chemiluminescence activation of NAD(P)H probes by NADH

According to the protocol described above, we activated each probe with varying NADH concentrations and measured the chemiluminescent response. In each case the background signal was constant and stable. Due to the high rate of the enzymatic reduction of the probes by NADH, the maximal signal following NADH addition was always obtained during the first 60 s of measurement. The maximal S/B was recorded and used as data for Figure 3. LOD for the three probes was defined as blank + 3SD.

**Nt-NCL:**

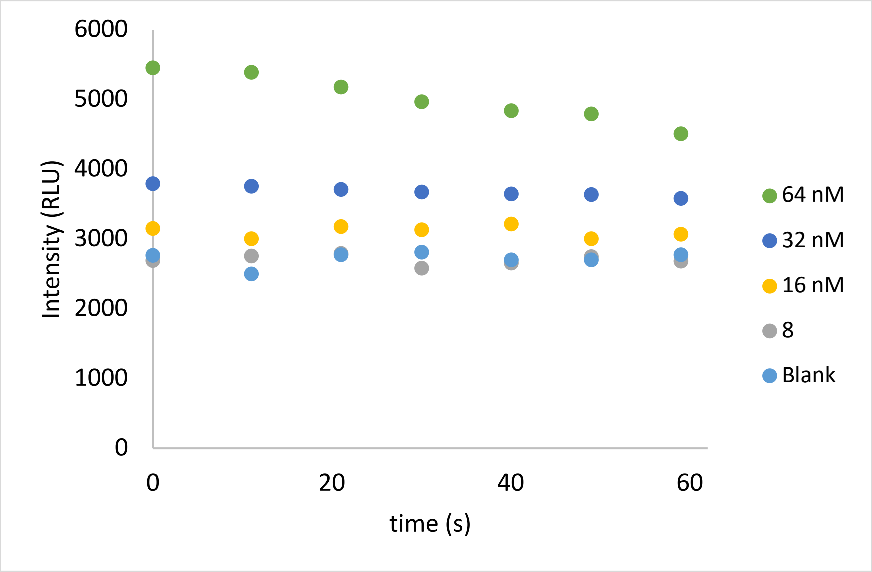


**Ac-NCL:**

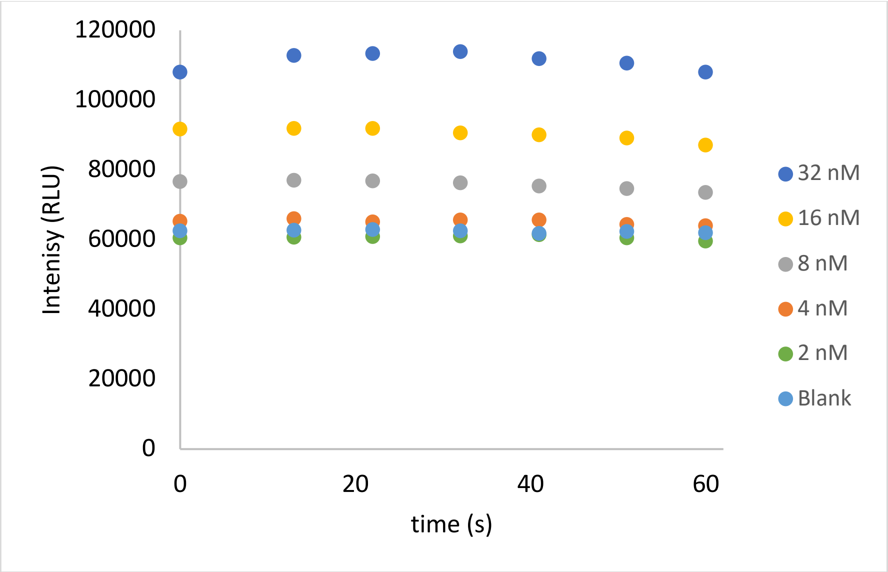

**St-NCL**

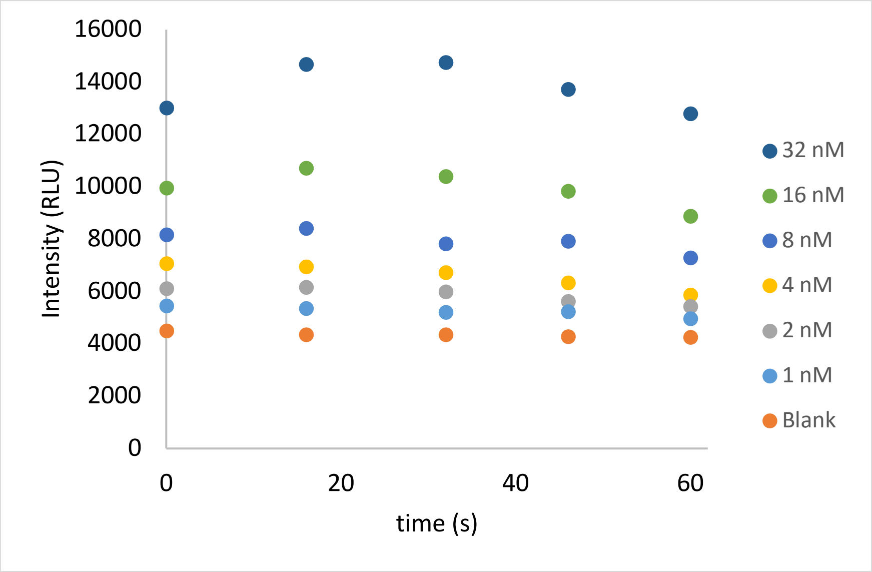

16000
14000
12000
10000
8000
6000
4000
2000
0
Intensity (RLU)
0
20
40
60
time (s)
32 nM
16 nM
8 nM
4 nM
2 nM
1 nM
Blank

## Chemiluminescence Spectra

### 1a

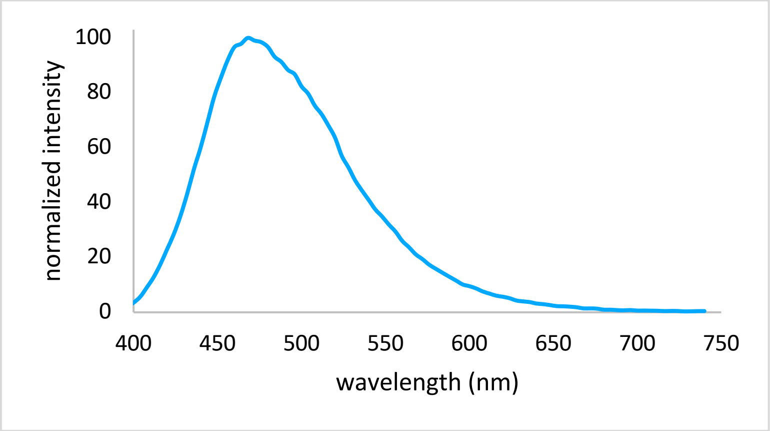


### 1b

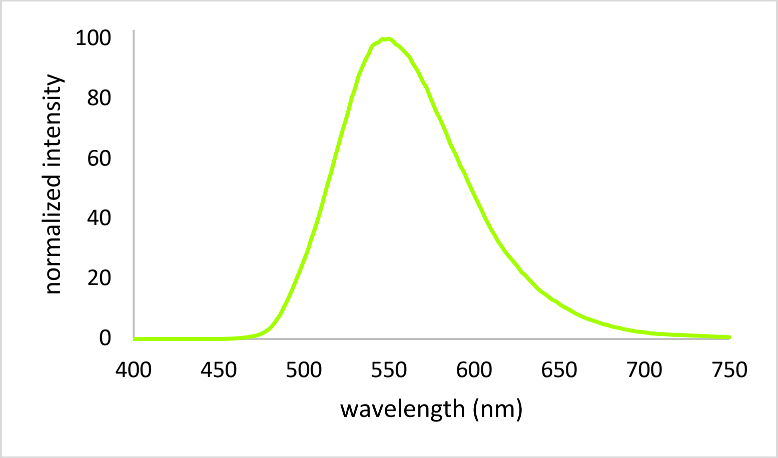


### 1c

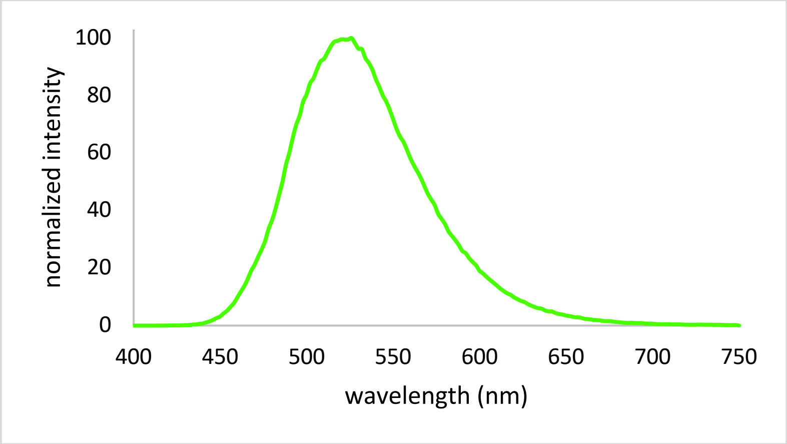

**2**

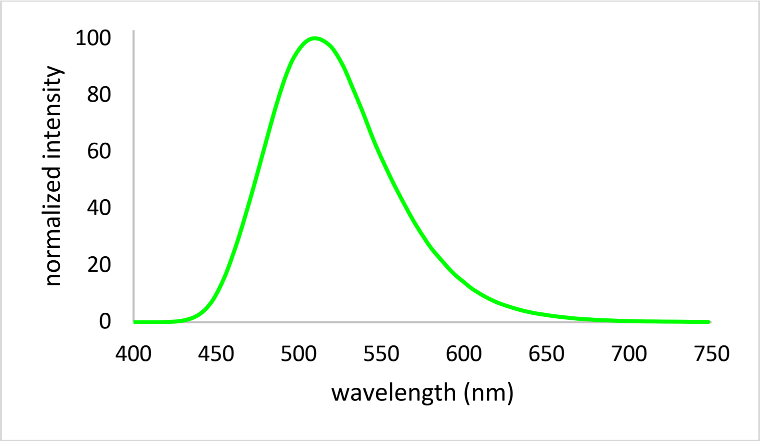

normalized intensity
100
80
60
40
20
0
400
450
500
550
600
650
700
750
wavelength (nm)


**3a**

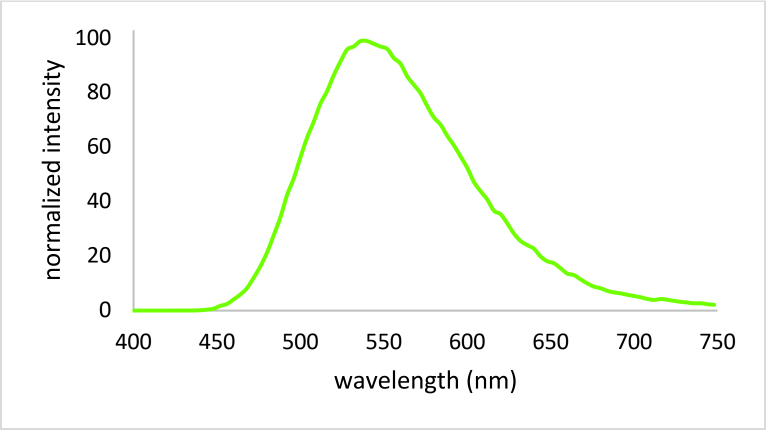

normalized intensity
100
80
60
40
20
0
400
450
500
550
600
650
700
750
wavelength (nm)


**3b**

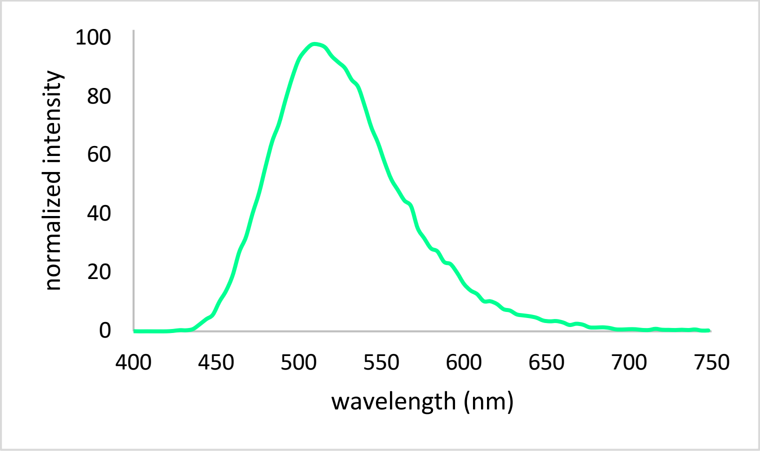

normalized intensity
100
80
60
40
20
0
400
450
500
550
600
650
700
750
wavelength (nm)

**3c**

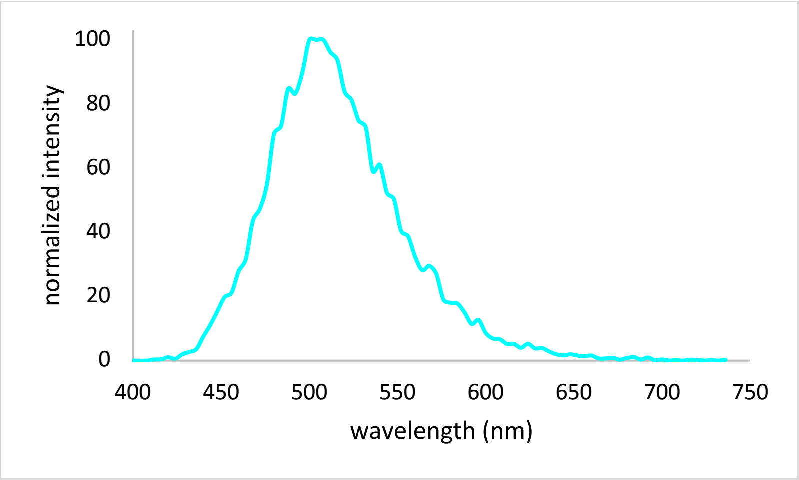

normalized intensity
100
80
60
40
20
0
400
450
500
550
600
650
700
750
wavelength (nm)

## Photophysical properties of benzoates 7a-f

The brightness of dioxetane-luminophores is closely dependent on the fluorescence quantum yield of the benzoates that correspond to the actual light-emitting species in the chemiluminescence process (see Scheme 1 in the manuscript). Therefore, the appropriate benzoates were synthesized and their fluorescence quantum yields were measured (see below for synthetic details). The fluorescence quantum yields of the styryl-substituted benzoates remained satisfactory ($\Phi$ = 0.16-0.38), in the same range of the acryl-substituted benzoates, thus affording efficient chemiluminescence process under physiological conditions.

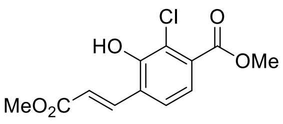


**7a**

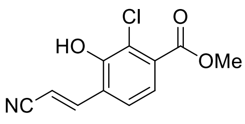


**7b**

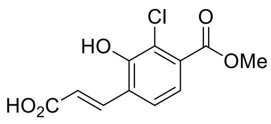


**7c**

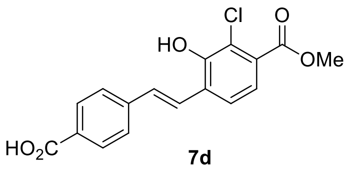


**7d**

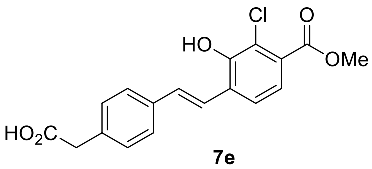


**7e**

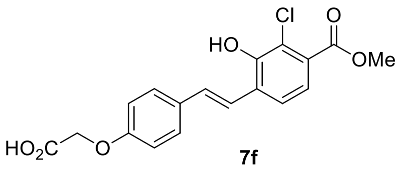


**7f**

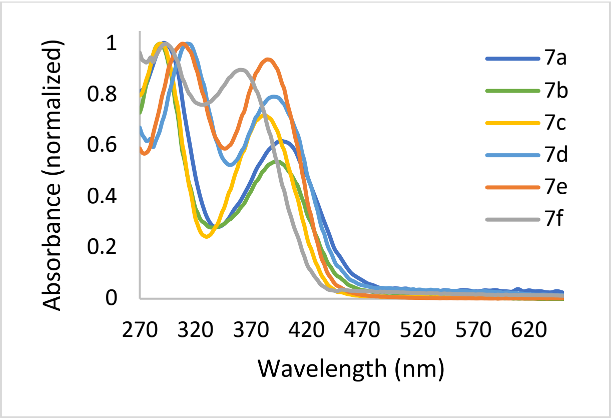


**Figure S1**. Normalized absorbance spectra of benzoates **7a-f** [50 µM]. Spectra were measured at sodium carbonate buffer [100 mM], pH 10, 5% DMSO.

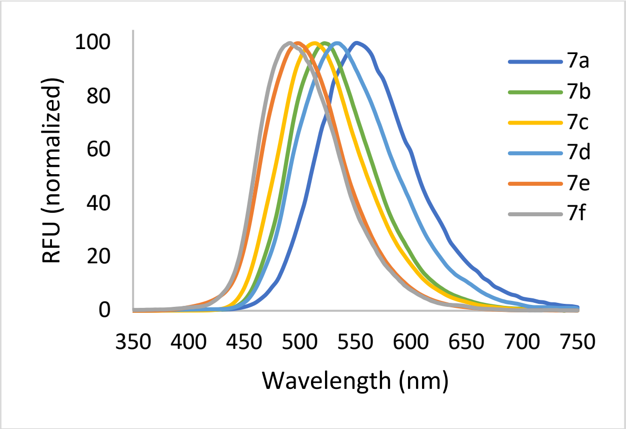


**Figure S2**. Normalized fluorescence spectra of benzoates **7a-f** [50 µM]. Spectra were measured at sodium carbonate buffer [100 mM], pH 10, 5% DMSO. Performing the measurements at pH 7.4 result in identical spectra.

**Table S1.** Photophysical properties of benzoates **7a-f.**

| | $\lambda_{max, abs}$ (nm) | $\lambda_{max, em}$ (nm) | Φ |
|---|---|---|---|
| **7a** | 290 | 550 | 0.08 |
| **7b** | 288 | 525 | 0.40 |
| **7c** | 288 | 510 | 0.44 |
| **7d** | 314 | 535 | 0.38 |
| **7e** | 310 | 500 | 0.25 |
| **7f** | 294 | 490 | 0.16 |

Fluorescence quantum yield was measured by Hamamatsu Quantaurus-QY spectrometer. Fluorescence quantum yield was the same in both pH 7.4 and 10.

**Full list of authors in the Gaussian09 reference**

M. J. Frisch, G. W. Trucks, H. B. Schlegel, G. E. Scuseria, M. A. Robb, J. R. Cheeseman, G. Scalmani, V. Barone, B. Mennucci, G. A. Petersson, H. Nakatsuji, M. Caricato, X. Li, H. P. Hratchian, A. F. Izmaylov, J. Bloino, G. Zheng, J. L. Sonnenberg, M. Hada, M. Ehara, K. Toyota, R. Fukuda, J. Hasegawa, M. Ishida, T. Nakajima, Y. Honda, O. Kitao, H. Nakai, T. Vreven, J. A. Montgomery, Jr., J. E. Peralta, F. Ogliaro, M. Bearpark, J. J. Heyd, E. Brothers, K. N. Kudin, V. N. Staroverov, T. Keith, R. Kobayashi, J. Normand, K. Raghavachari, A. Rendell, J. C. Burant, S. S. Iyengar, J. Tomasi, M. Cossi, N. Rega, J. M. Millam, M. Klene, J. E. Knox, J. B. Cross, V. Bakken, C. Adamo, J. Jaramillo, R. Gomperts, R. E. Stratmann, O. Yazyev, A. J. Austin, R. Cammi, C. Pomelli, J. W. Ochterski, R. L. Martin, K. Morokuma, V. G. Zakrzewski, G. A. Voth, P. Salvador, J. J. Dannenberg, S. Dapprich, A. D. Daniels, O. Farkas, J. B. Foresman, J. V. Ortiz, J. Cioslowski, and D. J. Fox, Gaussian, Inc., Wallingford CT, 2013.

## Computational Methods

Benzoic acids were optimized in the gas-phase with the M06-2X density functional,[2] and the triple-ζ valence quality def2-TZVP basis set of Weigend and Ahlrichs,[3] as implemented in Gaussian 09 (revision D.01).[4] NBO charges were calculated from these structures using NBO6,[5] at the same level. Hammett values were taken from the compilation made by Hansch, Leo and Taft.[6]

For the bond dissociation energy calculations (BDE)[7] all optimizations were performed in solution using the SMD[8] solvation model [water (ε =78.3553)] with spin-unrestricted DFT using UM06-2X and the triple-ζ valence quality def2-TZVP basis set, with non-default convergence and grid criteria (tight convergence, ultrafine grid density) as implemented in Gaussian 09 (revision D.01). The energy of the H atom was calculated to be -0.495999 Hartrees at this level. For our calculations we used truncated probe structures, focusing on the conserved phenol conjugated π-system wherein the adamantane-dioxetane portion was replaced with a hydrogen. The population of atomic spin densities were obtained via Mulliken population analysis and NBO analysis using NBO6.[5] Conformational searches were performed with Macromodel version 11.7[9] and the OPLS3 force field.[10]

## Compilation of Parameters of Various Substituted Benzoic Acids

**Table S2.** Comparison of Hammett Values and $NBO_{C=O}$

| Para R | $\sigma_p$ | $NBO_{C=O}$ |
|---|---|---|
| 4-OMe | -0.27 | 0.781 |
| 4-$^t$Bu | -0.20 | 0.781 |
| H | 0.00 | 0.780 |
| 4-$N_3$ | 0.08 | 0.779 |
| 4-Cl | 0.23 | 0.779 |
| 4-Br | 0.23 | 0.779 |
| 4-$C_6F_5$ | 0.27 | 0.778 |
| 4-CN | 0.66 | 0.776 |
| 4-$NO_2$ | 0.78 | 0.776 |
| 4-NO | 0.91 | 0.775 |

Hammett values have typically been employed to describe the effect of electron withdrawing and donating substituents on experimental observations. Previous efforts have shown that empirically derived Hammett values, $\sigma_p$, can be replaced by computational equivalents such as IR C=O stretching frequencies and NBO charges.[11] In our analysis we employed the $NBO_{C=O}$, the charge on the benzoic acid moiety due to the near perfect correlation between the values (Figure S3).

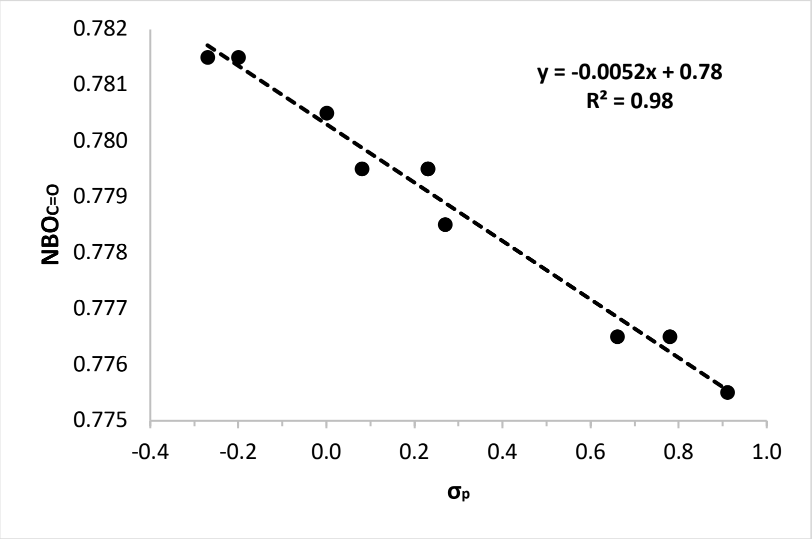


**Figure S3.** Comparison of Hammett Values and $NBO_{C=O}$.

## Benzoic Acids

### 4-methoxybenzoic acid

M06-2X/def2-TZVP Geometry

| | | | | | | | |
|---|---|---|---|---|---|---|---|
| C | -1.65980 | 0.28900 | -0.00010 | C | -3.87140 | -0.56050 | 0.00020 |
| C | -0.82930 | 1.41270 | -0.00010 | H | -1.28890 | 2.39170 | -0.00000 |
| C | 0.53960 | 1.25950 | -0.00010 | H | 1.18300 | 2.12850 | -0.00000 |
| C | 1.10370 | -0.01760 | -0.00010 | H | 0.72510 | -2.11400 | -0.00010 |
| C | 0.27410 | -1.13010 | -0.00010 | H | -1.72890 | -1.87060 | -0.00010 |
| C | -1.10430 | -0.98970 | -0.00010 | H | 4.20640 | 0.66120 | 0.00030 |
| C | 2.56670 | -0.23580 | 0.00000 | H | -4.87370 | -0.14110 | 0.00040 |
| O | 3.27250 | 0.91320 | 0.00010 | H | -3.73390 | -1.17640 | -0.89230 |
| O | 3.10450 | -1.31090 | 0.00010 | H | -3.73360 | -1.17650 | 0.89260 |
| O | -2.98590 | 0.53930 | 0.00000 | | | | |

### 4-(*tert*-butyl)benzoic acid

M06-2X/def2-TZVP Geometry

| | | | | | | | |
|---|---|---|---|---|---|---|---|
| C | 1.04630 | 0.03070 | 0.00000 | H | -1.64530 | 2.11000 | -0.00010 |
| C | 0.30340 | 1.20880 | -0.00000 | H | -1.57160 | -2.15440 | 0.00010 |
| C | -1.08490 | 1.18520 | -0.00010 | H | 0.89190 | -2.11660 | 0.00010 |
| C | -1.75900 | -0.02730 | -0.00000 | H | -4.79020 | 0.93610 | 0.00020 |
| C | -1.03340 | -1.21550 | 0.00000 | H | 2.71950 | -1.74970 | -1.28070 |
| C | 0.34670 | -1.18120 | 0.00000 | H | 2.72390 | -0.21740 | -2.15810 |
| C | -3.24010 | -0.10900 | -0.00000 | H | 4.16290 | -0.73400 | -1.26540 |
| O | -3.83670 | 1.09900 | 0.00010 | H | 2.71860 | -1.74860 | 1.28230 |
| O | -3.87100 | -1.13130 | -0.00010 | H | 4.16290 | -0.73430 | 1.26530 |
| C | 2.57300 | 0.01650 | -0.00000 | H | 2.72480 | -0.21540 | 2.15820 |
| C | 3.07110 | -0.71810 | -1.25260 | H | 2.86880 | 1.98790 | -0.88660 |
| C | 3.07120 | -0.71730 | 1.25300 | H | 2.86920 | 1.98820 | 0.88560 |
| C | 3.16710 | 1.42460 | -0.00040 | H | 4.25600 | 1.35540 | -0.00060 |
| H | 0.80240 | 2.16730 | -0.00000 | | | | |

### Benzoic Acid

M06-2X/def2-TZVP Geometry

| | | | | | | | |
|---|---|---|---|---|---|---|---|
| C | 2.55390 | -0.04500 | 0.00000 | C | -0.21410 | 0.03000 | 0.00000 |
| C | 1.82970 | -1.22980 | 0.00010 | C | 0.51130 | 1.21730 | -0.00010 |
| C | 0.44370 | -1.19580 | 0.00010 | C | 1.89540 | 1.17850 | -0.00010 |

| | | | | | | | |
|---|---|---|---|---|---|---|---|
| C | -1.69750 | 0.12280 | 0.00000 | H | -0.02820 | 2.15530 | -0.00010 |
| O | -2.30160 | -1.08050 | -0.00020 | H | 2.46260 | 2.09990 | -0.00010 |
| O | -2.31920 | 1.15000 | 0.00020 | H | -3.25440 | -0.91290 | 0.00020 |
| H | 2.34610 | -2.18060 | 0.00010 | H | 3.63600 | -0.07500 | 0.00010 |
| H | -0.13090 | -2.11150 | 0.00010 | | | | |

**4-azidobenzoic acid**

M06-2X/def2-TZVP Geometry

| | | | | | | | |
|---|---|---|---|---|---|---|---|
| C | 0.91690 | -0.85380 | -0.00010 | O | -3.25570 | -1.43440 | 0.00010 |
| C | -0.44580 | -1.08410 | -0.00010 | N | 3.55630 | -0.14880 | 0.00010 |
| C | -1.34050 | -0.01940 | -0.00010 | N | 4.35140 | -0.93190 | 0.00010 |
| C | -0.86020 | 1.28820 | -0.00000 | H | 1.60620 | -1.68900 | -0.00010 |
| C | 0.50000 | 1.52810 | -0.00000 | H | -0.83720 | -2.09290 | -0.00010 |
| C | 1.39090 | 0.45760 | -0.00010 | H | -1.55750 | 2.11440 | 0.00000 |
| N | 2.76350 | 0.79390 | 0.00000 | H | 0.89420 | 2.53500 | 0.00000 |
| C | -2.79050 | -0.32710 | 0.00000 | H | -4.48130 | 0.47210 | 0.00030 |
| O | -3.56290 | 0.77590 | 0.00000 | | | | |

**4-chlorobenzoic**

M06-2X/def2-TZVP Geometry

| | | | | | | | |
|---|---|---|---|---|---|---|---|
| C | -1.67880 | -0.00380 | 0.00000 | H | -1.52650 | -2.13890 | 0.00010 |
| C | -0.98370 | -1.20420 | 0.00010 | H | 0.95940 | -2.10700 | 0.00010 |
| C | 0.40110 | -1.18130 | 0.00000 | H | 0.91390 | 2.16200 | -0.00000 |
| C | 1.07760 | 0.03380 | 0.00000 | H | -1.58520 | 2.13600 | -0.00010 |
| C | 0.36600 | 1.22900 | -0.00000 | H | 4.10290 | -0.95240 | 0.00010 |
| C | -1.01710 | 1.21640 | -0.00000 | Cl | -3.40890 | -0.02780 | -0.00000 |
| C | 2.56120 | 0.10740 | 0.00000 | | | | |
| O | 3.14740 | -1.10390 | -0.00010 | | | | |
| O | 3.19370 | 1.12760 | 0.00010 | | | | |

**4-bromobenzoic acid**

M06-2X/def2-TZVP Geometry

| | | | | | | | |
|---|---|---|---|---|---|---|---|
| C | 0.99510 | 0.00540 | 0.00000 | C | 0.33050 | 1.22370 | 0.00000 |
| C | 0.30460 | -1.19730 | 0.00000 | C | -3.24510 | 0.10350 | -0.00000 |
| C | -1.08090 | -1.17840 | 0.00000 | O | -3.82720 | -1.10950 | 0.00000 |
| C | -1.76090 | 0.03450 | -0.00000 | O | -3.88040 | 1.12190 | -0.00000 |
| C | -1.05330 | 1.23180 | 0.00000 | Br | 2.88590 | -0.01560 | -0.00000 |

| | | | | | | | |
|---|---|---|---|---|---|---|---|
| H | 0.84520 | -2.13320 | 0.00000 | H | 0.89100 | 2.14790 | 0.00000 |
| H | -1.63620 | -2.10610 | -0.00000 | H | -4.78340 | -0.96160 | -0.00020 |
| H | -1.60380 | 2.16330 | 0.00000 | | | | |

**2',3',4',5',6'-pentafluoro-[1,1'-biphenyl]-4-carboxylic acid**

M06-2X/def2-TZVP Geometry

| | | | | | | | |
|---|---|---|---|---|---|---|---|
| C | 1.35550 | -0.97060 | 0.74710 | F | -3.61080 | -2.30450 | -0.36900 |
| C | 2.73780 | -0.97610 | 0.75320 | F | -4.95550 | 0.02030 | -0.00940 |
| C | 3.44020 | -0.02810 | 0.01780 | F | -3.58500 | 2.32890 | 0.35820 |
| C | 2.75510 | 0.92750 | -0.72390 | F | -0.91720 | 2.32410 | 0.37590 |
| C | 1.37100 | 0.93450 | -0.72660 | C | 4.92620 | -0.07670 | 0.05690 |
| C | 0.66030 | -0.01360 | 0.00740 | O | 5.50270 | 0.88710 | -0.68330 |
| C | -0.82120 | -0.00460 | 0.00260 | O | 5.56510 | -0.88120 | 0.67690 |
| C | -1.56040 | -1.16980 | -0.17530 | H | 0.80940 | -1.70760 | 1.32010 |
| C | -2.94390 | -1.17430 | -0.18340 | H | 3.29170 | -1.70900 | 1.32470 |
| C | -3.63300 | 0.01240 | -0.00550 | H | 3.30640 | 1.66080 | -1.29570 |
| C | -2.93060 | 1.19060 | 0.17640 | H | 0.83700 | 1.67740 | -1.30360 |
| C | -1.54740 | 1.16930 | 0.17640 | H | 6.45960 | 0.77410 | -0.59440 |
| F | -0.94300 | -2.33180 | -0.37120 | | | | |

**4-cyanobenzoic acid**

M06-2X/def2-TZVP Geometry

| | | | | | | | |
|---|---|---|---|---|---|---|---|
| C | 1.80890 | -0.00380 | 0.00000 | C | 3.24570 | -0.02400 | -0.00000 |
| C | 1.10370 | -1.20570 | -0.00000 | N | 4.39270 | -0.04090 | -0.00010 |
| C | -0.27990 | -1.18510 | -0.00000 | H | 1.64360 | -2.14260 | -0.00010 |
| C | -0.95220 | 0.03230 | 0.00000 | H | -0.84100 | -2.10880 | -0.00010 |
| C | -0.24620 | 1.23070 | 0.00010 | H | -0.79800 | 2.16110 | 0.00010 |
| C | 1.13580 | 1.21760 | 0.00010 | H | 1.70000 | 2.13990 | 0.00010 |
| C | -2.44070 | 0.10580 | -0.00000 | H | -3.98010 | -0.95960 | -0.00010 |
| O | -3.02320 | -1.10450 | 0.00010 | | | | |
| O | -3.06720 | 1.12820 | -0.00020 | | | | |

**4-nitrobenzoic acid**

M06-2X/def2-TZVP Geometry

| | | | | | | | |
|---|---|---|---|---|---|---|---|
| C | 1.39040 | 0.00030 | 0.00000 | C | -1.34110 | 0.03390 | 0.00010 |
| C | 0.71560 | -1.20640 | 0.00000 | C | -0.63770 | 1.23400 | 0.00010 |
| C | -0.66940 | -1.18420 | 0.00000 | C | 0.74570 | 1.22450 | 0.00010 |

| | | | |
|---|---|---|---|
| C | -2.83100 | 0.10610 | -0.00000 |
| O | -3.41150 | -1.10460 | 0.00030 |
| O | -3.45710 | 1.12830 | -0.00040 |
| H | 1.27210 | -2.13160 | -0.00000 |
| H | -1.23020 | -2.10780 | -0.00000 |
| H | -1.19070 | 2.16350 | 0.00010 |
| H | 1.32500 | 2.13580 | 0.00020 |
| H | -4.36880 | -0.96190 | -0.00020 |
| N | 2.87010 | -0.01850 | -0.00000 |
| O | 3.41270 | -1.09850 | -0.00030 |
| O | 3.43940 | 1.04750 | 0.00010 |

**4-nitrosobenzoic acid**

M06-2X/def2-TZVP Geometry

| | | | |
|---|---|---|---|
| C | -0.96510 | 1.36730 | -0.00010 |
| C | 0.41650 | 1.25090 | -0.00040 |
| C | 0.99090 | -0.01460 | -0.00010 |
| C | 0.19680 | -1.16300 | 0.00020 |
| C | -1.17820 | -1.04960 | 0.00040 |
| C | -1.74340 | 0.22140 | 0.00020 |
| N | -3.17720 | 0.45080 | 0.00060 |
| C | 2.47020 | -0.20070 | -0.00010 |
| O | 3.14420 | 0.96150 | 0.00020 |
| O | 3.01850 | -1.26710 | -0.00030 |
| O | -3.84070 | -0.54210 | -0.00070 |
| H | -1.45680 | 2.33180 | 0.00000 |
| H | 1.04690 | 2.12870 | -0.00060 |
| H | 0.68380 | -2.12920 | 0.00050 |
| H | -1.82280 | -1.91820 | 0.00060 |
| H | 4.08670 | 0.74170 | 0.00070 |

**(*E*)-4-(2-cyanovinyl)benzoic acid**

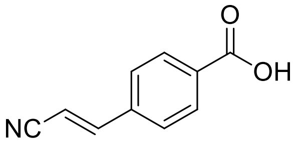


M06-2X/def2-TZVP Geometry

| | | | |
|---|---|---|---|
| C | -0.07680 | 1.37350 | 0.00010 |
| C | 1.30210 | 1.24670 | -0.00000 |
| C | 1.87400 | -0.01890 | 0.00000 |
| C | 1.06260 | -1.15190 | 0.00010 |
| C | -0.31040 | -1.02020 | 0.00020 |
| C | -0.90130 | 0.24760 | 0.00010 |
| C | -2.35140 | 0.45080 | 0.00020 |
| C | -3.28320 | -0.50600 | -0.00020 |
| C | -4.67510 | -0.18640 | -0.00010 |
| N | -5.79680 | 0.05980 | -0.00010 |
| C | 3.34810 | -0.21790 | -0.00010 |
| O | 4.03320 | 0.93880 | -0.00020 |
| O | 3.88930 | -1.28880 | 0.00000 |
| H | -0.52590 | 2.35900 | 0.00000 |
| H | 1.93440 | 2.12340 | -0.00010 |
| H | 1.53170 | -2.12700 | 0.00020 |
| H | -0.92480 | -1.91060 | 0.00030 |
| H | -2.68250 | 1.48410 | 0.00050 |
| H | -3.04070 | -1.56050 | -0.00060 |
| H | 4.97330 | 0.70900 | -0.00020 |

**(*E*)-4-(3-methoxy-3-oxoprop-1-en-1-yl)benzoic acid**

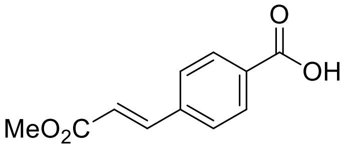


M06-2X/def2-TZVP Geometry

| | | | | | | | |
|---|---|---|---|---|---|---|---|
| C | -0.64860 | -0.95960 | -0.00000 | O | -5.07750 | 0.80030 | -0.00020 |
| C | -2.01430 | -1.15410 | -0.00010 | O | -4.83380 | -1.41790 | -0.00030 |
| C | -2.87660 | -0.05930 | -0.00010 | H | 0.00910 | -1.81830 | 0.00000 |
| C | -2.36190 | 1.23110 | -0.00000 | H | -2.43810 | -2.14960 | -0.00020 |
| C | -0.98990 | 1.42010 | 0.00010 | H | -3.03370 | 2.07800 | -0.00000 |
| C | -0.11440 | 0.33350 | 0.00010 | H | -0.58440 | 2.42440 | 0.00020 |
| C | 1.32660 | 0.59720 | 0.00020 | H | 1.62550 | 1.64180 | 0.00030 |
| C | 2.30450 | -0.30660 | 0.00000 | H | 2.13620 | -1.37470 | -0.00010 |
| C | 3.71450 | 0.14270 | 0.00020 | H | 6.18760 | 0.00760 | -0.88570 |
| O | 4.55100 | -0.90530 | -0.00010 | H | 6.18750 | 0.00720 | 0.88610 |
| O | 4.08730 | 1.28590 | 0.00040 | H | 6.46960 | -1.52120 | -0.00010 |
| C | 5.93730 | -0.57480 | 0.00010 | H | -6.00510 | 0.52510 | -0.00030 |
| C | -4.33900 | -0.32430 | -0.00020 | | | | |

**(*E*)-3-(4-carboxyphenyl)acrylate**

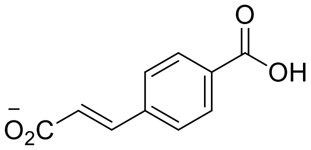


M06-2X/def2-TZVP Geometry

| | | | | | | | |
|---|---|---|---|---|---|---|---|
| C | 0.15500 | -0.96270 | -0.00040 | C | 3.81540 | -0.05660 | 0.00010 |
| C | 1.52780 | -1.09550 | -0.00030 | O | 4.27670 | -1.33080 | 0.00000 |
| C | 2.34650 | 0.03660 | -0.00000 | O | 4.58200 | 0.87450 | 0.00030 |
| C | 1.75930 | 1.29960 | 0.00010 | H | -0.46550 | -1.84870 | -0.00060 |
| C | 0.38410 | 1.42430 | -0.00000 | H | 1.97880 | -2.07880 | -0.00040 |
| C | -0.45560 | 0.30090 | -0.00020 | H | 2.40070 | 2.17170 | 0.00030 |
| C | -1.90510 | 0.49220 | -0.00030 | H | -0.06960 | 2.40800 | 0.00010 |
| C | -2.83910 | -0.45870 | 0.00030 | H | -2.26980 | 1.51620 | -0.00070 |
| C | -4.34610 | -0.14270 | 0.00010 | H | -2.59080 | -1.51640 | 0.00080 |
| O | -4.65150 | 1.06250 | -0.00020 | H | 5.24030 | -1.25890 | 0.00010 |
| O | -5.06690 | -1.15840 | 0.00030 | | | | |

**(*E*)-4-(4-carboxystyryl)benzoate**

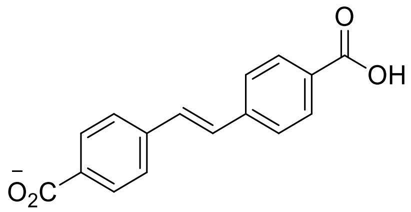


M06-2X/def2-TZVP Geometry

| | | | | | | | |
|---|---|---|---|---|---|---|---|
| C | -2.70660 | 1.44250 | 0.13630 | O | 6.74990 | 1.23950 | -0.12840 |
| C | -4.07990 | 1.30570 | 0.15470 | C | -6.12940 | -0.05760 | 0.03040 |
| C | -4.65820 | 0.04750 | 0.00810 | O | -6.89410 | 0.86230 | 0.17010 |
| C | -3.83520 | -1.06630 | -0.16130 | O | -6.57900 | -1.32350 | -0.12510 |
| C | -2.46310 | -0.92330 | -0.18020 | H | -2.26260 | 2.42390 | 0.25270 |
| C | -1.86150 | 0.33490 | -0.02420 | H | -4.72670 | 2.16410 | 0.28220 |
| C | -0.41770 | 0.54410 | -0.02920 | H | -4.28090 | -2.04400 | -0.28520 |
| C | 0.51660 | -0.41830 | -0.00270 | H | -1.84640 | -1.79860 | -0.33220 |
| C | 1.96360 | -0.24790 | -0.00810 | H | -0.10540 | 1.58290 | -0.03330 |
| C | 2.77260 | -1.37910 | 0.14510 | H | 0.19070 | -1.45430 | 0.04400 |
| C | 4.15390 | -1.27410 | 0.15510 | H | 2.29870 | -2.34910 | 0.26130 |
| C | 4.77020 | -0.03730 | 0.00750 | H | 4.79660 | -2.13760 | 0.27400 |
| C | 3.96960 | 1.09350 | -0.15180 | H | 4.47630 | 2.04320 | -0.27140 |
| C | 2.59120 | 0.99590 | -0.16090 | H | 1.99380 | 1.88980 | -0.29770 |
| C | 6.31650 | 0.08320 | 0.01360 | H | -7.54360 | -1.26900 | -0.09370 |
| O | 6.92240 | -0.99230 | 0.15920 | | | | |

**(*E*)-2-(4-(4-carboxystyryl)phenyl)acetate**

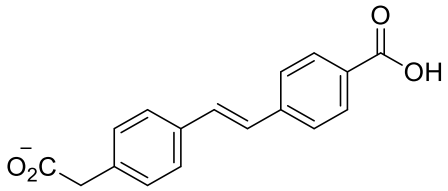


M06-2X/def2-TZVP Geometry

| | | | | | | | |
|---|---|---|---|---|---|---|---|
| C | 2.89850 | 1.01140 | -0.01010 | C | 0.80590 | -0.36950 | -0.28250 |
| C | 4.26830 | 1.08820 | 0.11620 | C | -0.09610 | 0.61400 | -0.13950 |
| C | 5.04800 | -0.06930 | 0.10080 | C | -1.54230 | 0.51480 | -0.25680 |
| C | 4.42630 | -1.30640 | -0.04710 | C | -2.31680 | 1.64970 | -0.00660 |
| C | 3.05210 | -1.37740 | -0.17320 | C | -3.69780 | 1.61100 | -0.10280 |
| C | 2.25110 | -0.22690 | -0.15230 | C | -4.36630 | 0.43940 | -0.45800 |

| | | | | | | | |
|---|---|---|---|---|---|---|---|
| C | -3.58810 | -0.70410 | -0.70200 | H | 2.57380 | -2.34290 | -0.28650 |
| C | -2.21430 | -0.66680 | -0.60520 | H | 0.46160 | -1.37580 | -0.49520 |
| C | -5.84980 | 0.38770 | -0.54450 | H | 0.26110 | 1.61060 | 0.10670 |
| C | -6.51400 | -0.57880 | 0.53200 | H | -1.82080 | 2.57460 | 0.26930 |
| O | -6.03890 | -1.72630 | 0.55980 | H | -4.27680 | 2.50370 | 0.10320 |
| O | -7.44010 | -0.06920 | 1.17520 | H | -4.10620 | -1.62580 | -0.93010 |
| C | 6.51000 | 0.06590 | 0.23810 | H | -1.65020 | -1.57060 | -0.80230 |
| O | 7.10740 | 1.10420 | 0.36340 | H | -6.14790 | -0.02780 | -1.51140 |
| O | 7.16320 | -1.11830 | 0.21180 | H | -6.28190 | 1.38110 | -0.43840 |
| H | 2.31920 | 1.92460 | -0.00920 | H | 8.10110 | -0.90660 | 0.31100 |
| H | 4.76360 | 2.04450 | 0.22550 | | | | |
| H | 5.02360 | -2.20790 | -0.06160 | | | | |

**(*E*)-2-(4-(4-carboxystyryl)phenoxy)acetate**

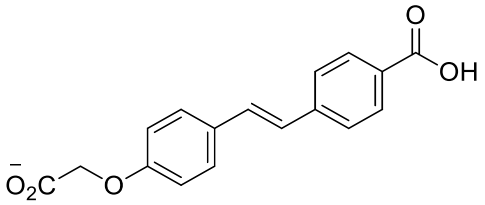


M06-2X/def2-TZVP Geometry

| | | | | | | | |
|---|---|---|---|---|---|---|---|
| C | 3.09560 | -0.88110 | -0.08960 | O | -7.15620 | -1.89960 | 0.01010 |
| C | 4.44260 | -1.17830 | -0.08640 | C | 6.83930 | -0.43280 | 0.05450 |
| C | 5.39020 | -0.16120 | 0.03940 | O | 7.14010 | -1.74550 | -0.06950 |
| C | 4.96000 | 1.15710 | 0.16320 | O | 7.70570 | 0.39720 | 0.16460 |
| C | 3.61110 | 1.44890 | 0.15810 | H | 2.38250 | -1.68960 | -0.17270 |
| C | 2.64180 | 0.44210 | 0.02780 | H | 4.77240 | -2.20450 | -0.17510 |
| C | 1.23290 | 0.81290 | 0.02970 | H | 5.70140 | 1.93920 | 0.26410 |
| C | 0.20060 | -0.01680 | -0.19690 | H | 3.28330 | 2.47700 | 0.25480 |
| C | -1.21420 | 0.30300 | -0.19540 | H | 1.03610 | 1.86160 | 0.22580 |
| C | -2.13750 | -0.69930 | -0.51030 | H | 0.41580 | -1.05890 | -0.41780 |
| C | -3.50070 | -0.47690 | -0.52870 | H | -1.76890 | -1.69410 | -0.73670 |
| C | -3.98970 | 0.79710 | -0.22260 | H | -4.17970 | -1.28950 | -0.73900 |
| C | -3.07690 | 1.81340 | 0.10620 | H | -3.47660 | 2.78800 | 0.35430 |
| C | -1.72470 | 1.57150 | 0.11930 | H | -1.05140 | 2.37720 | 0.38530 |
| O | -5.27150 | 1.14950 | -0.25400 | H | -6.01890 | -0.25520 | -1.57820 |
| C | -6.24150 | 0.15710 | -0.59000 | H | -7.18600 | 0.69530 | -0.64670 |
| C | -6.39730 | -1.01030 | 0.43300 | H | 8.10470 | -1.80140 | -0.04440 |
| O | -5.76850 | -0.90460 | 1.49380 | | | | |

## BDE Calculations

### Truncated Luminophores

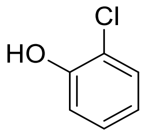

**1a**$_{truncated}$

UM06-2X/def2-TZVP Energy in solution = -767.068527

UM06-2X/def2-TZVP Zero-point energy corrected in solution = -766.972871

Number of Imaginary Frequencies = 0

SMD-M06-2X/def2-TZVP Geometry

| | | | | | | | |
|---|---|---|---|---|---|---|---|
| C | 2.34330 | 0.01510 | 0.00000 | O | -0.66590 | 2.04800 | -0.00000 |
| C | 1.82260 | -1.27400 | 0.00000 | H | 3.41460 | 0.16800 | 0.00000 |
| C | 0.44900 | -1.45910 | 0.00000 | H | 2.47940 | -2.13320 | 0.00000 |
| C | -0.39050 | -0.35670 | 0.00000 | H | 0.02110 | -2.45290 | 0.00000 |
| C | 0.12140 | 0.93890 | 0.00000 | H | 1.89090 | 2.12360 | 0.00000 |
| C | 1.50070 | 1.11400 | 0.00000 | H | -1.60100 | 1.79800 | -0.00000 |
| Cl | -2.11510 | -0.57400 | 0.00000 | | | | |

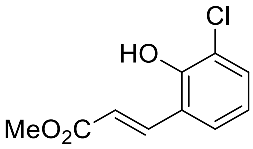

**1b**$_{truncated}$

UM06-2X/def2-TZVP Energy in solution = -1072.354886

UM06-2X/def2-TZVP Zero-point energy corrected in solution = -1072.181541

Number of Imaginary Frequencies = 0

SMD-M06-2X/def2-TZVP Geometry

| | | | | | | | |
|---|---|---|---|---|---|---|---|
| C | -1.20000 | 2.10090 | -0.00110 | C | -1.34210 | -0.30310 | -0.02130 |
| C | -2.57810 | 2.20880 | 0.01530 | C | -0.55330 | 0.86030 | -0.01690 |
| C | -3.35220 | 1.05810 | 0.01980 | Cl | -3.69360 | -1.62360 | 0.01710 |
| C | -2.73110 | -0.17720 | 0.00290 | C | 0.90550 | 0.86450 | -0.02180 |

| | | | | | | | |
|---|---|---|---|---|---|---|---|
| C | 1.76910 | -0.15620 | -0.00640 | H | -4.43260 | 1.11030 | 0.03660 |
| C | 3.21430 | 0.13000 | -0.00160 | H | 1.33570 | 1.86210 | -0.03290 |
| O | 3.93220 | -0.99620 | 0.01730 | H | 1.48390 | -1.19490 | 0.00820 |
| O | 3.72550 | 1.23140 | -0.01150 | H | 5.76280 | -1.84480 | 0.04060 |
| C | 5.35540 | -0.83880 | 0.02830 | H | 5.68110 | -0.30790 | -0.86480 |
| O | -0.73270 | -1.50610 | -0.04670 | H | 5.66610 | -0.29340 | 0.91800 |
| H | -0.58880 | 2.99490 | -0.00070 | H | -1.38760 | -2.21960 | -0.07810 |
| H | -3.05370 | 3.17940 | 0.02680 | | | | |

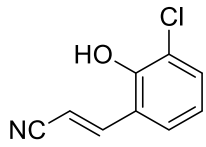


**1c**$_{truncated}$

UM06-2X/def2-TZVP Energy in solution = -936.716388

UM06-2X/def2-TZVP Zero-point energy corrected in solution = -936.588131

Number of Imaginary Frequencies = 0

SMD-M06-2X/def2-TZVP Geometry

| | | | | | | | |
|---|---|---|---|---|---|---|---|
| C | -0.08940 | 2.04910 | 0.00000 | C | 4.01070 | -0.45330 | 0.00000 |
| C | -1.44630 | 2.31130 | 0.00000 | N | 5.16140 | -0.40330 | 0.00000 |
| C | -2.34600 | 1.25530 | 0.00000 | O | -0.03120 | -1.58760 | -0.00000 |
| C | -1.86940 | -0.04280 | -0.00000 | H | 0.61980 | 2.86750 | 0.00010 |
| C | -0.50350 | -0.32480 | -0.00000 | H | -1.80930 | 3.32950 | 0.00000 |
| C | 0.41080 | 0.74240 | 0.00000 | H | -3.41350 | 1.43060 | -0.00000 |
| Cl | -2.98700 | -1.37210 | -0.00010 | H | 2.39990 | 1.52410 | 0.00010 |
| C | 1.85930 | 0.58300 | 0.00000 | H | 2.17640 | -1.53940 | -0.00000 |
| C | 2.58800 | -0.54270 | 0.00000 | H | -0.75950 | -2.22690 | -0.00000 |

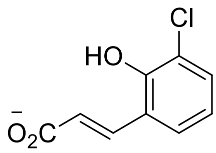


**2**$_{truncated}$

UM06-2X/def2-TZVP Energy in solution = -1032.606256

UM06-2X/def2-TZVP Zero-point energy corrected in solution = -1032.474537

Number of Imaginary Frequencies = 0

SMD-M06-2X/def2-TZVP Geometry

| | | | | | | | |
|---|---|---|---|---|---|---|---|
| C | -0.55230 | 2.06960 | -0.00000 | O | 4.27430 | 0.84360 | -0.00000 |
| C | -1.91790 | 2.29280 | -0.00000 | O | 4.37160 | -1.38660 | 0.00000 |
| C | -2.78640 | 1.21230 | -0.00000 | O | -0.38720 | -1.56360 | 0.00000 |
| C | -2.26550 | -0.06900 | 0.00000 | H | 0.13010 | 2.91070 | -0.00000 |
| C | -0.89150 | -0.30930 | 0.00000 | H | -2.30930 | 3.30070 | -0.00000 |
| C | -0.00630 | 0.78210 | -0.00000 | H | -3.85900 | 1.35270 | 0.00000 |
| Cl | -3.34370 | -1.43360 | 0.00000 | H | 1.95680 | 1.63240 | -0.00000 |
| C | 1.45430 | 0.66920 | -0.00000 | H | 1.85390 | -1.41630 | 0.00000 |
| C | 2.24060 | -0.40930 | 0.00000 | H | -1.10280 | -2.21640 | 0.00000 |
| C | 3.74220 | -0.29510 | -0.00000 | | | | |

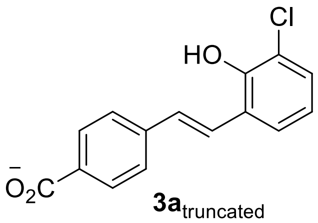

**3a**$_{truncated}$

UM06-2X/def2-TZVP Energy in solution = -1263.655461

UM06-2X/def2-TZVP Zero-point energy corrected in solution = -1263.442107

Number of Imaginary Frequencies = 0

SMD-M06-2X/def2-TZVP Geometry

| | | | | | | | |
|---|---|---|---|---|---|---|---|
| C | 2.54130 | 1.79570 | 0.35430 | C | -0.29520 | 0.86960 | -0.03840 |
| C | 3.87580 | 2.16370 | 0.32530 | C | -1.71250 | 0.49370 | -0.01510 |
| C | 4.85520 | 1.20190 | 0.12550 | C | -2.66350 | 1.45310 | -0.37410 |
| C | 4.47690 | -0.11850 | -0.03840 | C | -4.01670 | 1.15290 | -0.37900 |
| C | 3.13790 | -0.50260 | -0.01470 | C | -4.46120 | -0.11480 | -0.01940 |
| C | 2.14410 | 0.46950 | 0.17650 | C | -3.51730 | -1.07510 | 0.34590 |
| Cl | 5.69110 | -1.33960 | -0.28220 | C | -2.16610 | -0.77980 | 0.34930 |
| C | 0.73790 | 0.05310 | 0.19300 | C | -5.94290 | -0.44410 | -0.02300 |

| | | | | | | | |
|---|---|---|---|---|---|---|---|
| O | -6.28470 | -1.58250 | 0.38110 | H | -0.10700 | 1.90990 | -0.28750 |
| O | -6.73040 | 0.44460 | -0.43150 | H | -2.33000 | 2.44540 | -0.65440 |
| O | 2.74630 | -1.79160 | -0.18500 | H | -4.73780 | 1.90800 | -0.66220 |
| H | 1.78600 | 2.54890 | 0.53680 | H | -3.85180 | -2.06370 | 0.63100 |
| H | 4.15990 | 3.19720 | 0.46890 | H | -1.46030 | -1.54690 | 0.64150 |
| H | 5.90420 | 1.46480 | 0.10370 | H | 3.51600 | -2.36740 | -0.29750 |
| H | 0.55930 | -0.99610 | 0.39510 | | | | |

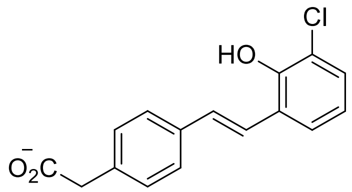


**3b**$_{truncated}$

UM06-2X/def2-TZVP Energy in solution = -1302.961267

UM06-2X/def2-TZVP Zero-point energy corrected in solution = -1302.720148

Number of Imaginary Frequencies = 0

SMD-M06-2X/def2-TZVP Geometry

| | | | | | | | |
|---|---|---|---|---|---|---|---|
| C | -2.91510 | 1.78840 | -0.32650 | C | 6.28820 | -0.29790 | -0.59380 |
| C | -4.24740 | 2.15520 | -0.41560 | O | 7.43830 | -0.80460 | -0.61790 |
| C | -5.23990 | 1.19140 | -0.31440 | O | 5.76550 | 0.35780 | -1.52550 |
| C | -4.87570 | -0.12930 | -0.12290 | O | -3.16320 | -1.80210 | 0.17180 |
| C | -3.53950 | -0.51210 | -0.02820 | H | -2.14820 | 2.54420 | -0.43450 |
| C | -2.53280 | 0.46110 | -0.12450 | H | -4.51830 | 3.18970 | -0.57700 |
| Cl | -6.10590 | -1.35160 | 0.01040 | H | -6.28690 | 1.45310 | -0.38620 |
| C | -1.13070 | 0.04220 | -0.02410 | H | -0.94140 | -1.00930 | -0.20320 |
| C | -0.11420 | 0.85540 | 0.28170 | H | -0.31570 | 1.89880 | 0.50710 |
| C | 1.29880 | 0.47360 | 0.37500 | H | 1.87270 | 2.41560 | 1.07860 |
| C | 2.22210 | 1.42250 | 0.82020 | H | 4.26510 | 1.86600 | 1.29160 |
| C | 3.56920 | 1.11240 | 0.94080 | H | 3.46980 | -2.09740 | -0.09260 |
| C | 4.04010 | -0.15650 | 0.61990 | H | 1.09530 | -1.56160 | -0.31470 |
| C | 3.12020 | -1.10460 | 0.16760 | H | 5.63540 | -1.52930 | 1.02350 |
| C | 1.77640 | -0.80040 | 0.04460 | H | 5.98730 | 0.13480 | 1.45820 |
| C | 5.49660 | -0.49370 | 0.71060 | H | -3.93680 | -2.38330 | 0.18210 |

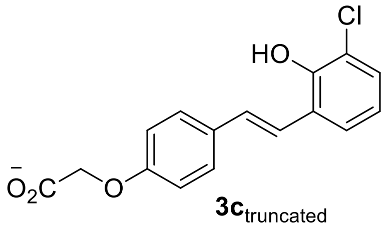

$\mathbf{3c}_{truncated}$

UM06-2X/def2-TZVP Energy in solution = -1378.183493

UM06-2X/def2-TZVP Zero-point energy corrected in solution = -1377.936739

Number of Imaginary Frequencies = 0

SMD-M06-2X/def2-TZVP Geometry

| | | | |
|---|---|---|---|
| C | 3.09230 | 1.83490 | 0.14670 |
| C | 4.37970 | 2.34490 | 0.11780 |
| C | 5.46530 | 1.48380 | 0.05570 |
| C | 5.23760 | 0.11970 | 0.02220 |
| C | 3.94810 | -0.40680 | 0.04290 |
| C | 2.84690 | 0.46130 | 0.10150 |
| Cl | 6.58450 | -0.97830 | -0.05100 |
| C | 1.49510 | -0.10760 | 0.12760 |
| C | 0.38640 | 0.55860 | -0.21320 |
| C | -0.98230 | 0.03840 | -0.18120 |
| C | -2.02120 | 0.84430 | -0.64440 |
| C | -3.34310 | 0.41620 | -0.64710 |
| C | -3.64380 | -0.85650 | -0.17560 |
| C | -2.61900 | -1.67890 | 0.29910 |
| C | -1.31340 | -1.23750 | 0.29690 |
| O | -4.89720 | -1.39050 | -0.14110 |
| C | -5.96980 | -0.59210 | -0.60450 |
| C | -6.33670 | 0.57500 | 0.31870 |
| O | -7.15630 | 1.39100 | -0.16800 |
| O | -5.83540 | 0.61360 | 1.46240 |
| O | 3.70660 | -1.74310 | -0.00760 |
| H | 2.25400 | 2.51510 | 0.22380 |
| H | 4.54380 | 3.41320 | 0.15630 |
| H | 6.47980 | 1.85830 | 0.03920 |
| H | 1.42650 | -1.14370 | 0.43690 |
| H | 0.46970 | 1.58220 | -0.56690 |
| H | -1.79220 | 1.83800 | -1.01200 |
| H | -4.11430 | 1.08260 | -1.00830 |
| H | -2.87220 | -2.66440 | 0.66930 |
| H | -0.54180 | -1.89500 | 0.67690 |
| H | -6.83300 | -1.25610 | -0.65380 |
| H | -5.77140 | -0.21720 | -1.61040 |
| H | 4.53920 | -2.23600 | -0.02990 |

**Truncated Phenoxy Radical Luminophores**

**1a**

UM06-2X/def2-TZVP Energy in solution = -766.419404

UM06-2X/def2-TZVP Zero-point energy corrected in solution = -766.336486

Number of Imaginary Frequencies = 0

SMD-M06-2X/def2-TZVP Geometry

| | | | | | | | |
|---|---|---|---|---|---|---|---|
| C | 2.33310 | -0.07880 | 0.00000 | O | -0.54970 | 2.04600 | -0.00000 |
| C | 1.70150 | -1.33790 | 0.00000 | Cl | -2.16910 | -0.41460 | -0.00000 |
| C | 0.30820 | -1.45120 | 0.00000 | H | 3.41350 | -0.02700 | 0.00000 |
| C | -0.45880 | -0.31750 | -0.00000 | H | 2.30050 | -2.23840 | 0.00000 |
| C | 0.14140 | 1.01260 | 0.00000 | H | -0.15950 | -2.42630 | 0.00000 |
| C | 1.58760 | 1.06070 | 0.00000 | H | 2.03980 | 2.04410 | 0.00000 |

**1b**

UM06-2X/def2-TZVP Energy in solution = -1071.707234

UM06-2X/def2-TZVP Zero-point energy corrected in solution = -1071.547298

Number of Imaginary Frequencies = 0

SMD-M06-2X/def2-TZVP Geometry

| | | | | | | | |
|---|---|---|---|---|---|---|---|
| C | -0.79910 | 1.85510 | -0.03480 | O | 3.51670 | -0.94430 | -0.04280 |
| C | -2.14420 | 2.21310 | -0.03540 | O | 4.38660 | 1.11200 | 0.08150 |
| C | -3.16490 | 1.24540 | -0.01890 | C | 4.84530 | -1.47960 | -0.04940 |
| C | -2.83320 | -0.07450 | -0.00020 | H | -0.05280 | 2.63790 | -0.05540 |
| C | -1.44100 | -0.51970 | 0.00420 | H | -2.41820 | 3.25900 | -0.05170 |
| C | -0.41530 | 0.52980 | -0.01560 | H | -4.20270 | 1.55000 | -0.02140 |
| O | -1.14740 | -1.72120 | 0.02650 | H | 1.11640 | -0.98500 | -0.06340 |
| Cl | -4.04490 | -1.28680 | 0.02080 | H | 1.96590 | 1.96680 | 0.09420 |
| C | 0.96730 | 0.08610 | -0.01800 | H | 5.38950 | -1.11880 | -0.92060 |
| C | 2.03590 | 0.88860 | 0.03630 | H | 5.36940 | -1.19310 | 0.86090 |
| C | 3.41920 | 0.37990 | 0.02830 | H | 4.72770 | -2.55750 | -0.09540 |

**1c**

UM06-2X/def2-TZVP Energy in solution = -936.069000

UM06-2X/def2-TZVP Zero-point energy corrected in solution = -935.953641

Number of Imaginary Frequencies = 0

SMD-water-UM06-2X/def2-TZVP Geometry

| | | | |
|---|---|---|---|
| C | 0.26580 | 1.70450 | -0.04290 |
| C | -1.02540 | 2.22690 | -0.01650 |
| C | -2.15790 | 1.39430 | 0.01060 |
| C | -1.99310 | 0.04270 | 0.00580 |
| C | -0.66750 | -0.57220 | -0.02530 |
| C | 0.47910 | 0.34230 | -0.04150 |
| O | -0.52500 | -1.80020 | -0.03270 |
| Cl | -3.34330 | -1.01200 | 0.03460 |
| C | 1.79390 | -0.26920 | -0.06550 |
| C | 2.94660 | 0.39750 | 0.08440 |
| C | 4.19020 | -0.29440 | 0.03650 |
| N | 5.20450 | -0.83800 | 0.00230 |
| H | 1.10350 | 2.38880 | -0.07430 |
| H | -1.16690 | 3.29900 | -0.01990 |
| H | -3.14910 | 1.82620 | 0.03200 |
| H | 1.82070 | -1.34260 | -0.20530 |
| H | 3.00660 | 1.46510 | 0.25310 |

**2**

UM06-2X/def2-TZVP Energy in solution = -1031.961741

UM06-2X/def2-TZVP Zero-point energy corrected in solution = -1031.843091

Number of Imaginary Frequencies = 0

SMD-M06-2X/def2-TZVP Geometry

| | | | |
|---|---|---|---|
| C | -0.15650 | 1.73370 | -0.10060 |
| C | -1.44270 | 2.24940 | -0.06590 |
| C | -2.57490 | 1.41060 | -0.00450 |
| C | -2.40390 | 0.06280 | 0.01480 |
| C | -1.07760 | -0.54780 | -0.02210 |
| C | 0.06850 | 0.36670 | -0.07410 |
| O | -0.93280 | -1.77790 | -0.00320 |
| Cl | -3.75350 | -0.99810 | 0.08310 |
| C | 1.38910 | -0.23310 | -0.09650 |
| C | 2.54000 | 0.43380 | 0.04150 |
| C | 3.88240 | -0.24990 | 0.03030 |
| O | 3.92900 | -1.48380 | -0.19180 |
| O | 4.87270 | 0.49120 | 0.25660 |
| H | 0.67990 | 2.41740 | -0.15680 |
| H | -1.59110 | 3.32050 | -0.08880 |
| H | -3.56730 | 1.83950 | 0.02340 |
| H | 1.42700 | -1.30930 | -0.21430 |
| H | 2.56420 | 1.50640 | 0.19320 |

**3a**

UM06-2X/def2-TZVP Energy in solution = -1263.011158

UM06-2X/def2-TZVP Zero-point energy corrected in solution = -1262.810834

Number of Imaginary Frequencies = 0

SMD-M06-2X/def2-TZVP Geometry

| | | | | | | | |
|---|---|---|---|---|---|---|---|
| C | 2.82260 | 2.09360 | 0.11570 | C | -3.65780 | 1.08400 | -0.16580 |
| C | 4.19350 | 2.18220 | 0.18450 | C | -2.27600 | 1.08410 | -0.16340 |
| C | 5.00350 | 1.02270 | 0.13640 | C | -5.89590 | -0.09030 | 0.00380 |
| C | 4.41890 | -0.19370 | 0.00990 | O | -6.47920 | -1.18340 | 0.20100 |
| C | 2.96910 | -0.36420 | -0.09110 | O | -6.46460 | 1.01050 | -0.19440 |
| C | 2.16350 | 0.86320 | 0.00140 | H | 2.22420 | 2.99480 | 0.16290 |
| O | 2.47400 | -1.48480 | -0.26380 | H | 4.67080 | 3.14780 | 0.28270 |
| Cl | 5.37450 | -1.62460 | -0.06210 | H | 6.07990 | 1.11020 | 0.19700 |
| C | 0.72970 | 0.86930 | -0.02490 | H | 0.30290 | 1.86670 | -0.05690 |
| C | -0.10960 | -0.19160 | 0.02640 | H | 0.29660 | -1.19000 | 0.10490 |
| C | -1.56390 | -0.11110 | 0.01100 | H | -1.75280 | -2.22810 | 0.30620 |
| C | -2.29080 | -1.29660 | 0.17590 | H | -4.21970 | -2.21600 | 0.30810 |
| C | -3.67530 | -1.29080 | 0.17660 | H | -4.19450 | 2.01290 | -0.30340 |
| C | -4.37580 | -0.10040 | 0.00590 | H | -1.75000 | 2.01910 | -0.30340 |

**3b**

UM06-2X/def2-TZVP Energy in solution = -1302.319646

UM06-2X/def2-TZVP Zero-point energy corrected in solution = -1302.090541

Number of Imaginary Frequencies = 0

SMD-M06-2X/def2-TZVP Geometry

| | | | | | | | |
|---|---|---|---|---|---|---|---|
| C | -2.90970 | 1.80660 | 0.20950 | O | -3.18240 | -1.77760 | -0.21550 |
| C | -4.23420 | 2.11270 | 0.37960 | Cl | -6.07710 | -1.43010 | 0.12090 |
| C | -5.23510 | 1.10590 | 0.35510 | C | -1.12640 | 0.09220 | -0.17440 |
| C | -4.88320 | -0.18650 | 0.15800 | C | -0.07040 | 0.94270 | -0.18850 |
| C | -3.49440 | -0.59130 | -0.03280 | C | 1.32100 | 0.56930 | -0.35630 |
| C | -2.48880 | 0.48230 | 0.00280 | C | 2.28520 | 1.58610 | -0.37470 |

| | | | | | | | |
|---|---|---|---|---|---|---|---|
| C | 3.62830 | 1.29370 | -0.53240 | H | -6.27380 | 1.37530 | 0.49290 |
| C | 4.05470 | -0.02590 | -0.67560 | H | -0.95830 | -0.96890 | -0.30570 |
| C | 3.09680 | -1.04410 | -0.65500 | H | -0.24010 | 2.00730 | -0.06610 |
| C | 1.75480 | -0.75750 | -0.50080 | H | 1.96410 | 2.61540 | -0.26640 |
| C | 5.51760 | -0.35190 | -0.78520 | H | 4.35850 | 2.09420 | -0.54700 |
| C | 6.09230 | -0.50880 | 0.63290 | H | 3.41790 | -2.07320 | -0.76510 |
| O | 6.57240 | 0.51670 | 1.17380 | H | 1.04010 | -1.57030 | -0.49270 |
| O | 6.00640 | -1.64300 | 1.16310 | H | 6.04360 | 0.45310 | -1.29610 |
| H | -2.18010 | 2.60410 | 0.23900 | H | 5.65280 | -1.28500 | -1.33010 |
| H | -4.53690 | 3.13880 | 0.53830 | | | | |

**3c**

UM06-2X/def2-TZVP Energy in solution = -1377.543277

UM06-2X/def2-TZVP Zero-point energy corrected in solution = -1377.309108

Number of Imaginary Frequencies = 0

SMD-M06-2X/def2-TZVP Geometry

| | | | |
|---|---|---|---|
| C | 3.13370 | 1.81450 | -0.23650 |
| C | 4.41950 | 2.27090 | -0.15780 |
| C | 5.50720 | 1.37910 | 0.04810 |
| C | 5.27620 | 0.05040 | 0.16250 |
| C | 3.93490 | -0.51450 | 0.08140 |
| C | 2.83570 | 0.44180 | -0.11910 |
| O | 3.73670 | -1.73560 | 0.17940 |
| Cl | 6.57770 | -1.05590 | 0.41240 |
| C | 1.52040 | -0.09600 | -0.17680 |
| C | 0.38390 | 0.63950 | -0.32040 |
| C | -0.96220 | 0.12390 | -0.36980 |
| C | -2.02280 | 1.03250 | -0.47870 |
| C | -3.34210 | 0.61850 | -0.52000 |
| C | -3.62650 | -0.74510 | -0.45510 |
| C | -2.58170 | -1.67310 | -0.35140 |
| C | -1.27650 | -1.24730 | -0.30800 |
| O | -4.87330 | -1.26820 | -0.49680 |
| C | -5.97660 | -0.38140 | -0.57750 |
| C | -6.25060 | 0.40250 | 0.71110 |
| O | -7.13100 | 1.28970 | 0.60560 |
| O | -5.61890 | 0.09550 | 1.74370 |
| H | 2.33270 | 2.52410 | -0.39200 |
| H | 4.62780 | 3.32830 | -0.25040 |
| H | 6.51580 | 1.76560 | 0.11150 |
| H | 1.45050 | -1.17240 | -0.08560 |
| H | 0.45740 | 1.71920 | -0.39900 |
| H | -1.80120 | 2.09200 | -0.52570 |
| H | -4.12740 | 1.35680 | -0.59560 |
| H | -2.82730 | -2.72610 | -0.30480 |
| H | -0.48970 | -1.98550 | -0.22710 |
| H | -6.84390 | -1.01040 | -0.77580 |
| H | -5.85940 | 0.31020 | -1.41350 |

**Population of atomic spin densities**

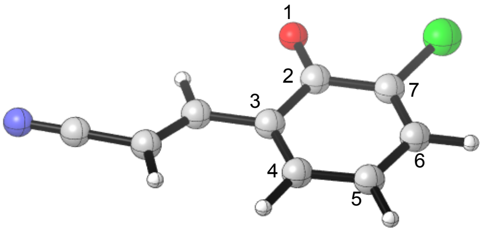


**Figure S4.** Atomic numbering of the model phenoxy radicals used in the population analyses. Probe 1c is used as an example, the computed structure is illustrated with CYLView.[12]

**Table S3.** Complete population analyses of the phenoxy radicals.

**1a**

| Atom Number | NBO Atomic Spin | Mulliken Atomic Spin |
|---|---|---|
| 1 | 0.34 | 0.34 |
| 2 | -0.01 | -0.05 |
| 3 | 0.23 | 0.27 |
| 4 | -0.1 | -0.16 |
| 5 | 0.38 | 0.45 |
| 6 | -0.11 | -0.17 |
| 7 | 0.26 | 0.30 |

**1b**

| Atom Number | NBO Atomic Spin | Mulliken Atomic Spin |
|---|---|---|
| 1 | 0.31 | 0.32 |
| 2 | -0.01 | -0.06 |
| 3 | 0.25 | 0.31 |
| 4 | -0.11 | -0.17 |
| 5 | 0.34 | 0.42 |
| 6 | -0.11 | -0.17 |
| 7 | 0.22 | 0.26 |

**1c**

| Atom Number | NBO Atomic Spin | Mulliken Atomic Spin |
|---|---|---|
| 1 | 0.31 | 0.32 |
| 2 | -0.01 | -0.07 |
| 3 | 0.24 | 0.30 |
| 4 | -0.11 | -0.17 |
| 5 | 0.35 | 0.42 |
| 6 | -0.11 | -0.17 |
| 7 | 0.22 | 0.27 |

**2**

| Atom Number | NBO Atomic Spin | Mulliken Atomic Spin |
|---|---|---|
| 1 | 0.31 | 0.32 |
| 2 | 0 | -0.05 |
| 3 | 0.27 | 0.33 |
| 4 | -0.12 | -0.19 |
| 5 | 0.32 | 0.39 |
| 6 | -0.09 | -0.14 |
| 7 | 0.19 | 0.23 |

**3a**

| Atom Number | NBO Atomic Spin | Mulliken Atomic Spin |
|---|---|---|
| 1 | 0.27 | 0.28 |
| 2 | 0.01 | -0.04 |
| 3 | 0.26 | 0.32 |
| 4 | -0.12 | -0.18 |
| 5 | 0.28 | 0.34 |
| 6 | -0.06 | -0.11 |
| 7 | 0.15 | 0.19 |

**3b**

| Atom Number | NBO Atomic Spin | Mulliken Atomic Spin |
|---|---|---|
| 1 | 0.27 | 0.28 |
| 2 | 0.02 | -0.02 |
| 3 | 0.27 | 0.32 |
| 4 | -0.11 | -0.18 |
| 5 | 0.23 | 0.29 |
| 6 | -0.04 | -0.08 |
| 7 | 0.12 | 0.15 |

**3c**

| Atom Number | NBO Atomic Spin | Mulliken Atomic Spin |
|---|---|---|
| 1 | 0.26 | 0.26 |
| 2 | 0.02 | -0.01 |
| 3 | 0.25 | 0.30 |
| 4 | -0.10 | -0.16 |
| 5 | 0.20 | 0.26 |
| 6 | -0.03 | -0.06 |
| 7 | 0.10 | 0.12 |

## Synthetic schemes and experimental procedures

### General methods

All reactions requiring anhydrous conditions were performed under an argon atmosphere. All reactions were carried out at room temperature unless stated otherwise. Chemicals and solvents were either A.R. grade or purified by standard techniques. Thin layer chromatography (TLC): silica gel plates Merck 60 F254: compounds were visualized by irradiation with UV light. Flash chromatography (FC): silica gel Merck 60 (particle size 0.040-0.063 mm), eluent given in parentheses. Reverse-phase high pressure liquid chromatography (RP-HPLC): C18 5u, 250x4.6mm, eluent given in parentheses. Preparative RP-HPLC: C18 5u, 250x21mm, eluent given in parentheses. $^{1}$H-NMR spectra were recorded using Bruker Avance operated at 400MHz. $^{13}$C-NMR spectra were recorded using Bruker Avance operated at 100 MHz. Chemical shifts were reported in ppm on the δ scale relative to a residual solvent ($CDCl_3$: δ = 7.26 for $^{1}$H-NMR and 77.16 for $^{13}$C-NMR, DMSO-d6: δ = 2.50 for $^{1}$H-NMR and 39.52 for $^{13}$C-NMR, $CD_3OD$: δ = 3.31 for $^{1}$H-NMR and 49.00 for $^{13}$C-NMR). Mass spectra were measured on Waters Xevo TQD. Absorbance, fluorescence and chemiluminescence were recorded on Molecular Devices Spectramax i3x. All reagents, including salts and solvents, were purchased from Sigma-Aldrich.

### Abbreviations

**MeCN**- acetonitrile, **DCM** - dichloromethane, **DMF** - N,N'- dimethylformamide, **EtOAc** - ethylacetate, **Hex** - hexanes, **MeOH** - methanol, **TFA** - trifluoroacetic acid, **THF** – tetrahydrofuran, **TEMP** - 2,2,6,6-Tetramethylpiperidine, **DMBA** - 1,3-dimethylbarbituric acid, **DBU** -1,8-Diazabicyclo(5.4.0)undec-7-ene, **DCC** - *N*,*N*'-Dicyclohexylcarbodiimide, **NHS** - *N*-hydroxysuccinimide, **PTSA** - *p*-Toluenesulfonic acid monohydrate.

**Synthesis of Benzyl Bromides 10a-c:**

**Compound 10a**

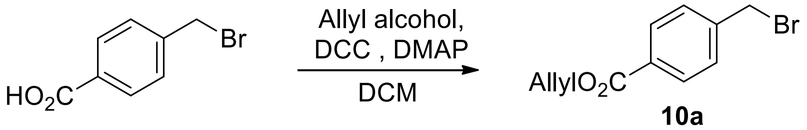

**10a**

Allyl alcohol (3.18 ml, 46.73 mmol) and 4-(Bromomethyl)benzoic acid (2.0 g, 9.34 mmol) were dissolved in DCM (15 mL). Then, DCC (1.93 g, 9.34 mmol) and DMAP (114 mg, 0.93 mmol) were added. The reaction was stirred at room temperature and monitored by TLC (Hex:EtOAc 90:10). Upon completion, the solution was filtered and the filtrate was diluted with DCM (50 mL), washed with 1M HCl (50 mL) and brine (50 ml). The organic layer was dried over $Na_2SO_4$ and evaporated under reduced pressure. Purification by column chromatography (Hex:EtOAc 90:10) afforded compound **10a** as a colorless oil (2.23 g, 94% yield). $^1$H-NMR (400 MHz, $CDCl_3$) δ 8.02 (d, J = 8.3 Hz, 2H), 7.45 (d, J = 8.3 Hz, 2H), 6.09 – 5.93 (m, 1H), 5.40 (dd, J = 17.2, 1.5 Hz, 1H), 5.28 (dd, J = 10.5, 1.2 Hz, 1H), 4.81 (dt, J = 5.5, 1.3 Hz, 2H), 4.49 (s, 2H). $^{13}$C-NMR (126 MHz, $CDCl_3$) δ 165.82, 142.87, 140.00, 132.25, 130.28, 129.19, 118.51, 65.82, 55.89.

**Compound 10b**

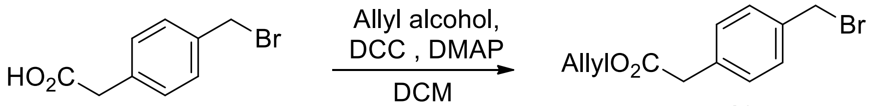

**10b**

Allyl alcohol (2.98 ml, 43.86 mmol) and 4-(Bromomethyl)phenylacetic acid (2 g, 8.77 mmol) were dissolved in DCM (15 mL). Then, DCC (1.81 g, 8.77 mmol) and DMAP (108 mg, 0.88 mmol) were added. The reaction was stirred at room temperature and monitored by TLC (Hex:EtOAc 90:10). Upon completion, the solution was filtered and the filtrate was diluted with DCM (50 mL), washed with 1M HCl (50 mL) and brine (50 ml). The organic layer was dried over $Na_2SO_4$ and evaporated under reduced pressure. Purification by column chromatography (Hex:EtOAc 90:10) afforded compound **10b** as a colorless oil (2.16 g, 92% yield). $^1$H-NMR (400 MHz, $CDCl_3$) δ 7.36 (d, J = 8.2 Hz, 2H), 7.27 (d, J = 8.2 Hz, 2H), 5.91 (ddt, J = 17.2, 10.4, 5.7 Hz, 1H), 5.28 (dd, J = 17.2, 1.5 Hz, 1H), 5.23 (dd, J = 10.4, 1.3 Hz, 1H), 4.60 (dt, J = 5.7, 1.4 Hz, 2H), 4.48 (s, 2H), 3.65 (s, 2H). $^{13}$C-NMR (400 MHz, $CDCl_3$) δ 170.97, 136.76, 134.29, 132.02, 129.82, 129.37, 118.47, 65.64, 41.03, 33.27.

**Compound 10c**

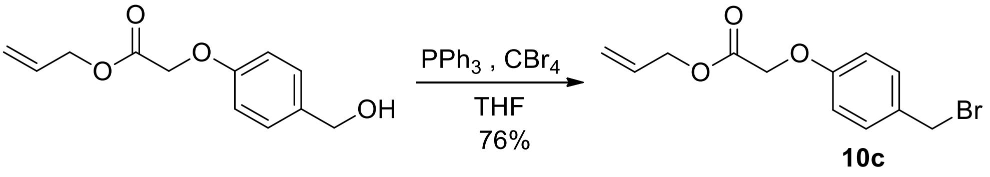

Triphenylphosphine (2.60 gr, 9.90 mmol) was added to a solution of allyl 2-(4-(hydroxymethyl)phenoxy)acetate[13] (2 g, 9.00 mmol) and $CBr_4$ (3.28 g, 9.90 mmol) in THF (25 mL). The reaction was stirred at room temperature and monitored by TLC (Hex:EtOAc 90:10). Upon completion, EtOAc (100 mL) was added, and organic phase was washed with brine (100 ml) and dried over $Na_2SO_4$. Purification by column chromatography (Hex:EtOAc 90:10) afforded compound **10c** as a colorless oil (1.94 g, 76% yield). $^1$H-NMR (400 MHz, $CDCl_3$) δ 7.32 (d, $J$ = 8.6 Hz, 2H), 6.87 (d, $J$ = 8.7 Hz, 2H), 5.92 (ddt, $J$ = 16.4, 10.5, 5.8 Hz, 1H), 5.33 (dd, $J$ = 17.2, 1.4 Hz, 1H), 5.27 (dd, $J$ = 10.4, 1.1 Hz, 1H), 4.70 (dd, $J$ = 5.8, 1.1 Hz, 2H), 4.65 (s, 2H), 4.48 (s, 2H). $^{13}$C-NMR (400 MHz, $CDCl_3$) δ 168.55, 157.94, 131.50, 131.29, 130.71, 119.41, 115.08, 66.10, 65.45, 33.71.

**Synthesis of Phosphonium Salts 5a-c:**

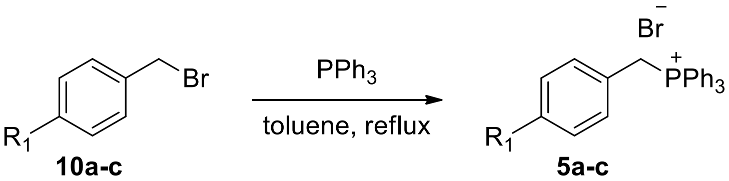

**Procedure A:**

To a solution of benzyl bromide **10** (1 mmol) in toluene (5 mL) was added triphenylphosphine (1.5 eq). Reaction was refluxed for 4 hours upon which white solid precipitated and TLC (Hex:EtOAc 80:20) indicated disappearance of starting material. The reaction mixture was cooled and the precipitate was collected and washed with toluene (20 mL) and hexane (20 ml). The product was dried under vacuum to give the benzyltriphenylphosphonium bromide **5** as a white solid.

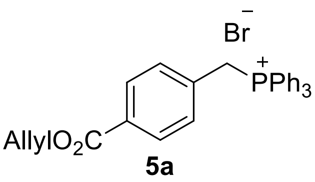

**5a** was prepared from **10a** according to **Procedure A** (97% yield). $^1$H-NMR (400 MHz, $CDCl_3$) δ 7.69 (m, 11H), 7.54 (m, 6H), 7.18 (dd, *J* = 8.4, 2.4 Hz, 2H), 5.92 (ddt, *J* = 17.1, 10.5, 5.7 Hz, 1H), 5.55 (d, *J* = 15.2 Hz, 2H), 5.30 (ddd, *J* = 17.2, 3.0, 1.5 Hz, 1H), 5.20 (ddd, *J* = 10.5, 2.5, 1.2 Hz, 1H), 4.69 (dt, *J* = 5.7, 1.3 Hz, 2H). $^{13}$C-NMR (400 MHz, $CDCl_3$) δ 165.72, 135.20, 135.17, 134.58, 134.48, 133.09, 133.00, 132.06, 131.87, 131.82, 129.98, 129.82, 129.79, 118.56, 117.99, 117.13, 65.79. MS (ES+): m/z calc. for $C_{29}H_{26}O_2P$: 437.2; found: 437.1 $[M]^+$.

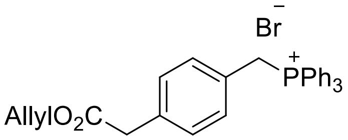


**5b**

**5b** was prepared from **10b** according to **Procedure A** (97% yield). $^1$H-NMR (400 MHz, $CDCl_3$) δ 7.79 – 7.49 (m, 15H), 7.02 – 6.93 (m, 4H), 5.81 (ddt, *J* = 17.1, 10.5, 5.7 Hz, 1H), 5.23 (d, *J* = 14.5 Hz, 2H), 5.21 – 5.16 (m, 1H), 5.14 (m, 1H), 4.50 (d, *J* = 5.7 Hz, 2H), 3.50 (d, *J* = 1.3 Hz, 2H). $^{13}$C-NMR (101 MHz, $CDCl_3$) δ 170.93, 135.09, 134.44, 134.35, 131.89, 131.73, 131.67, 130.28, 130.16, 129.83, 126.12, 126.03, 118.42, 118.10, 117.25, 65.53, 40.78. MS (ES+): m/z calc. for $C_{30}H_{28}O_2P$: 451.2; found: 451.4 $[M]^+$.

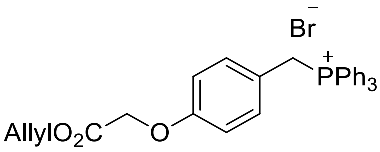


**5c**

**5c** was prepared from **10c** according to **Procedure A** (91% yield). $^1$H-NMR (400 MHz, $CDCl_3$) δ 7.72 – 7.48 (m, 15H), 6.93 (dd, *J* = 8.8, 2.5 Hz, 4H), 6.53 (d, *J* = 8.3 Hz, 2H), 5.88 – 5.73 (m, 1H), 5.21 (dd, *J* = 17.2, 1.4 Hz, 1H), 5.18 – 5.11 (m, 3H), 4.55 (dt, *J* = 5.8, 1.2 Hz, 2H), 4.47 (s, 2H). $^{13}$C-NMR (400 MHz, $CDCl_3$) δ 168.25, 157.76, 135.04, 134.42, 134.32, 132.84, 132.79, 131.29, 130.24, 130.12, 119.97, 119.89, 119.25, 118.07, 117.22, 114.89, 65.86, 65.07. MS (ES+): m/z calc. for $C_{30}H_{28}O_3P$: 467.2; found: 467.3 $[M]^+$.

**Synthesis of Benzoates 7a-f:**

Benzoates **7a** and **7b** were prepared as reported previously.[14]

**Benzoate 7c:**

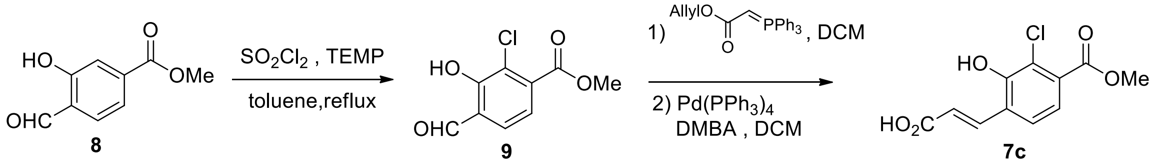


**Compound 9.** A mixture of compound **8**[15] (500 mg, 2.78 mmol) and TEMP (4.7 µL, 0.28 mmol) in toluene (10 mL) was heated to reflux. Then, $SO_2Cl_2$ (225 µL, 2.78 mmol) dissolved in toluene (3 mL) was added dropwise over 10 min. The mixture was kept in reflux for 1 hour and monitored by TLC (Hex:EtOAc 70:30). Upon completion, the reaction was cooled to room temperature and 30 mL of EtOAc were added. Organic phase was washed with brine (30 mL) and dried over $Na_2SO_4$. Purification by column chromatography (Hex:EtOAc 70:30) afforded compound **9** as a white solid (315 mg, 53% yield). $^1$H-NMR (400 MHz, $CDCl_3$) δ 11.56 (s, 1H), 9.95 (s, 1H), 7.56 (d, *J* = 8.0 Hz, 1H), 7.34 (d, *J* = 8.0 Hz, 1H), 3.96 (s, 3H). $^{13}$C-NMR (400 MHz, $CDCl_3$) δ 196.02, 165.75, 157.84, 138.10, 131.25, 122.10, 121.74, 120.76, 53.08. MS (ES-): m/z calc. for $C_9H_7ClO_4$: 214.0; found: 213.0 $[M-H]^-$.

**7c:**

Allyl (triphenylphosphoranylidene)acetate (101 mg, 0.28 mmol) was added to a solution of compound **9** (50 mg, 0.23 mmol) in DCM (2 mL). The mixture was stirred at room temperature for 15 minutes upon which time TLC (Hex:EtOAc 70:30) indicated full conversion of the starting material. Then, the solution was diluted with EtOAc (25 mL), washed with 0.5M HCl (25 mL) and brine (25 ml). The organic layer was dried over $Na_2SO_4$ and evaporated under reduced pressure. The residue was dissolved in DCM (2 ml), followed by the addition of DMBA (73 mg, 0.47 mmol) and tetrakis(triphenylphosphine)palladium(27 mg, 0.02 mmol). The reaction was stirred at room temperature and allyl deprotection was monitored by TLC (Hex:EtOAc 70:30). Upon completion, the solvent was concentrated under reduced pressure and the crude product was purified by preparative RP-HPLC to give **7c** as a white solid (37 mg, 62% yield). $^1$H-NMR (400 MHz, MeOD) δ

7.97 (d, $J$ = 16.2 Hz, 1H), 7.49 (d, $J$ = 8.2 Hz, 1H), 7.30 (d, $J$ = 8.2 Hz, 1H), 6.60 (d, $J$ = 16.2 Hz, 1H), 3.90 (s, 3H). $^{13}$C-NMR (400 MHz, MeOD) δ 169.08, 166.27, 152.54, 139.01, 131.68, 126.50, 126.04, 121.54, 121.17, 51.98. MS (ES-): m/z calc. for $C_{11}H_9ClO_5$: 256.0; found: 255.0 $[M-H]^-$.

**Benzoates 7d-f:**

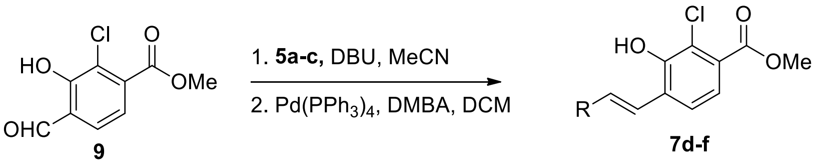


**9** **7d-f**

**Procedure B**

Aldehyde **9** (50 mg, 0.23 mmol) and phosphonium salt **5** (1.1 eq) were dissolved in MeCN (2 ml). Then, DBU (1.2 eq) was added, the solution was heated to reflux and reaction was monitored by RP-HPLC. Upon completion, the reaction mixture was cooled, diluted with EtOAc (25 mL) and washed with 0.5M HCl (25 mL) and brine (25 ml). The organic layer was dried over $Na_2SO_4$ and evaporated under reduced pressure. The residue was dissolved in DCM (2 ml), followed by the addition of DMBA (73 mg, 0.47 mmol) and tetrakis(triphenylphosphine)palladium (27 mg, 0.02 mmol). The reaction was stirred at room temperature and allyl deprotection was monitored by TLC (Hex:EtOAc 70:30). Upon completion, the solvent was concentrated under reduced pressure and the crude product was purified by preparative RP-HPLC to give the corresponding benzoate **7** as a white solid.

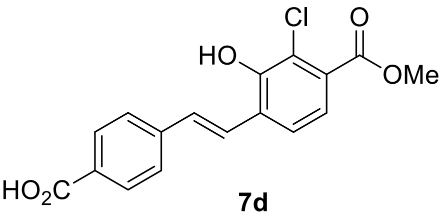


**7d**

**7d** was prepared using phosphonium salt **5a** according to **Procedure B** (79% yield). $^{1}$H-NMR (400 MHz, DMSO) δ 7.94 (d, $J$ = 8.3 Hz, 2H), 7.72 (m, $J$ = 8.3, 3.6 Hz, 3H), 7.61 (d, $J$ = 16.5 Hz, 1H), 7.42 (d, $J$ = 16.4 Hz, 1H), 7.30 (d, $J$ = 8.2 Hz, 1H), 3.83 (s, 3H). $^{13}$C-NMR (101 MHz, DMSO) δ 177.47, 167.05, 165.59, 151.11, 141.16, 130.52, 130.07, 129.85, 129.28, 126.77, 124.47, 121.45, 120.61, 52.46. MS (ES-): m/z calc. for $C_{17}H_{13}ClO_5$: 332.1; found: 331.1 $[M-H]^-$.

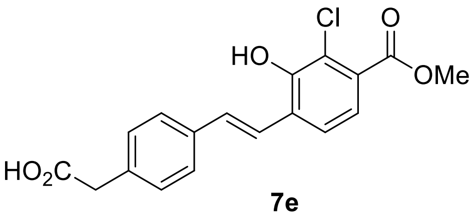

**7e**

**7e** was prepared using phosphonium salt **5b** according to **Procedure B** (66% yield). $^{1}$H-NMR (400 MHz, DMSO) δ 12.33 (s, 1H), 9.89 (s, 1H), 7.68 (d, *J* = 8.3 Hz, 1H), 7.54 (d, *J* = 7.1 Hz, 2H), 7.46 (d, *J* = 16.4 Hz, 1H), 7.33 (d, *J* = 16.7 Hz, 1H), 7.29 (m, *J* = 10.4, 3.8 Hz, 3H), 3.83 (s, 3H), 3.58 (s, 2H). $^{13}$C-NMR (400 MHz, DMSO) δ 173.15, 166.20, 151.35, 135.93, 135.70, 131.98, 130.47, 129.98, 127.28, 124.67, 122.31, 122.19, 121.23, 53.00, 41.05. MS (ES-): m/z calc. for $C_{18}H_{15}ClO_5$: 346.1; found: 345.1 [M-H]$^-$.

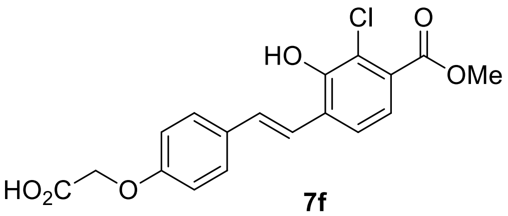

**7f**

**7f** was prepared using phosphonium salt **5c** according to **Procedure B** (75% yield). $^{1}$H-NMR (400 MHz, DMSO) δ 13.02 (s, 1H), 9.83 (s, 1H), 7.65 (d, *J* = 8.1 Hz, 1H), 7.54 (d, *J* = 8.0 Hz, 2H), 7.40 – 7.23 (m, 3H), 6.94 (d, *J* = 8.0 Hz, 2H), 4.70 (s, 2H), 3.82 (s, 3H). $^{13}$C-NMR (400 MHz, DMSO) δ 170.62, 166.20, 158.48, 131.84, 130.77, 130.66, 129.57, 128.72, 124.39, 122.26, 121.17, 120.54, 115.43, 65.11, 52.97. MS (ES-): m/z calc. for $C_{18}H_{15}ClO_6$: 362.1 found: 361.1 [M-H]$^-$.

**Synthesis of Enol-Ethers 6a-c**

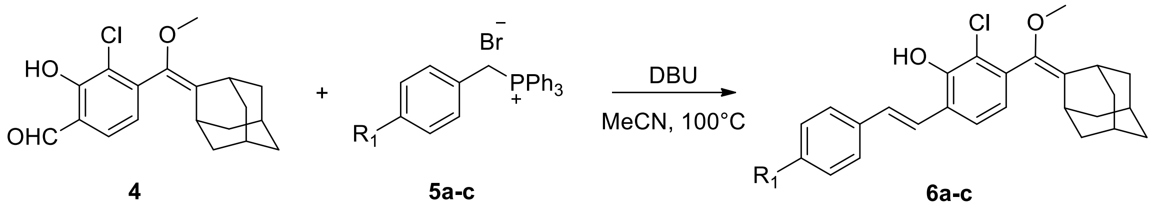

**Procedure C**

Compound **4**[16] (200 mg, 0.6 mmol) and phosphonium salt **5** (1.1 eq) were dissolved in MeCN (2 mL). Then, DBU (1.2 eq) was added, the solution was heated to reflux and reaction was monitored by RP-HPLC. Upon completion, the reaction mixture was cooled, diluted with EtOAc (25 mL) and washed with 0.5M HCl (25 mL) and brine (25 ml). The organic layer was dried over $Na_2SO_4$ and evaporated under reduced pressure. The residue was purified by column chromatography (Hex:EtOAc 85:15) to afford **6**.

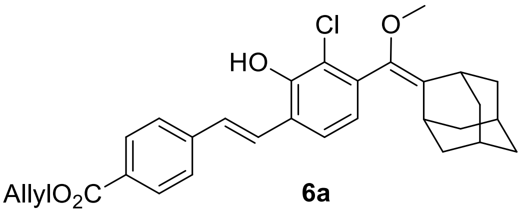

**6a**

**6a** was prepared using phosphonium salt **5a** according to **Procedure C**., and was obtained as a white solid (69% yield). $^1$H-NMR (400 MHz, $CDCl_3$) δ 8.05 (d, *J* = 8.0 Hz, 2H), 7.59 (d, *J* = 8.1 Hz, 2H), 7.52 (d, *J* = 16.4 Hz, 1H), 7.47 (d, *J* = 8.0 Hz, 1H), 7.23 (d, *J* = 16.4 Hz, 1H), 6.87 (d, *J* = 8.0 Hz, 1H), 6.16 (s, 1H), 6.11 – 5.99 (m, 1H), 5.42 (dd, *J* = 17.2, 1.2 Hz, 1H), 5.29 (dd, *J* = 10.4, 1.1 Hz, 1H), 4.83 (d, *J* = 5.6 Hz, 2H), 3.33 (s, 3H), 3.29 (s, 1H), 2.16 (s, 1H), 2.03 – 1.72 (m, 12H). $^{13}$C-NMR (400 MHz, $CDCl_3$) δ 166.09, 149.36, 142.18, 139.79, 134.40, 132.39, 132.32, 130.16, 129.38, 129.11, 126.54, 125.32, 124.66, 124.50, 123.68, 121.34, 118.23, 65.58, 57.18, 39.13, 37.17, 32.99, 29.80, 28.40. MS (ES+): m/z calc. for $C_{30}H_{31}ClO_4$: 490.2; found: 491.2 $[M+H]^+$.

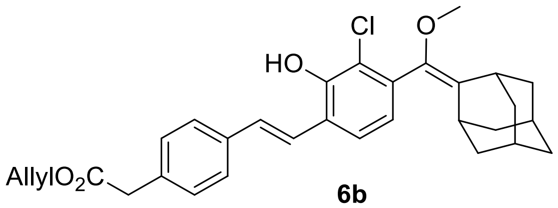

**6b**

**6b** was prepared using phosphonium salt **5b** according to **Procedure C**., and was obtained as a white solid (73% yield). $^{1}$H-NMR (400 MHz, $CDCl_3$) δ 7.50 (d, *J* = 8.2 Hz, 2H), 7.45 (d, *J* = 8.3 Hz, 1H), 7.40 (d, *J* = 16.5 Hz, 1H), 7.29 (d, *J* = 8.1 Hz, 2H), 7.17 (d, *J* = 16.6 Hz, 1H), 6.85 (d, *J* = 7.9 Hz, 1H), 5.99 – 5.84 (m, 1H), 5.33 – 5.25 (m, 1H), 5.24 – 5.19 (m, 1H), 4.61 (d, *J* = 5.7 Hz, 2H), 3.66 (s, 2H), 3.32 (s, 3H), 3.28 (s, 1H), 2.09 (s, 1H), 2.01 – 1.66 (m, 12H). $^{13}$C-NMR (400 MHz, $CDCl_3$) δ 171.17, 149.08, 139.89, 136.55, 133.69, 133.50, 130.04, 129.68, 129.23, 129.09, 126.93, 125.08, 124.34, 123.60, 122.70, 118.35, 65.58, 57.11, 41.14, 39.12, 37.19, 32.97, 29.77, 28.40. MS (ES+): m/z calc. for $C_{31}H_{33}ClO_4$: 504.2; found: 505.3 $[M+H]^+$.

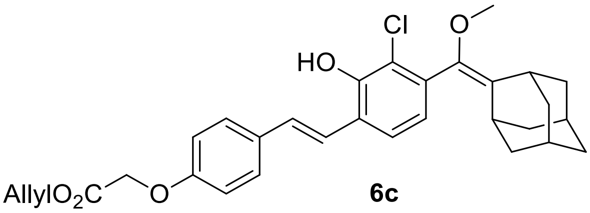

**6c**

**6c** was prepared using phosphonium salt **5c** according to **Procedure C**., and was obtained as a white solid (72% yield). $^{1}$H-NMR (400 MHz, $CDCl_3$) δ 7.47 (d, *J* = 8.8 Hz, 2H), 7.43 (d, *J* = 8.0 Hz, 1H), 7.30 (d, *J* = 16.5 Hz, 1H), 7.13 (d, *J* = 16.4 Hz, 1H), 6.90 (d, *J* = 8.8 Hz, 2H), 6.84 (d, *J* = 7.9 Hz, 1H), 6.12 (s, 1H), 5.98 – 5.88 (m, 1H), 5.34 (dd, *J* = 17.2, 1.4 Hz, 1H), 5.30 – 5.25 (m, 1H), 4.73 – 4.69 (m, 2H), 4.67 (s, 2H), 3.32 (s, 3H), 3.28 (s, 1H), 2.16 (s, 1H), 1.98 – 1.71 (m, 12H). $^{13}$C-NMR (400 MHz, $CDCl_3$) δ 168.62, 157.61, 148.90, 139.90, 133.34, 131.98, 131.48, 130.29, 129.75, 128.05, 125.29, 124.13, 123.59, 121.15, 119.24, 114.95, 114.44, 77.50, 77.19, 76.87, 65.98, 65.42, 57.12, 39.14, 38.86, 37.18, 32.95, 29.76, 28.38. MS (ES+): m/z calc. for $C_{31}H_{33}ClO_5$: 520.2; found: 521.3 $[M+H]^+$.

**Synthesis of Luminophores**

Luminophores **1a-c** were prepared as reported previously.[14]

**Luminophore 2**

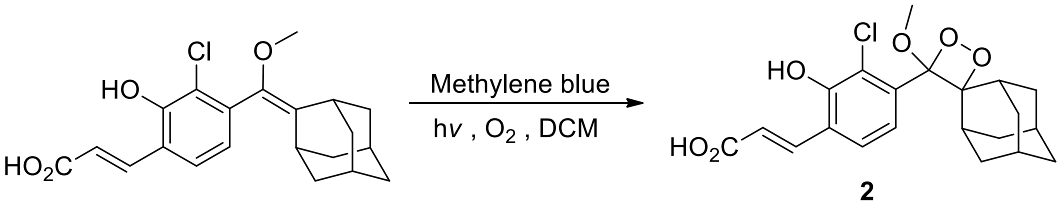


**2**

Acrylic acid substituted enol ether[17] (50 mg, 0.13 mmol) and a catalytic amount of methylene blue were dissolved in 10 mL of DCM. Then, oxygen was bubbled through the solution while irradiating with yellow light. The reaction was monitored by RP-HPLC. Upon completion (30 min), the solvent was concentrated under reduced pressure and the product was purified by preparative RP-HPLC. The product **2** was obtained as a white solid (44 mg, 81%).$^1$H-NMR (400 MHz, $CDCl_3$) δ 8.05 (d, J = 16.1 Hz, 1H), 7.70 (d, J = 7.9 Hz, 1H), 7.56 (d, J = 8.3 Hz, 1H), 6.69 (d, J = 16.1 Hz, 1H), 6.50 (bs, 1H), 3.23 (s, 3H), 3.02 (s, 1H), 2.22 (d, J = 12.0 Hz, 1H), 2.05 (s, 1H), 1.91 – 1.56 (m, 8H), 1.47 (dd, J = 12.9, 2.6 Hz, 1H), 1.36 (dd, J = 13.3, 2.7 Hz, 1H), 1.28 – 1.14 (m, 1H). $^{13}$C-NMR (101 MHz, $CDCl_3$) δ 171.67, 151.21, 140.90, 134.46, 127.29, 124.91, 123.63, 120.44, 111.69, 96.47, 77.35, 49.83, 36.66, 34.22, 33.61, 32.98, 32.30, 31.72, 26.28, 25.94 .MS (ES-): m/z calc. for $C_{21}H_{23}ClO_6$: 406.1; found: 405.3 $[M-H]^-$.

**Luminophores 3a-c**

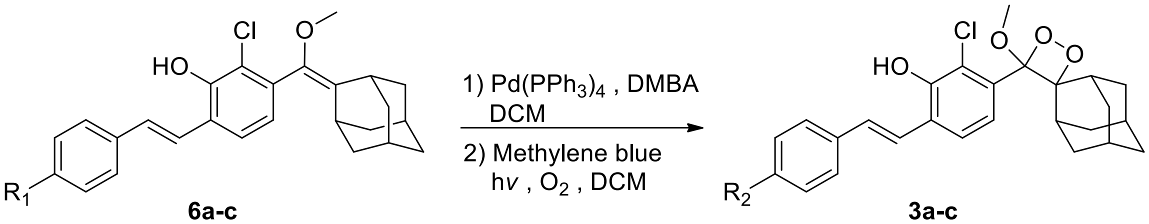


**Procedure D**

Enol-ether **6** (0.1 mmol) was dissolved in DCM, followed by the addition of DMBA (32 mg, 0.2 mmol) and tetrakis(triphenylphosphine)palladium (12 mg, 0.01 mmol). The reaction was stirred

at room temperature and monitored by TLC (Hex:EtOAc 60:40). Upon allyl deprotection, the solvent was concentrated under reduced pressure and the crude product was filtered through a short silica gel pad using EtOAc as eluent. The filtrate was concentrated under reduced pressure and the residue was dissolved in 10 mL of DCM followed by the addition of a catalytic amount of methylene blue. Then, oxygen was bubbled through the solution while irradiating with yellow light. The reaction was monitored by RP-HPLC. Upon completion (about 15 min), the solvent was concentrated under reduced pressure and the product was purified by preparative RP-HPLC to afford **3** as a white solid.

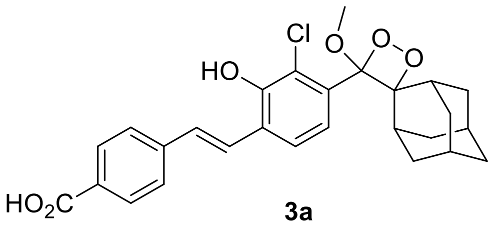


**3a**

**3a** was prepared from **6a** according to **Procedure D** (white solid, 47% yield). $^1$H-NMR (400 MHz, DMSO) δ 9.82 (s, 1H), 7.94 (d, *J* = 8.4 Hz, 2H), 7.77 (d, *J* = 8.4 Hz, 1H), 7.70 (d, *J* = 8.4 Hz, 2H), 7.60 (d, *J* = 16.4 Hz, 1H), 7.48 (d, *J* = 8.4 Hz, 1H), 7.39 (d, *J* = 16.4 Hz, 1H), 3.11 (s, 3H), 2.86 (s, 1H), 2.25 (d, *J* = 11.6 Hz, 1H), 1.98 (s, 1H), 1.77 – 1.50 (m, 8H), 1.45 (d, *J* = 12.6 Hz, 1H), 1.32 (d, *J* = 11.3 Hz, 1H), 1.23 (d, *J* = 16.2 Hz, 1H). $^{13}$C-NMR (400 MHz, DMSO) δ 167.05, 151.32, 141.36, 131.20, 129.87, 127.82, 126.69, 124.86, 124.43, 123.58, 119.95, 111.51, 95.37, 49.24, 35.96, 33.32, 33.02, 31.90, 31.76, 31.13, 30.86, 25.57, 25.27. MS (ES-): m/z calc. for $C_{27}H_{27}ClO_6$: 482.2; found: 481.2 $[M-H]^-$.

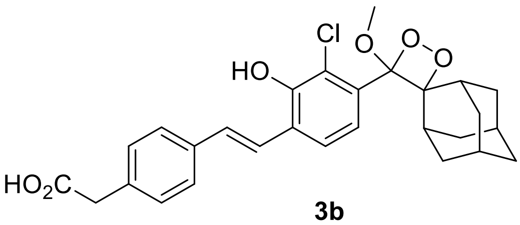


**3b**

**3b** was prepared from **6b** according to **Procedure D** (white solid, 51% yield). $^1$H-NMR (400 MHz, $CDCl_3$) δ 7.65 (d, *J* = 8.2 Hz, 1H), 7.59 (d, *J* = 8.3 Hz, 1H), 7.53 (d, *J* = 8.1 Hz, 2H), 7.41 (d, *J* = 16.5 Hz, 1H), 7.30 (d, *J* = 8.1 Hz, 2H), 7.24 (d, *J* = 16.7 Hz, 1H), 6.30 (s, 1H), 3.24 (s, 3H), 3.02 (s, 1H), 2.26 (d,

*J* = 12.2 Hz, 1H), 2.10 (s, 1H), 1.74 (m, 8H), 1.48 (dd, *J* = 12.9, 2.6 Hz, 1H), 1.36 (dd, *J* = 13.2, 2.7 Hz, 1H), 1.26 (s, 1H). $^{13}$C-NMR (400 MHz, $CDCl_3$) δ 176.71, 148.95, 135.98, 132.57, 130.64, 130.31, 129.33, 126.60, 126.44, 124.18, 124.05, 121.87, 117.61, 111.34, 95.85, 49.16, 40.25, 36.11, 33.61, 32.97, 32.32, 31.78, 31.13, 25.71, 25.37. MS (ES-): m/z calc. for $C_{28}H_{29}ClO_6$: 496.2; found: 495.2 $[M-H]^-$.

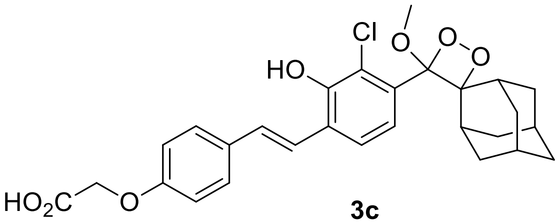


**3c**

**3c** was prepared from **6c** according to **Procedure D** (white solid, 39% yield). $^{1}$H-NMR (400 MHz, $CDCl_3$) δ 7.63 (d, *J* = 8.2 Hz, 1H), 7.57 (d, *J* = 8.3 Hz, 1H), 7.51 (d, *J* = 8.7 Hz, 2H), 7.31 (d, *J* = 16.5 Hz, 1H), 7.20 (d, *J* = 16.5 Hz, 1H), 6.93 (d, *J* = 8.7 Hz, 2H), 4.71 (s, 2H), 3.23 (s, 4H), 3.01 (s, 1H), 2.25 (d, *J* = 12.3 Hz, 1H), 2.10 (s, 1H), 1.92 – 1.59 (m, 8H), 1.48 (dd, *J* = 12.8, 2.4 Hz, 1H), 1.35 (dd, *J* = 13.2, 2.5 Hz, 1H), 1.25 (s, 1H). $^{13}$C-NMR (400 MHz, $CDCl_3$) δ 172.93, 156.87, 148.80, 131.02, 130.32, 130.00, 127.76, 126.64, 124.19, 123.84, 120.40, 114.47, 111.36, 95.87, 64.39, 49.15, 36.11, 33.61, 32.96, 32.30, 31.78, 31.13, 31.05, 25.70, 25.37. MS (ES-): m/z calc. for $C_{28}H_{29}ClO_7$: 512.2; found: 511.2 $[M-H]^-$.

**Synthesis of Compound 11**

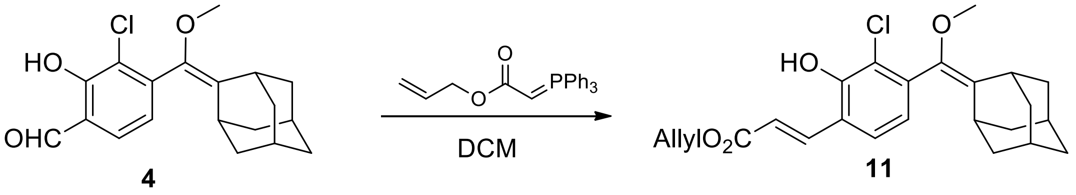


Allyl (triphenylphosphoranylidene)acetate (239 mg, 0.66 mmol) was added to a solution of compound **4**[16] (200 mg, 0.60 mmol) in DCM (5 mL). The mixture was stirred at room temperature for 15 minutes upon which time TLC (Hex:EtOAc 80:20) indicated full conversion of the starting material. Then, the solution was diluted with EtOAc (50 mL), washed with 0.5M HCl (50 mL) and brine (50 mL). The organic layer was dried over $Na_2SO_4$ and evaporated under reduced pressure. The residue was purified by column chromatography (Hex:EtOAc 85:15) affording compound **11** as a white solid (210 mg, 84% yield). $^1$H-NMR (400 MHz, $CDCl_3$) δ 7.97 (d, *J* = 16.2 Hz, 1H), 7.38 (d, *J* = 8.0 Hz, 1H), 6.86 (d, *J* = 7.9 Hz, 1H), 6.64 (d, *J* = 16.1 Hz, 1H), 6.28 (s, 1H), 6.00 (ddt, *J* = 17.1, 10.5, 5.7 Hz, 1H), 5.38 (ddd, *J* = 17.2, 3.0, 1.5 Hz, 1H), 5.27 (ddd, *J* = 10.4, 2.5, 1.2 Hz, 1H), 4.73 (d, *J* = 5.7 Hz, 2H), 3.31 (s, 3H), 3.27 (s, 1H), 2.12 (s, 1H), 1.99 – 1.62 (m, 12H). $^{13}$C-NMR (400 MHz, $CDCl_3$) δ 166.95, 150.74, 139.48, 136.71, 133.00, 132.47, 126.91, 123.76, 122.04, 121.62, 119.92, 118.34, 65.38, 57.44, 39.18, 37.16, 33.02, 29.85, 28.42. MS (ES-): m/z calc. for $C_{24}H_{27}ClO_4$: 414.2; found: 413.2 $[M-H]^-$.

**NAD(P)H Probes**

**Synthesis of Mesylate 16**

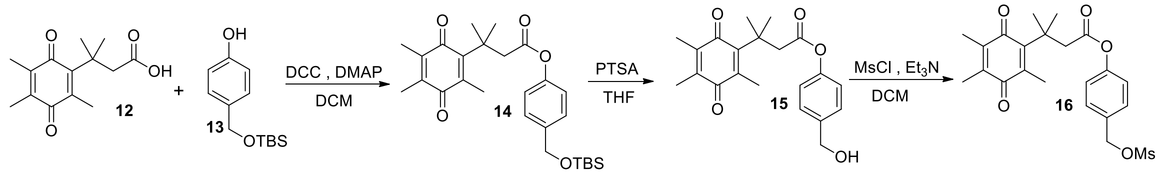


**Compound 14**

Compound **12**[18] (1.00 g, 4 mmol) and compound **13**[19] (952 mg, 4 mmol) were dissolved in DCM (15 mL). Then, DCC (906 mg, 4.4 mmol) and DMAP (50 mg, 0.44 mmol) were added. The reaction was stirred at room temperature and monitored by TLC (Hex:EtOAc 90: 10). Upon completion, the solution was filtered and the filtrate was diluted with EtOAc (100 mL), washed with 1M HCl (50 mL) and brine (50 ml). The organic layer was dried over $Na_2SO_4$ and evaporated under reduced pressure. The residue was purified by column chromatography (Hex:EtOAc 90:10) affording compound **14** as a yellow oil (1.78 mg, 95% yield). $^1$H-NMR (400 MHz, $CDCl_3$) δ 7.28 (d, *J* = 8.7 Hz, 2H), 6.92 (d, *J* = 8.6 Hz, 2H), 4.69 (s, 2H), 3.22 (s, 2H), 2.16 (s, 3H), 1.92 (m, 6H), 1.52 (s, 6H), 0.92 (s, 9H), 0.08 (s, 6H). $^{13}$C-NMR (400 MHz, $CDCl_3$) δ 190.44, 186.96, 170.94, 151.58, 148.76, 142.46, 138.60, 138.06, 126.50, 120.69, 63.88, 55.29, 47.22, 37.97, 28.45, 24.99, 24.23, 13.86, 12.16, 11.63. MS (ES+): m/z calc. for $C_{27}H_{38}O_5Si$: 470.3; found: 493.3 $[M+Na]^+$.

**Compound 15**

Compound **14** (1.00 g, 2.13 mmol) was dissolved in THF (10 mL) followed by the addition of PTSA (403 mg, 2.33 mmol). The reaction was stirred at 50°C and monitored by TLC (Hex:EtOAc 80:20). Upon completion, the reaction was diluted with EtOAc (100 mL), washed with 1M HCl (50 mL) and brine (50 ml). The organic layer was dried over $Na_2SO_4$ and evaporated under reduced pressure. The residue was purified by column chromatography (Hex:EtOAc 70:30) affording compound **15** as a yellow oil (749 mg, 99% yield). $^1$H-NMR (400 MHz, $CDCl_3$) δ 7.33 (d, *J* = 8.6 Hz, 2H), 6.96 (d, *J* = 8.5 Hz, 2H), 4.65 (s, 2H), 3.23 (s, 2H), 2.16 (s, 3H), 1.92 (m, 6H), 1.52 (s, 6H). $^{13}$C-NMR (400 MHz, $CDCl_3$) δ 190.44, 186.96, 170.93, 151.51, 149.34, 142.42, 138.67, 138.09, 127.59, 121.12, 64.22, 59.95, 47.20, 37.96, 33.41, 28.46, 25.12, 24.45, 13.89, 13.73, 12.17, 11.64. MS (ES+): m/z calc. for $C_{21}H_{24}O_5$: 356.2; found: 379.2 $[M+Na]^+$.

**Compound 16**

A solution of compound **15** (500 mg, 1.40 mmol) and $Et_3N$ (294 µL, 2.10 mmol) in DCM (10 mL) was cooled to 0°C. Then, a solution of methanesulfonyl chloride (130 µL, 0.17 mmol) in DCM (5 mL) was added in one portion. The reaction mixture was stirred for 30 minutes at room temperature and monitored by TLC (DCM). Upon completion, the mixture was concentrated under reduced pressure and the product was purified by column chromatography on silica gel (Hex:EtOAc 75:25) affording compound **16** as a yellow oil (524 mg, 86% yield). $^1$H-NMR (400 MHz, $CDCl_3$) δ 7.40 (d, *J* = 8.4 Hz, 2H), 7.02 (d, *J* = 8.5 Hz, 2H), 5.19 (s, 2H), 3.25 (s, 2H), 2.92 (s, 3H), 2.17 (s, 3H), 1.92 (s, 6H), 1.52 (s, 6H). $^{13}$C-NMR (126 MHz, DMSO) δ 190.96, 187.51, 171.26, 151.85, 151.31, 142.93, 139.48, 138.84, 131.20, 130.26, 122.23, 70.67, 53.58, 47.80, 38.55, 38.49, 29.10, 14.52, 12.77, 12.27, 0.13. MS (ES+): m/z calc. for $C_{22}H_{26}O_7S$: 434.1; found: 457.2 $[M+Na]^+$.

**Synthesis of NAD(P)H Probes Precursors**

**Compound 17**

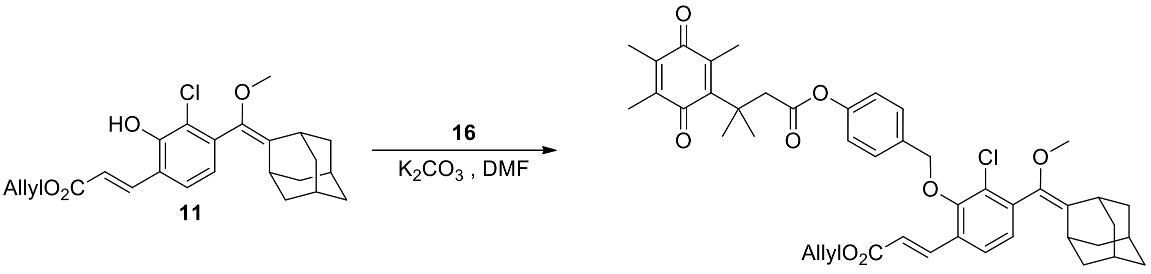


Compound **11** (100 mg, 0.24 mmol) and $K_2CO_3$ (40 mg, 0.29 mmol) were dissolved in DMF (2 mL) and stirred for 10 minutes. Then, mesylate **16** (105 mg, 0.24 mmol) was added and the reaction was stirred at 50°C and monitored by TLC (Hex:EtOAc 85:15). Upon completion, the mixture was diluted with EtOAc (25 mL) and washed with 0.5M HCl (25 mL) and brine (25 ml). The organic layer was dried over $Na_2SO_4$ and evaporated under reduced pressure. The product was purified by column chromatography (Hex:EtOAc 85:15) to afford compound **17** as a yellow oil (141 mg, 78% yield). $^1$H-NMR (400 MHz, $CDCl_3$) δ 7.95 (d, *J* = 16.2 Hz, 1H), 7.48 (d, *J* = 8.4 Hz, 2H), 7.45 (d, *J* = 8.0 Hz, 1H), 7.08 (d, *J* = 8.0 Hz, 1H), 7.00 (d, *J* = 8.4 Hz, 2H), 6.48 (d, *J* = 16.2 Hz, 1H), 5.97 (dq, *J* = 10.8, 5.7 Hz, 1H), 5.35 (dd, *J* = 17.2, 1.3 Hz, 1H), 5.26 (dd, *J* = 10.4, 0.9 Hz, 1H), 4.95 (d, *J* = 4.3 Hz, 2H),

4.70 (d, *J* = 5.6 Hz, 2H), 3.31 (s, 3H), 3.27 (s, 1H), 3.24 (s, 2H), 2.17 (s, 3H), 2.06 (s, 1H), 1.92 (m, 6H), 2.01 – 1.66 (m, 12H), 1.52 (s, 6H). $^{13}$C-NMR (400 MHz, $CDCl_3$) δ 190.42, 186.92, 170.72, 165.73, 153.17, 151.47, 150.08, 142.43, 138.92, 138.66, 138.35, 138.11, 137.80, 133.23, 132.05, 131.75, 129.36, 129.08, 127.42, 124.61, 121.17, 119.66, 117.77, 74.88, 64.80, 56.79, 47.21, 38.73, 38.57, 38.14, 37.95, 36.58, 32.47, 29.24, 28.45, 27.88, 27.73, 13.88, 12.18, 11.65. MS (ES+): m/z calc. for $C_{45}H_{49}ClO_8$: 752.3; found: 775.5 $[M+Na]^+$.

**Compound 18**

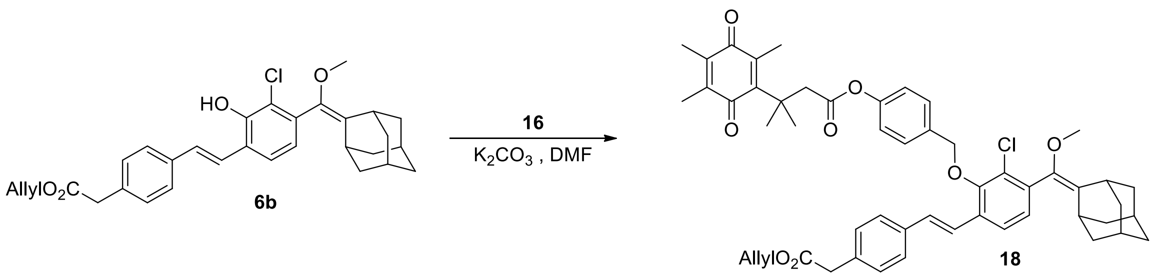


Compound **6b** (150 mg, 0.3 mmol) and $K_2CO_3$ (45 mg, 0.33 mmol) were dissolved in DMF (2 mL) and stirred for 10 minutes. Then, compound **16** (129 mg, 0.3 mmol) was added and the reaction was stirred at 50°C and monitored by TLC (Hex:EtOAc 85:15). Upon completion, the mixture was diluted with EtOAc (25 mL) and washed with 0.5M HCl (25 mL) and brine (25 ml). The organic layer was dried over $Na_2SO_4$ and evaporated under reduced pressure. The product was purified by column chromatography (Hex:EtOAc 85:15) to afford compound **18** as a yellow oil (184 mg, 75% yield). $^{1}$H-NMR (400 MHz, $CDCl_3$) δ 7.53 – 7.48 (m, 3H), 7.41 (d, *J* = 8.2 Hz, 2H), 7.34 (d, *J* = 16.5 Hz, 1H), 7.28 (d, *J* = 8.2 Hz, 2H), 7.12 (d, *J* = 16.5 Hz, 1H), 7.06 (d, *J* = 8.0 Hz, 1H), 7.01 (d, *J* = 8.5 Hz, 2H), 5.92 (ddt, *J* = 17.1, 10.5, 5.7 Hz, 1H), 5.30 (m, 1H), 5.23 (m, 1H), 4.95 (d, *J* = 3.5 Hz, 2H), 4.61 (dt, *J* = 5.7, 1.4 Hz, 2H), 3.67 (s, 2H), 3.33 (s, 3H), 3.29 (s, 1H), 3.26 (s, 2H), 2.18 (s, 3H), 2.11 (s, 1H), 1.93 (m, 6H), 1.97 – 1.70 (m, 12H), 1.54 (s, 6H). $^{13}$C-NMR (101 MHz, $CDCl_3$) δ 190.45, 186.96, 170.80, 170.59, 151.91, 151.51, 149.92, 142.44, 139.29, 138.68, 138.13, 135.60, 134.75, 133.95, 133.29, 131.86, 131.54, 131.31, 130.19, 129.28, 129.02, 127.31, 126.42, 123.38, 121.92, 121.14, 117.90, 76.87, 76.56, 76.24, 74.33, 65.10, 56.62, 47.22, 40.58, 38.61, 38.20, 37.96, 36.67, 32.47, 29.24, 28.47, 27.95, 27.80, 13.90, 12.19, 11.65. MS (ES+): m/z calc. for $C_{52}H_{55}ClO_8$: 842.4; found: 465.6 $[M+Na]^+$.

**Synthesis of NAD(P)H Probes**

**Nt-NCL**

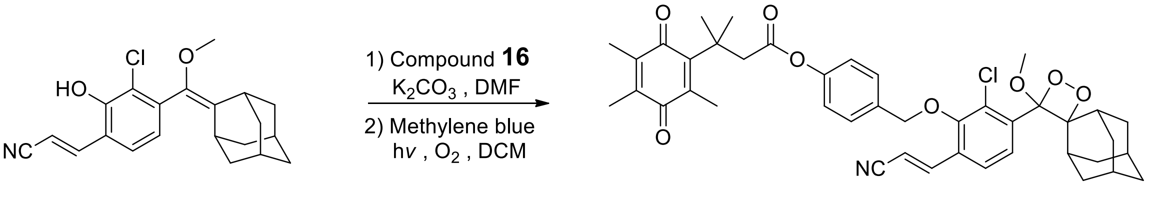


**Nt-NCL**

Acrylonitrile substituted enol ether[14] (100 mg, 0.28 mmol) and $K_2CO_3$ (43 mg, 0.31 mmol) were dissolved in DMF (2 mL) and stirred for 10 minutes. Then, compound **16** (122 mg, 0.28 mmol) was added and the reaction was stirred at 50°C while monitored by TLC (Hex:EtOAc 85:15). Upon completion, the mixture was diluted with EtOAc (50 mL) and washed with 0.5M HCl (50 mL) and brine (50 ml). The organic layer was dried over $Na_2SO_4$ and evaporated under reduced pressure. The residue was dissolved in 20 mL of DCM followed by the addition of a catalytic amount of methylene blue. Then, oxygen was bubbled through the solution while irradiating with yellow light. The reaction was monitored by RP-HPLC. Upon completion (15 min), the solvent was concentrated under reduced pressure and the product was purified by preparative RP-HPLC. **Nt-NCL** was obtained as a yellow solid (170 mg, 83%). $^1$H-NMR (400 MHz, $CDCl_3$) δ 7.94 (d, $J$ = 8.4 Hz, 1H), 7.50 (d, $J$ = 16.9 Hz, 1H), 7.46 (d, $J$ = 8.4 Hz, 1H), 7.40 (d, $J$ = 8.5 Hz, 2H), 7.03 (d, $J$ = 8.5 Hz, 2H), 5.95 (d, $J$ = 16.8 Hz, 1H), 4.90 (s, 2H), 3.26 (s, 2H), 3.21 (s, 3H), 3.02 (s, 1H), 2.29 (d, $J$ = 11.9 Hz, 1H), 2.18 (s, 3H), 1.93 (d, $J$ = 4.0 Hz, 6H), 1.87 – 1.60 (m, 8H), 1.53 (s, 6H), 1.46 (dd, $J$ = 12.9, 2.6 Hz, 1H), 1.38 – 1.30 (m, 1H). $^{13}$C-NMR (400 MHz, $CDCl_3$) δ 190.92, 187.49, 171.23, 153.69, 151.89, 150.93, 144.22, 142.93, 139.34, 138.71, 136.46, 132.93, 130.70, 129.86, 129.29, 128.04, 124.76, 122.01, 117.62, 111.64, 100.00, 96.43, 75.60, 49.81, 47.72, 38.51, 36.58, 33.94, 33.69, 32.73, 32.23, 31.61, 29.02, 26.17, 25.84, 14.44, 12.72, 12.20. MS (ES+): m/z calc. for $C_{42}H_{44}ClNO_8$: 725.3; found: 748.4 $[M+Na]^+$.

**Ac-NCL**

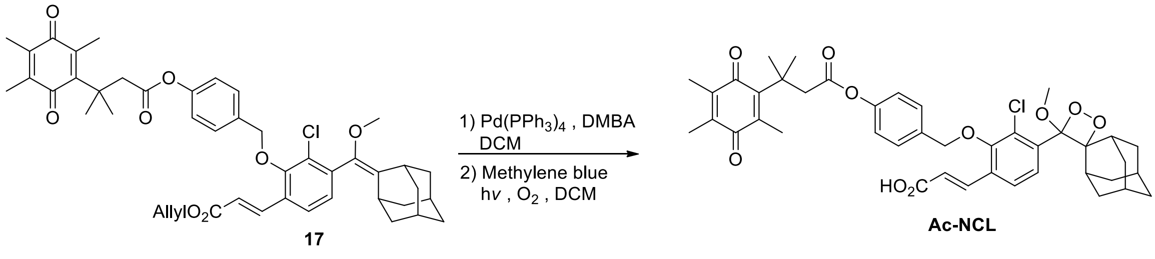


Compound **17** (100 mg, 0.13 mmol) was dissolved in DCM (2 ml), followed by the addition of DMBA (42 mg, 0.27 mmol) and tetrakis(triphenylphosphine)palladium(15 mg, 0.01 mmol). The reaction was stirred at room temperature and monitored by TLC (Hex:EtOAc 70:30). Upon completion, the solvent was concentrated under reduced pressure and the crude product was filtered through short silica gel pad using EtOAc as eluent. The filtrate was concentrated under reduced pressure and the crude residue dissolved in DCM (10 mL), followed by the addition of a catalytic amount methylene blue. Then, oxygen was bubbled through the solution while irradiating with yellow light. The reaction was monitored by RP-HPLC. Upon completion (15 min), the solvent was concentrated under reduced pressure and the product was purified by preparative RP-HPLC. **Ac-NCL** was obtained as a yellow solid (50 mg, 52%). $^{1}$H-NMR (400 MHz, $CDCl_3$) δ 8.00 – 7.91 (m, 2H), 7.61 (d, *J* = 8.3 Hz, 1H), 7.46 (d, *J* = 8.1 Hz, 2H), 7.01 (d, *J* = 8.4 Hz, 2H), 6.49 (d, *J* = 16.1 Hz, 1H), 4.91 (s, 2H), 3.25 (s, 2H), 3.22 (s, 3H), 3.03 (s, 1H), 2.31 (d, *J* = 12.6 Hz, 1H), 2.17 (s, 3H), 2.00 (s, 1H), 1.95 – 1.91 (m, 6H), 1.91 – 1.45 (m, 10H), 1.52 (s, 6H). $^{13}$C-NMR (400 MHz, $CDCl_3$) δ 190.41, 186.98, 170.77, 170.28, 153.84, 151.41, 150.26, 142.42, 139.91, 138.78, 138.18, 135.31, 132.81, 130.69, 129.50, 128.65, 127.35, 125.01, 121.31, 120.03, 111.23, 95.92, 76.87, 76.55, 76.23, 75.24, 49.28, 47.20, 37.98, 36.10, 33.42, 33.15, 32.18, 31.74, 31.11, 28.48, 25.69, 25.35, 13.91, 12.18, 11.66. MS (ES+): m/z calc. for $C_{42}H_{45}ClO_{10}$: 744.3; found: 767.5 $[M+Na]^{+}$.

**St-NCL**

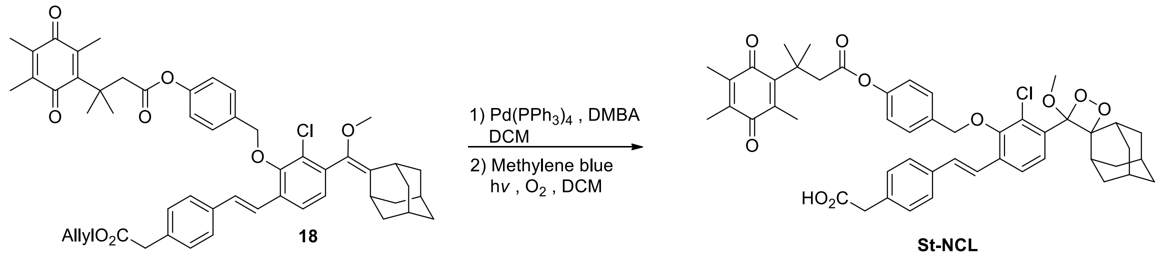


Compound **18** (100 mg, 0.12 mmol) was dissolved in DCM (2 ml), followed by the addition of DMBA (37 mg, 0.24 mmol) and tetrakis(triphenylphosphine)palladium(14 mg, 0.01 mmol). The reaction was stirred at room temperature and monitored by TLC (Hex:EtOAc 70:30). Upon completion, the solvent was concentrated under reduced pressure and the crude was filtered through a short silica gel pad using EtOAc as eluent. The filtrate was concentrated under reduced pressure and the crude residue dissolved in DCM (10 mL), followed by the addition of a catalytic amount methylene blue. Then, oxygen was bubbled through the solution while irradiating with yellow light. The reaction was monitored by RP-HPLC. Upon completion (15 min), the solvent was concentrated under reduced pressure and the product was purified by preparative RP-HPLC. **St-NCL** was obtained as a yellow solid (44 mg, 44%). $^{1}$H-NMR (400 MHz, DMSO) δ 7.89 (d, *J* = 8.5 Hz, 1H), 7.76 (d, *J* = 8.4 Hz, 1H), 7.51 (d, *J* = 8.5 Hz, 2H), 7.46 (d, *J* = 8.2 Hz, 2H), 7.37 (d, *J* = 16.5 Hz, 1H), 7.25 (m, 4H), 7.03 (d, *J* = 8.5 Hz, 2H), 4.93 (d, *J* = 4.0 Hz, 2H), 3.56 (s, 2H), 3.17 (s, 2H), 3.11 (s, 3H), 2.87 (s, 1H), 2.24 (d, *J* = 12.1 Hz, 1H), 2.10 (s, 3H), 1.94 (s, 1H), 1.84 (m, 6H), 1.76 – 1.52 (m, 8H), 1.46 (s, 6H), 1.33 (d, *J* = 10.9 Hz, 1H). $^{13}$C-NMR (101 MHz, DMSO) δ 190.85, 187.25, 172.96, 171.23, 153.03, 152.20, 150.61, 142.94, 139.23, 138.52, 135.82, 135.51, 134.62, 134.50, 132.75, 131.78, 130.41, 130.25, 128.83, 127.25, 127.08, 125.05, 122.20, 121.45, 111.86, 95.92, 75.08, 49.85, 47.34, 38.61, 36.44, 33.83, 33.59, 32.33, 32.25, 31.63, 31.39, 29.54, 28.94, 26.06, 25.74, 14.52, 12.92, 12.32. MS (ES+): m/z calc. for $C_{49}H_{51}ClO_{10}$: 834.3; found: 857.5 $[M+Na]^{+}$.

## NMR and MS spectra of key compounds

**2**

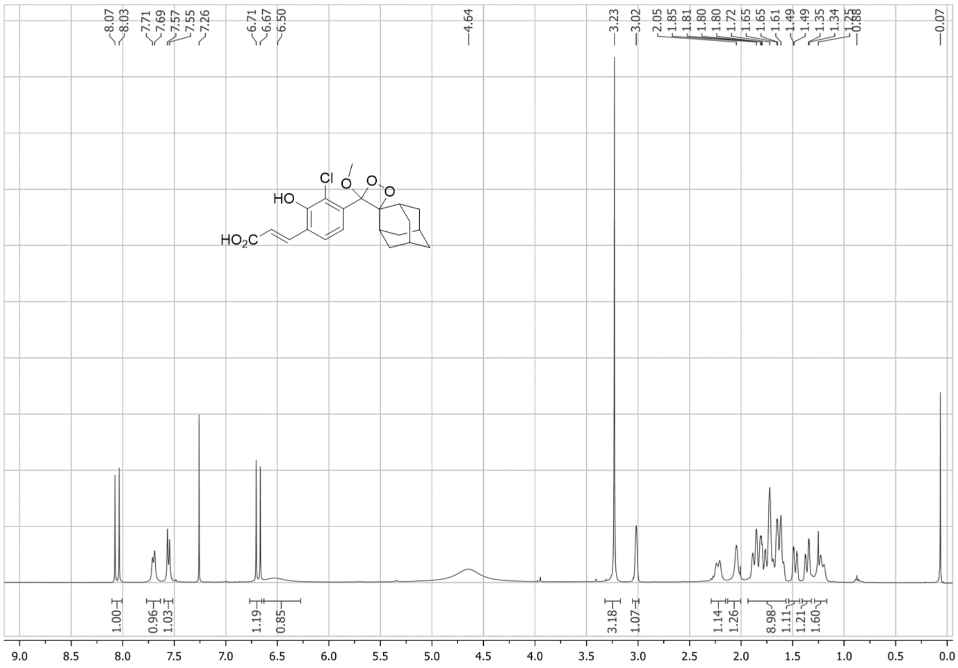

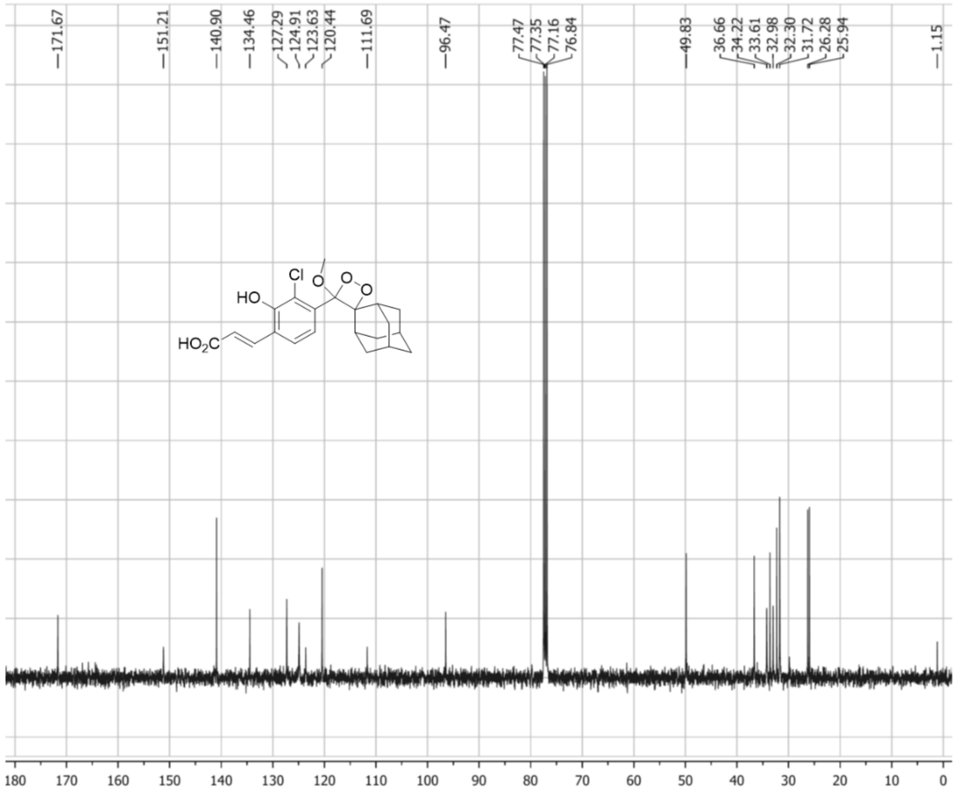

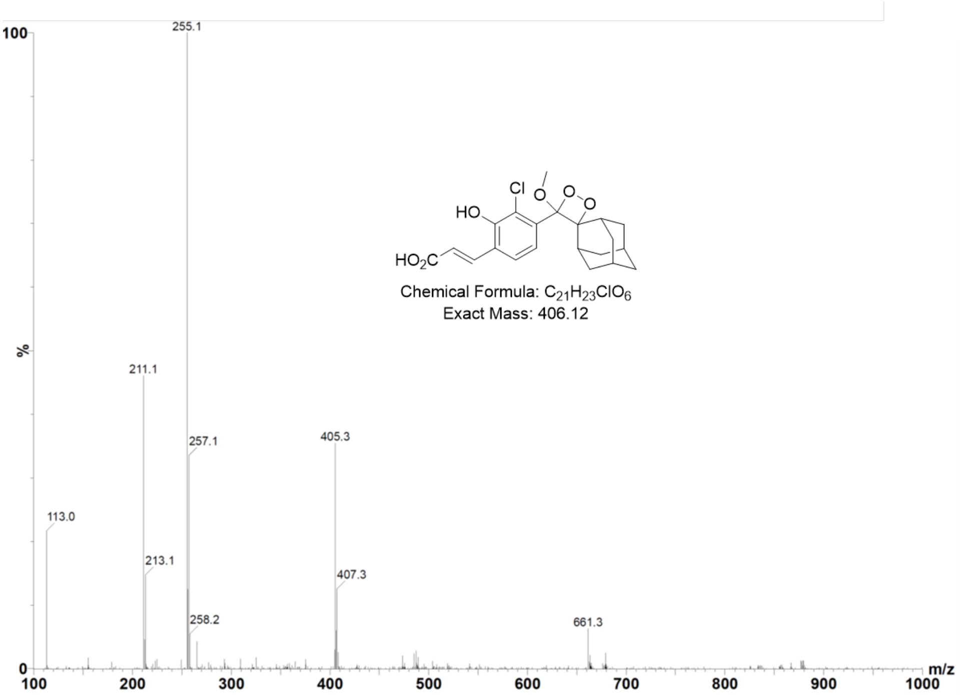
Chemical Formula: $C_{21}H_{23}ClO_6$
Exact Mass: 406.12
HO
Cl
$HO_2C$
255.1
211.1
257.1
405.3
113.0
213.1
407.3
258.2
661.3
%
m/z

**3a**

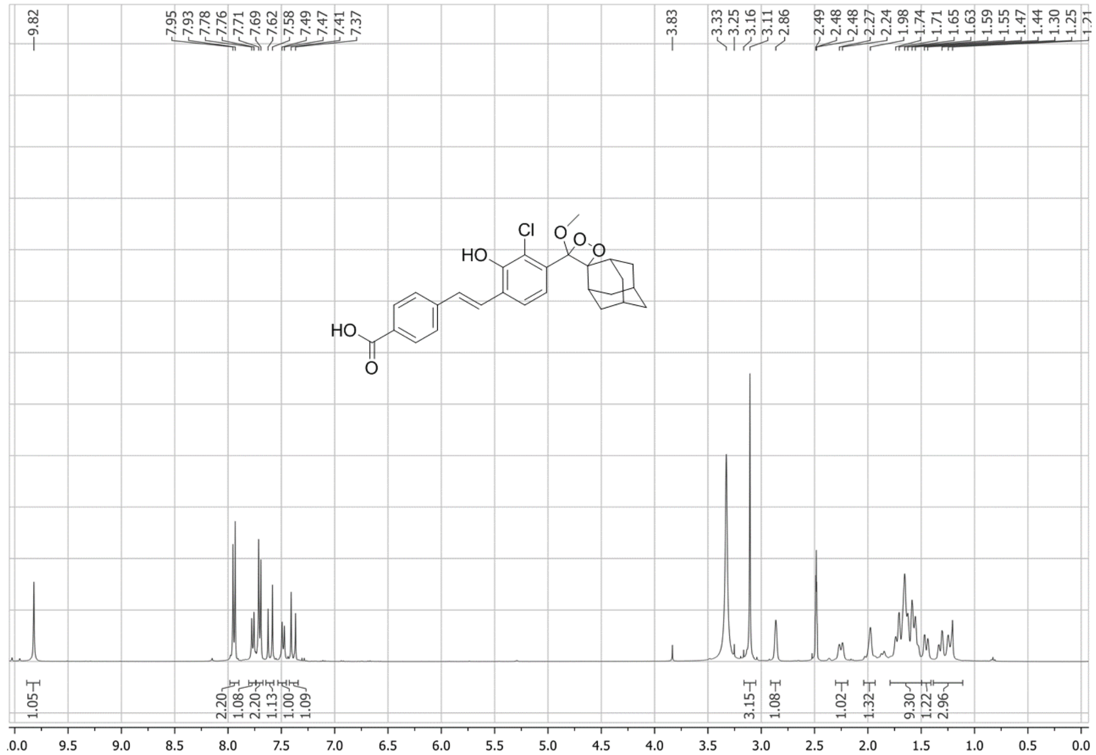
HO
Cl
HO
O

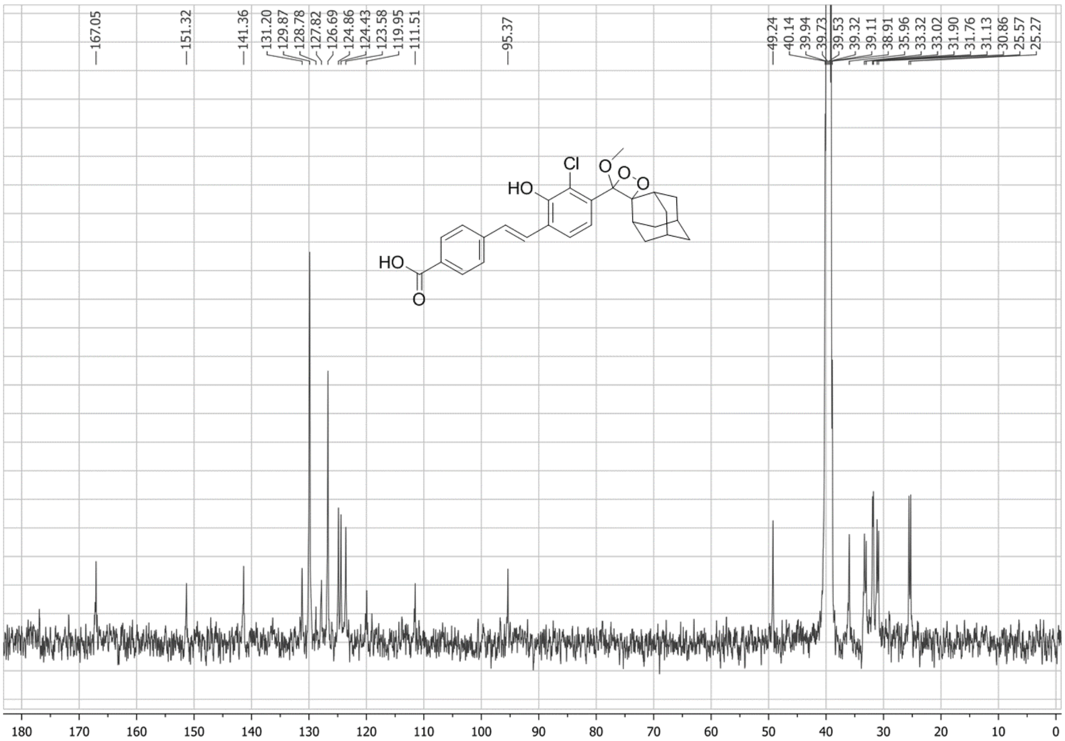

167.05
151.32
141.36
131.20
129.87
128.78
127.82
126.69
124.86
124.43
123.58
119.95
111.51
95.37
49.24
40.14
39.94
39.73
39.53
39.32
39.11
38.91
35.96
33.32
33.02
31.90
31.76
31.13
30.86
25.57
25.27
Cl
HO
HO
O


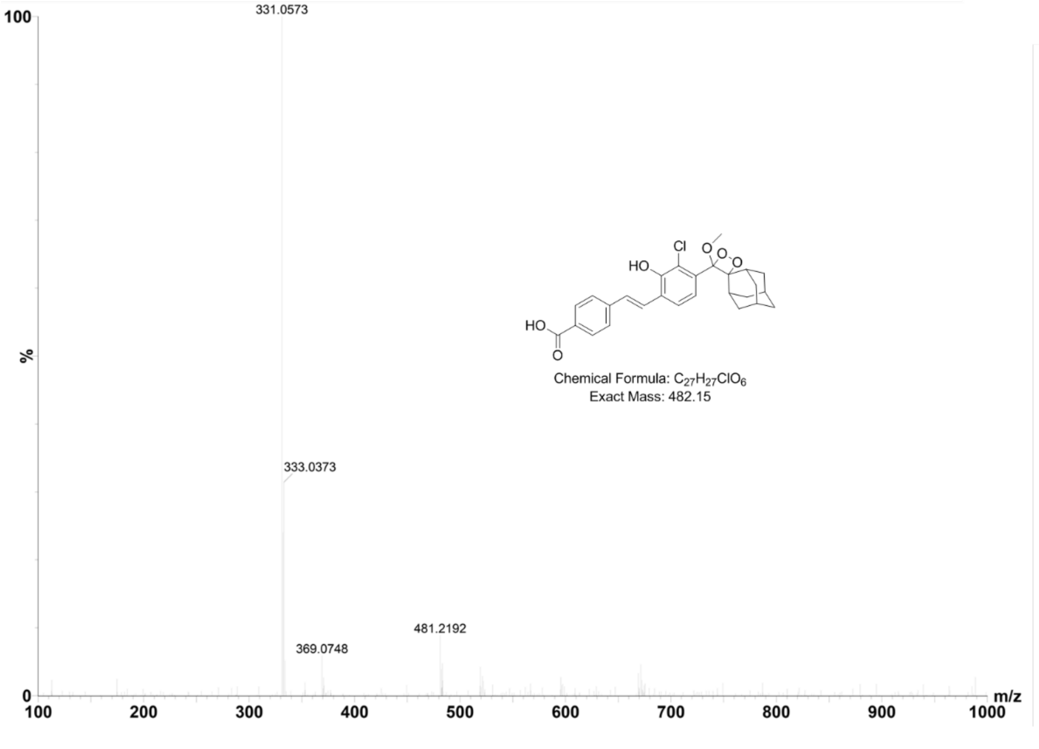

100
%
0
331.0573
333.0373
369.0748
481.2192
m/z
100
200
300
400
500
600
700
800
900
1000
Cl
HO
HO
O
Chemical Formula: $C_{27}H_{27}ClO_6$
Exact Mass: 482.15

**3b**

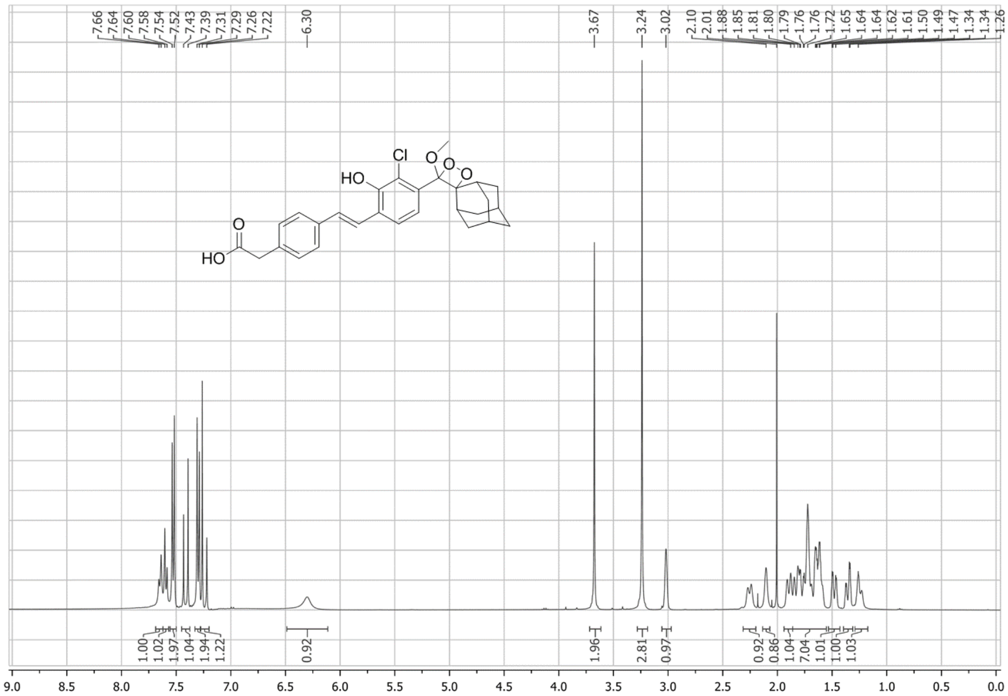
7.66
7.64
7.60
7.58
7.54
7.52
7.43
7.39
7.31
7.29
7.26
7.22
6.30
3.67
3.24
3.02
2.10
2.01
1.88
1.85
1.81
1.80
1.79
1.76
1.76
1.72
1.65
1.64
1.64
1.62
1.61
1.50
1.49
1.47
1.34
1.34
1.26
Cl
HO
O
O
O
O
HO
1.00
1.02
1.97
1.04
1.94
1.22
0.92
1.96
2.81
0.97
0.92
0.86
1.04
7.04
1.01
1.00
1.03
9.0
8.5
8.0
7.5
7.0
6.5
6.0
5.5
5.0
4.5
4.0
3.5
3.0
2.5
2.0
1.5
1.0
0.5
0.0

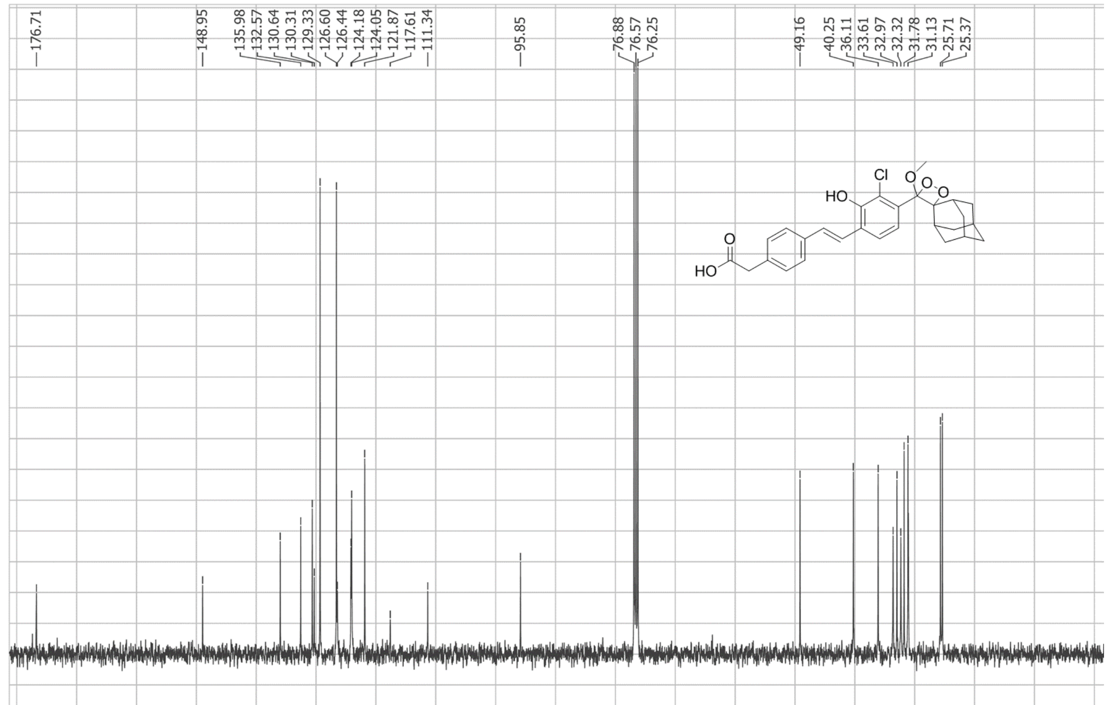
176.71
148.95
135.98
132.57
130.64
130.31
129.33
126.60
126.44
124.18
124.05
121.87
117.61
111.34
95.85
76.88
76.57
76.25
49.16
40.25
36.11
33.61
32.97
32.32
31.78
31.13
25.71
25.37
Cl
HO
O
O
O
O
HO
180
170
160
150
140
130
120
110
100
90
80
70
60
50
40
30
20
10
0

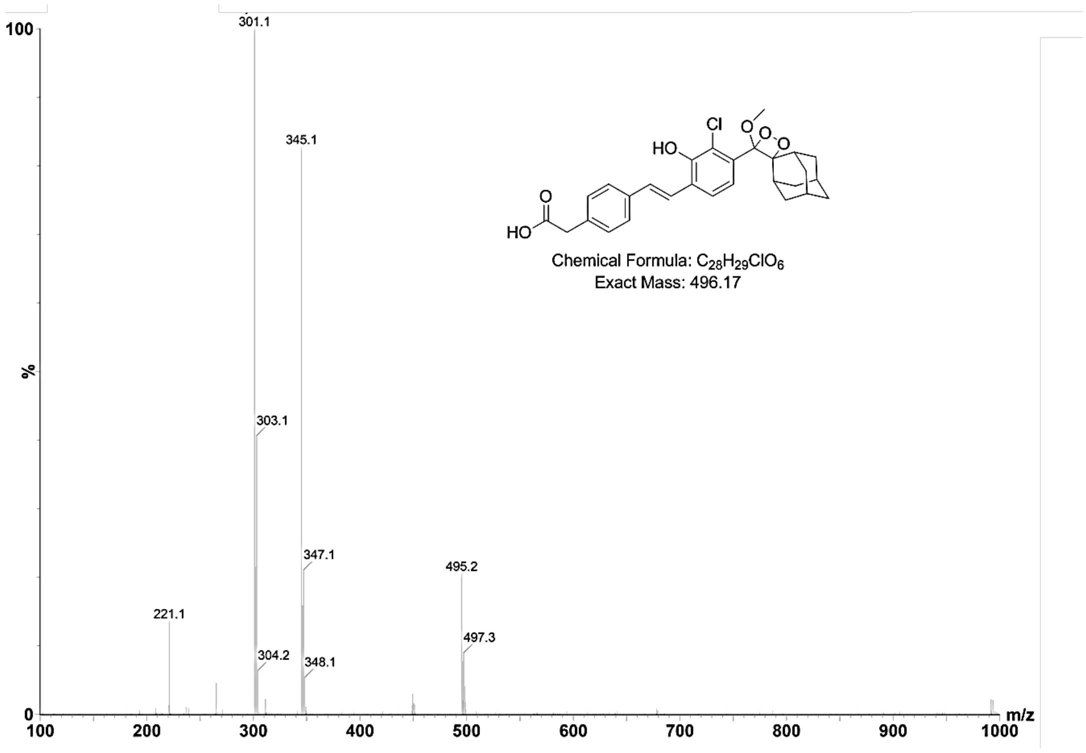
100
301.1
345.1
%
303.1
347.1
495.2
221.1
497.3
304.2
348.1
0
m/z
100
200
300
400
500
600
700
800
900
1000
Cl
HO
O
O
O
HO
Chemical Formula: $C_{28}H_{29}ClO_6$
Exact Mass: 496.17

**3c**

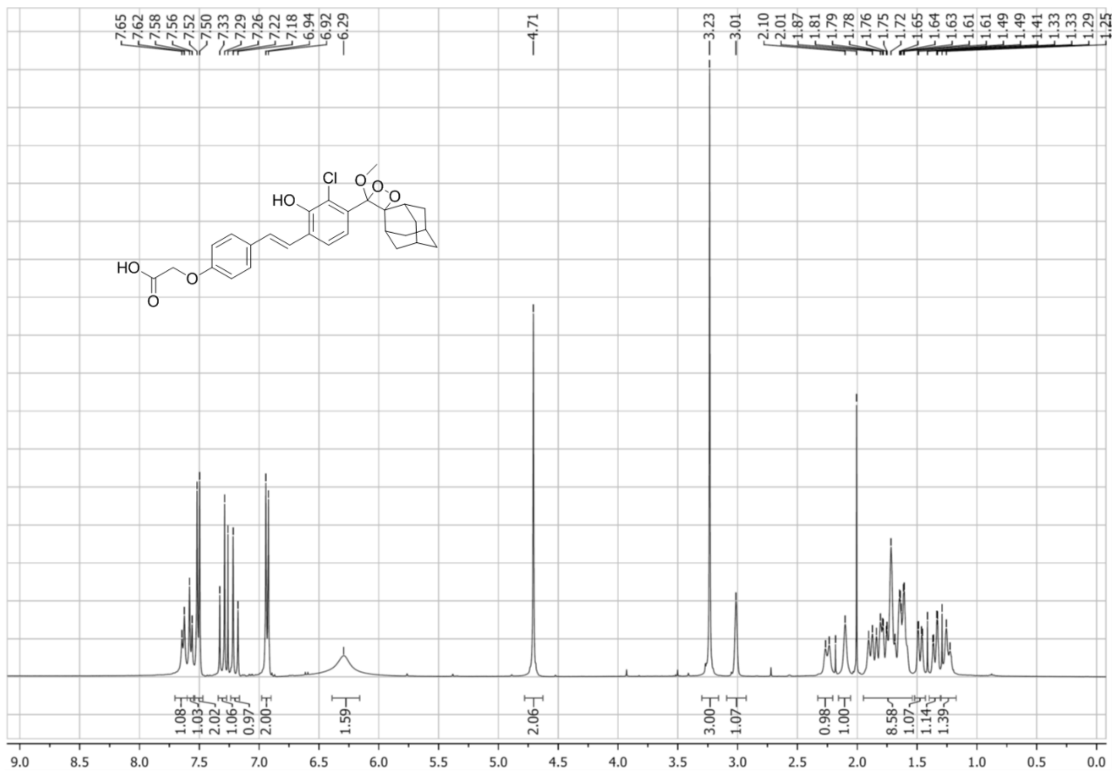
7.65
7.62
7.58
7.56
7.52
7.50
7.33
7.29
7.26
7.22
7.18
6.94
6.92
6.29
4.71
3.23
3.01
2.10
2.01
1.87
1.81
1.79
1.78
1.76
1.75
1.72
1.65
1.64
1.63
1.61
1.61
1.49
1.49
1.41
1.33
1.33
1.29
1.25
Cl
HO
O
O
HO
O
1.08
1.03
2.02
1.06
0.97
2.00
1.59
2.06
3.00
1.07
0.98
1.00
8.58
1.07
1.14
1.39
9.0
8.5
8.0
7.5
7.0
6.5
6.0
5.5
5.0
4.5
4.0
3.5
3.0
2.5
2.0
1.5
1.0
0.5
0.0

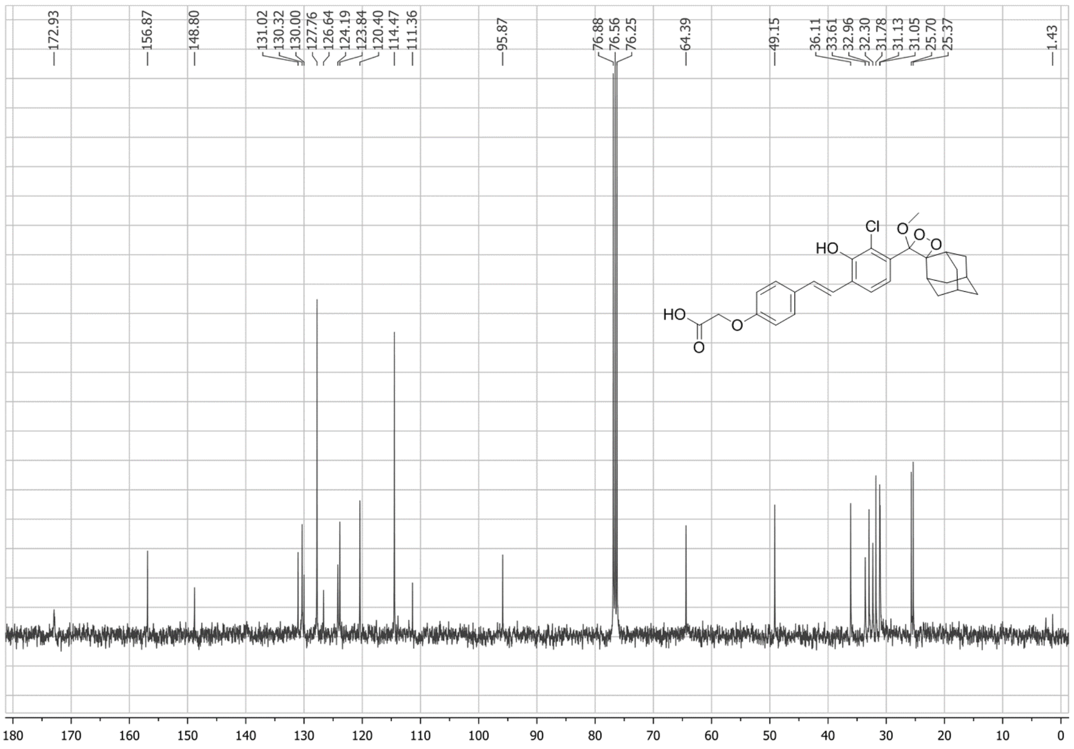
172.93
156.87
148.80
131.02
130.32
130.00
127.76
126.64
124.19
123.84
120.40
114.47
111.36
95.87
76.88
76.56
76.25
64.39
49.15
36.11
33.61
32.96
32.30
31.78
31.13
31.05
25.70
25.37
1.43
HO
Cl
O
HO
180 170 160 150 140 130 120 110 100 90 80 70 60 50 40 30 20 10 0

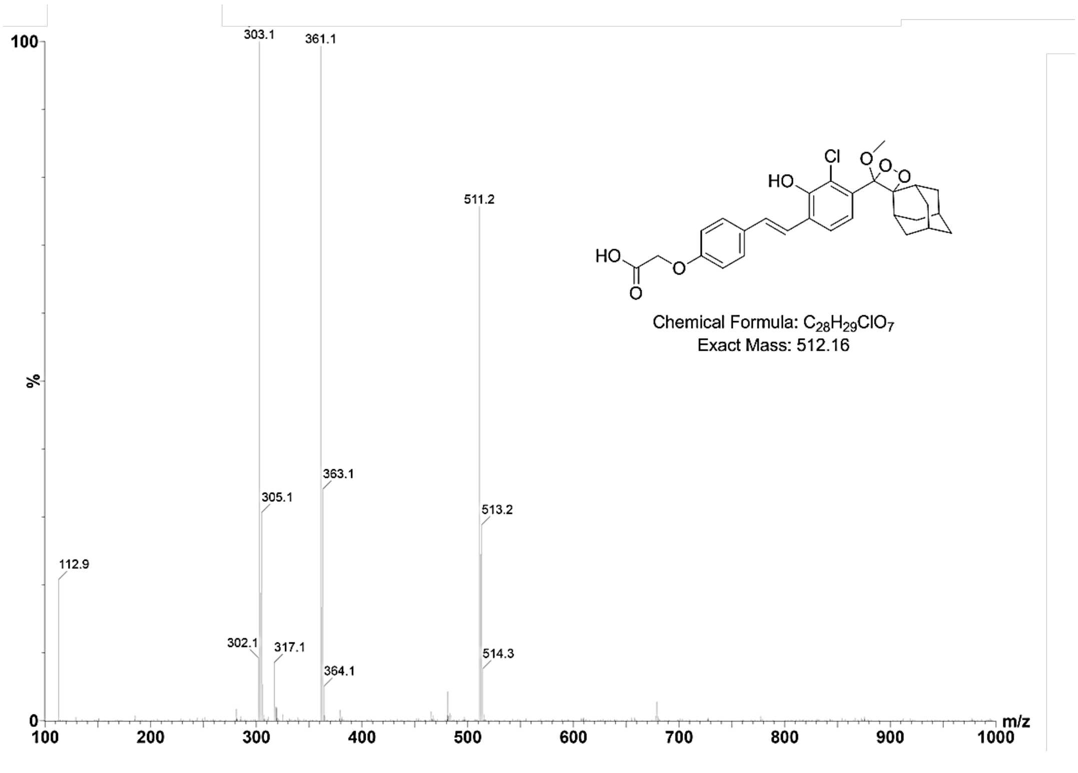
100
%
0
303.1
361.1
511.2
363.1
305.1
513.2
112.9
302.1
317.1
364.1
514.3
m/z
100 200 300 400 500 600 700 800 900 1000
HO
Cl
O
HO
Chemical Formula: $C_{28}H_{29}ClO_7$
Exact Mass: 512.16

**Nt-NCL**

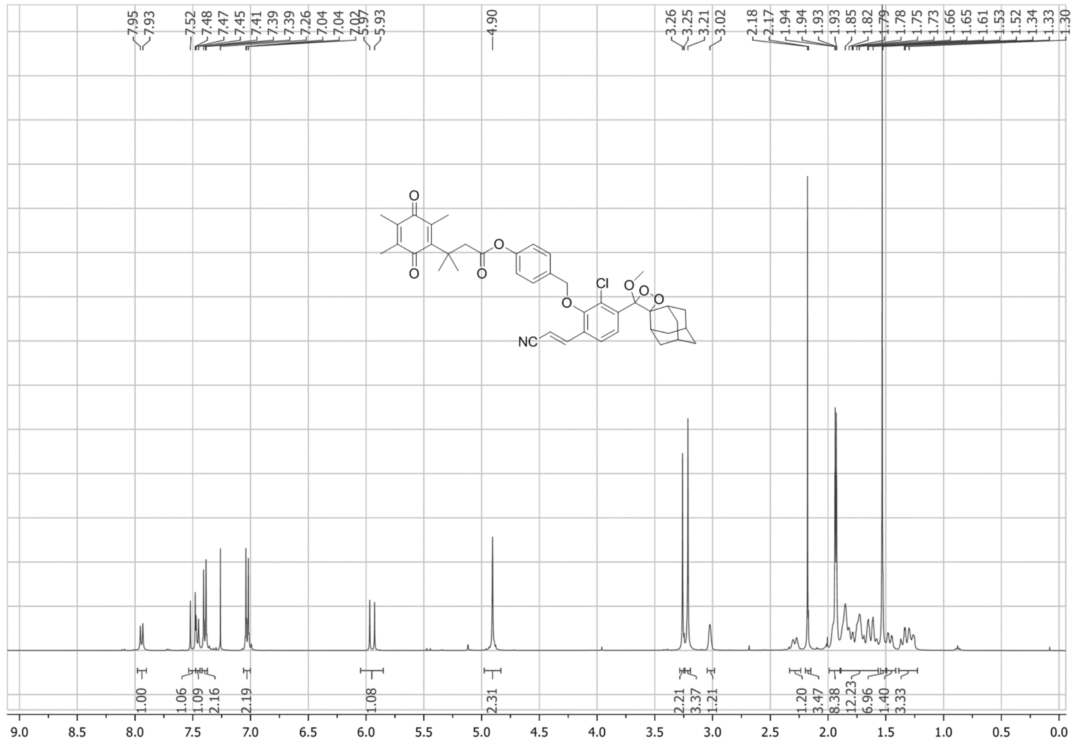

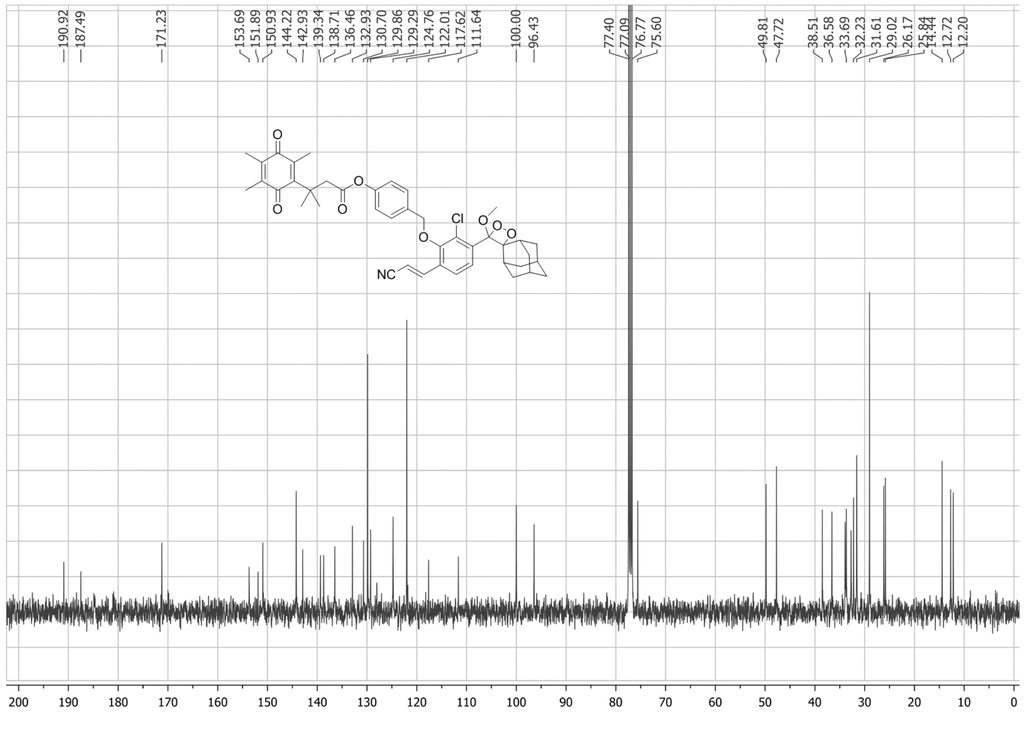

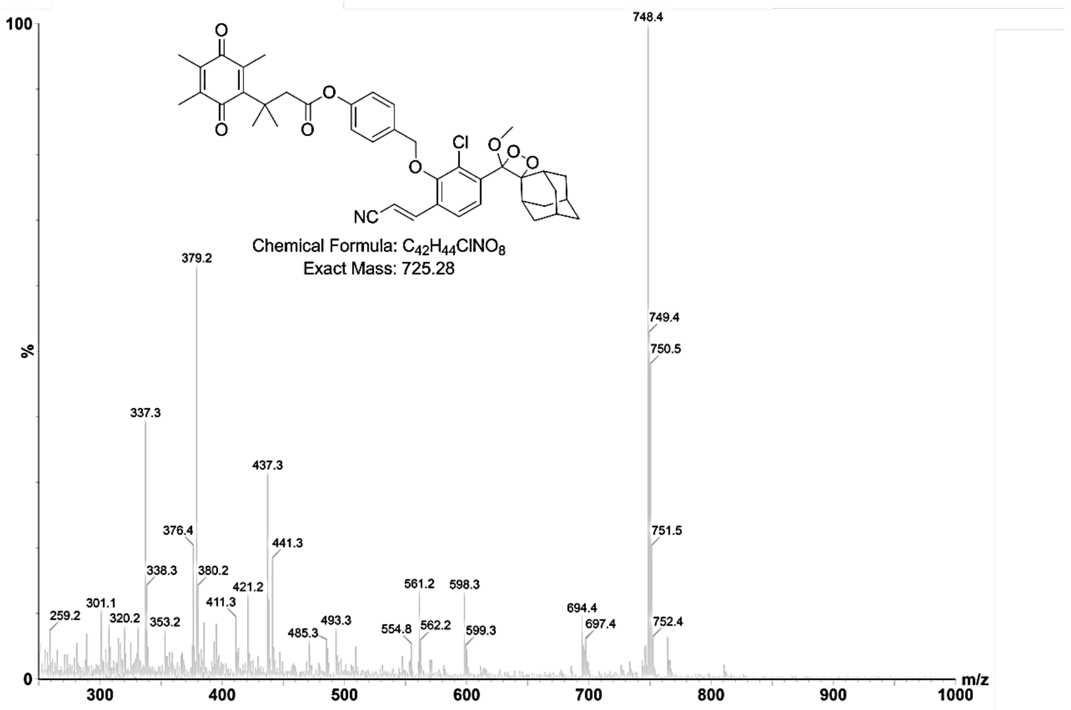
Chemical Formula: $C_{42}H_{44}ClNO_8$
Exact Mass: 725.28
NC
Cl
748.4
379.2
749.4
750.5
337.3
437.3
376.4
441.3
751.5
338.3
380.2
421.2
411.3
561.2
598.3
301.1
259.2
320.2
353.2
485.3
493.3
554.8
562.2
599.3
694.4
697.4
752.4
%
m/z
100
0
300
400
500
600
700
800
900
1000

**Ac-NCL**

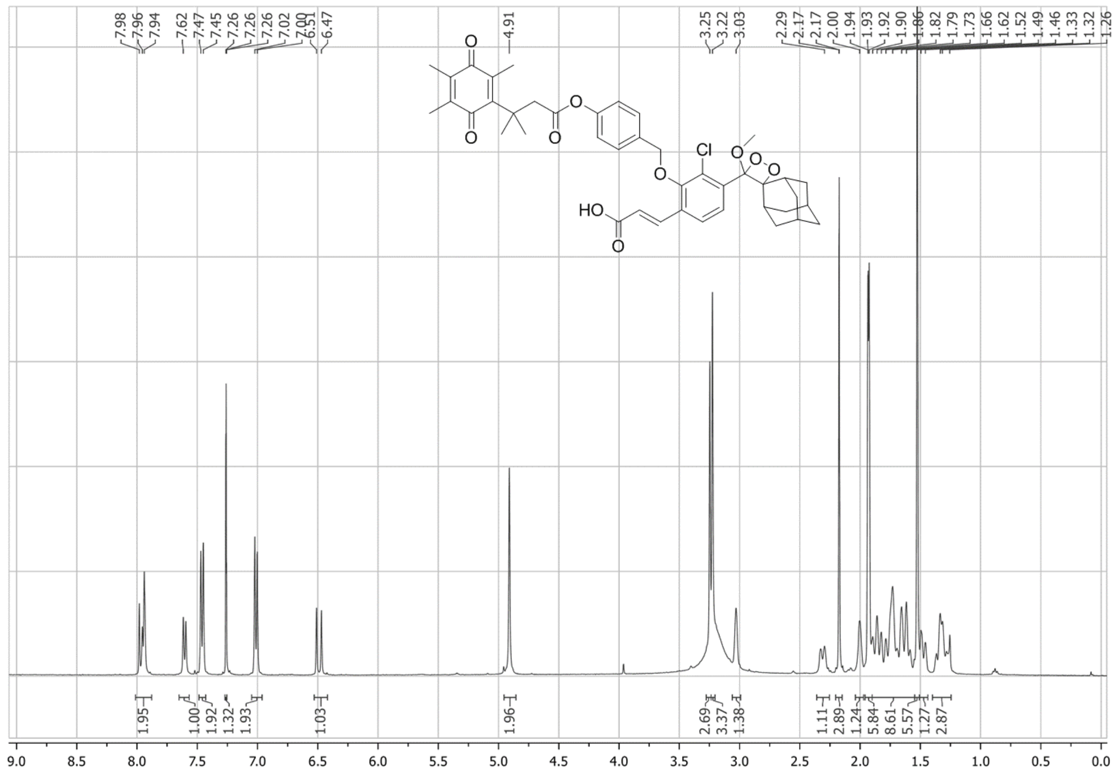
HO
Cl
7.98
7.96
7.94
7.62
7.47
7.45
7.26
7.26
7.26
7.02
7.00
6.51
6.47
4.91
3.25
3.22
3.03
2.29
2.17
2.17
2.00
1.94
1.93
1.92
1.90
1.86
1.82
1.79
1.73
1.66
1.62
1.52
1.49
1.46
1.33
1.32
1.26
1.95
1.00
1.92
1.32
1.93
1.03
1.96
2.69
3.37
1.38
1.11
2.89
1.24
5.84
8.61
5.57
1.27
2.87
9.0
8.5
8.0
7.5
7.0
6.5
6.0
5.5
5.0
4.5
4.0
3.5
3.0
2.5
2.0
1.5
1.0
0.5
0.0

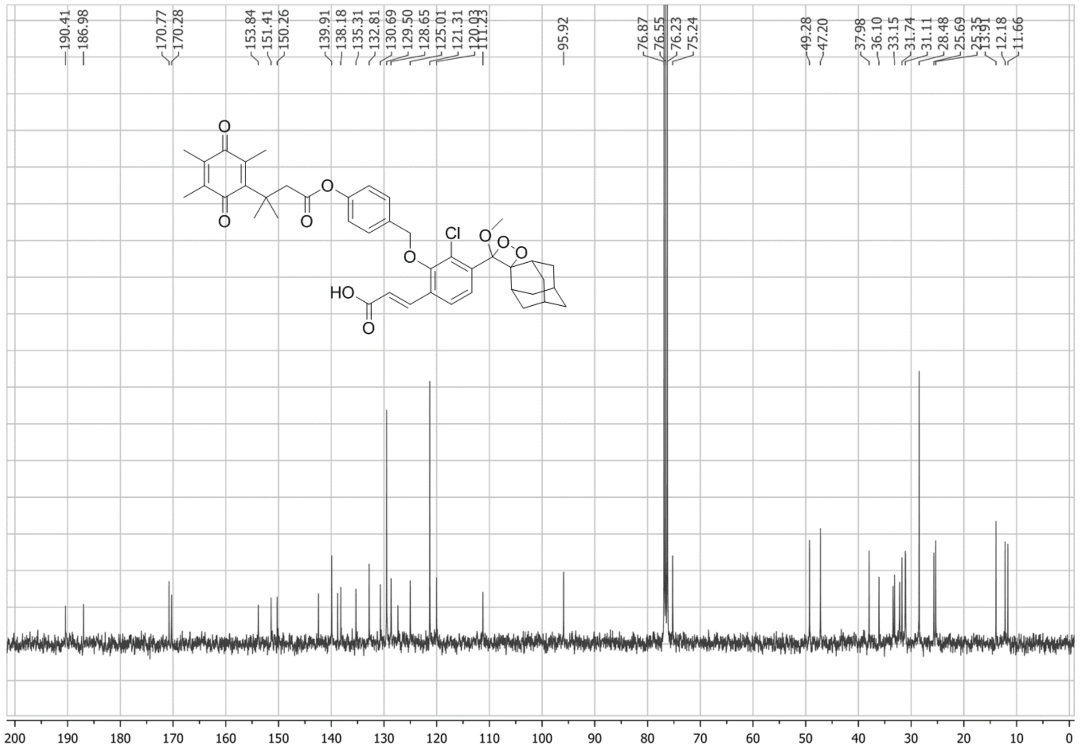
190.41
186.98
170.77
170.28
153.84
151.41
150.26
139.91
138.18
135.31
132.81
130.69
129.50
128.65
125.01
121.31
120.03
111.23
95.92
76.87
76.55
76.23
75.24
49.28
47.20
37.98
36.10
33.15
31.74
31.11
28.48
25.69
25.35
13.91
12.18
11.66
O
O
O
O
Cl
O
O
O
O
HO
O

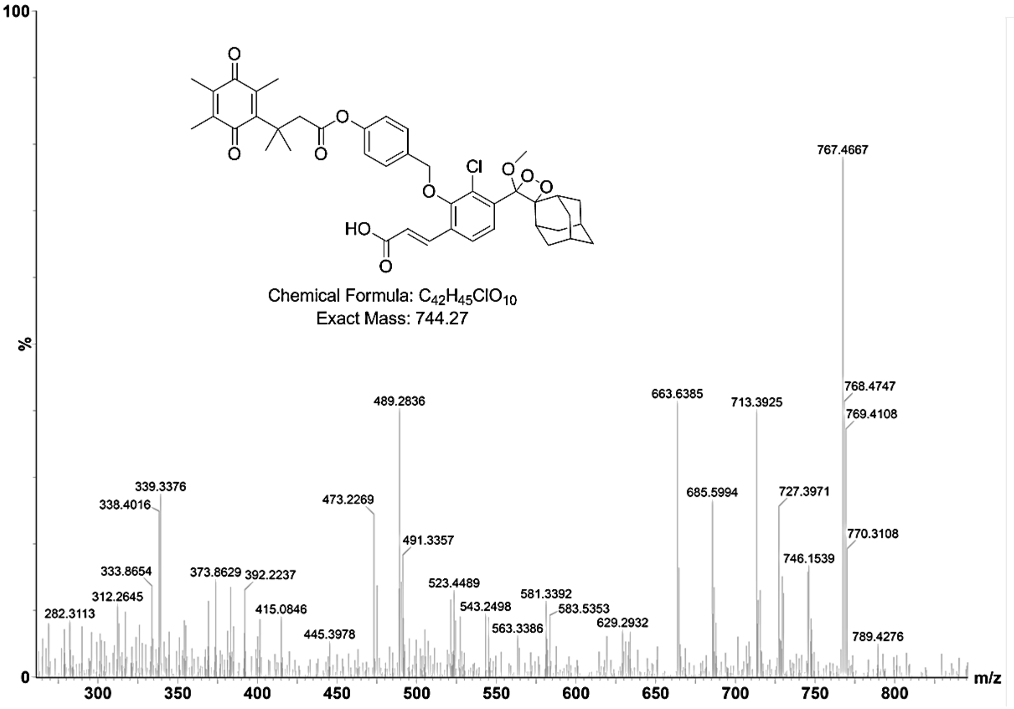
100
%
0
m/z
Chemical Formula: C42H45ClO10
Exact Mass: 744.27
767.4667
768.4747
769.4108
770.3108
663.6385
713.3925
489.2836
685.5994
727.3971
339.3376
338.4016
473.2269
491.3357
746.1539
333.8654
373.8629
392.2237
523.4489
312.2645
581.3392
282.3113
415.0846
543.2498
583.5353
629.2932
445.3978
563.3386
789.4276

**St-NCL**

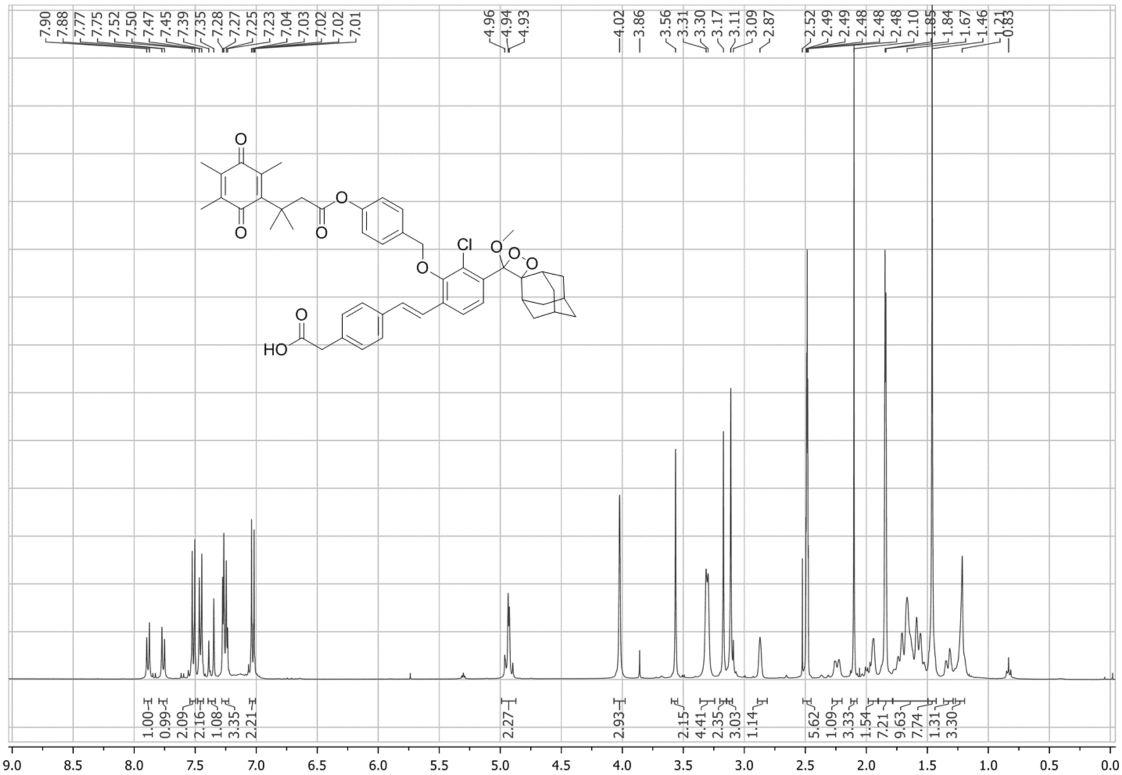

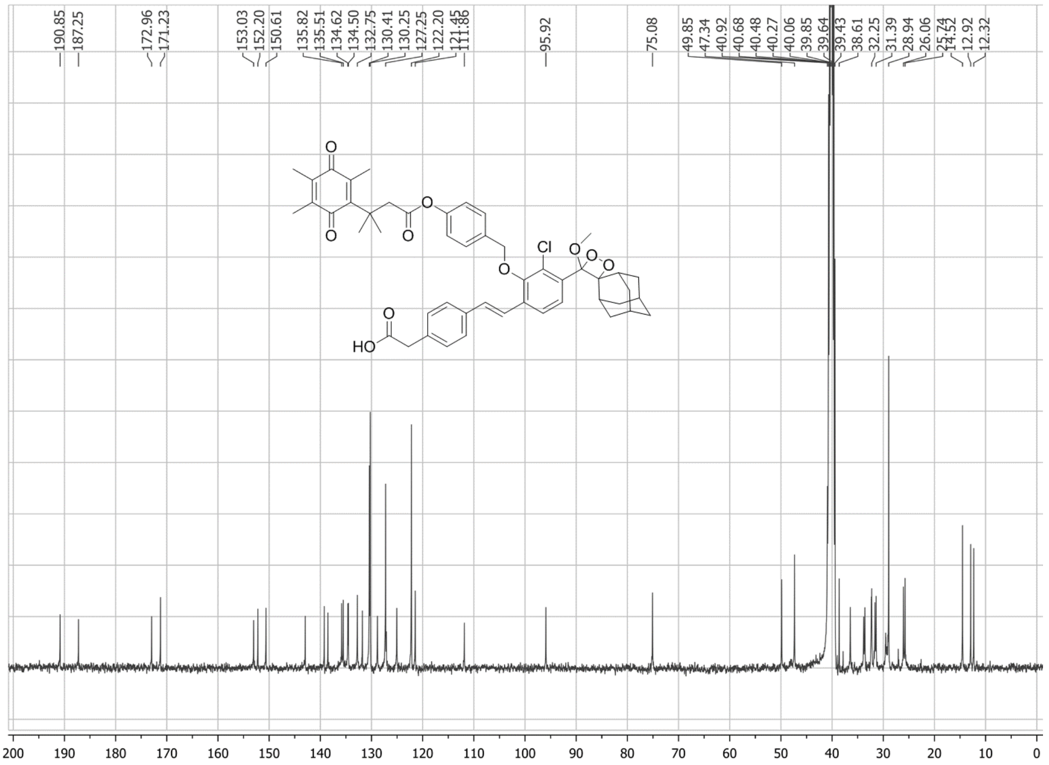

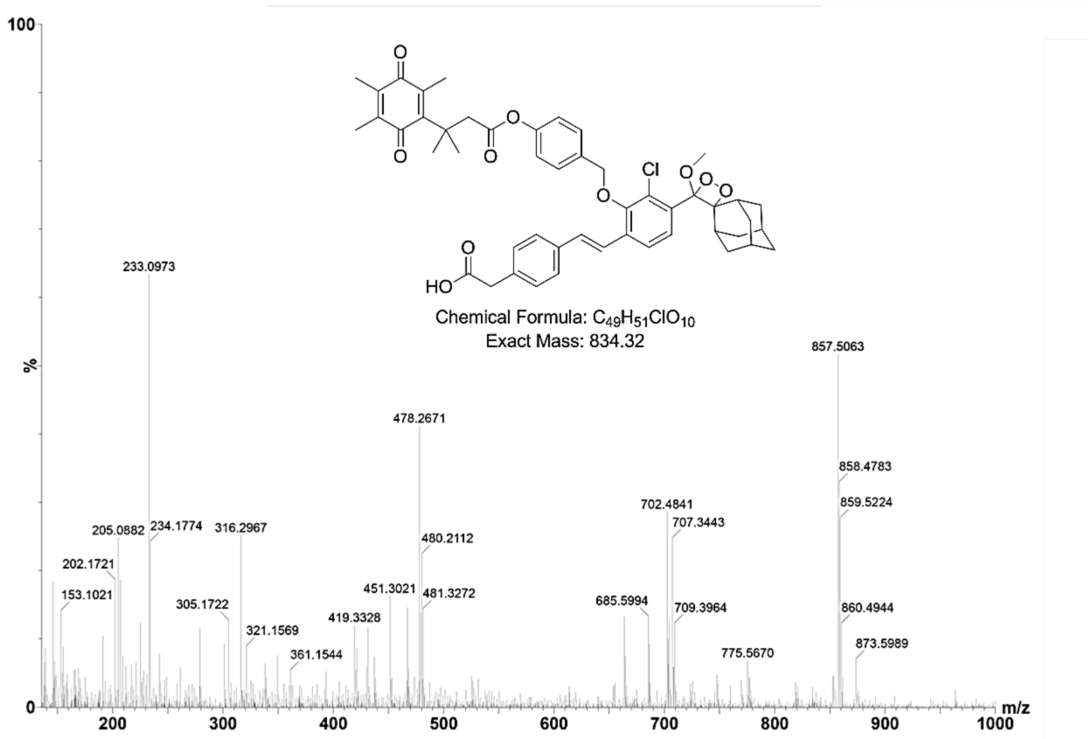

100
%
0
m/z
200
300
400
500
600
700
800
900
1000
233.0973
857.5063
478.2671
858.4783
702.4841
859.5224
707.3443
205.0882
234.1774
316.2967
480.2112
202.1721
153.1021
451.3021
481.3272
305.1722
419.3328
685.5994
709.3964
860.4944
321.1569
361.1544
775.5670
873.5989
Chemical Formula: $C_{49}H_{51}ClO_{10}$
Exact Mass: 834.32

## References


(1) Silvers, W. C.; Prasai, B.; Burk, D. H.; Brown, M. L.; Mccarley, R. L. **2012**.

(2) Zhao, Y.; Truhlar, D. G. *Theor. Chem. Acc.* **2008**, *120* (1–3), 215–241.

(3) Weigend, F.; Ahlrichs, R. *Phys. Chem. Chem. Phys.* **2005**, *7* (18), 3297–3305.

(4) Frisch, M. J. et al. Gaussian 09, Revision D.01, Gaussian, Inc.: Wallingford, CT 2013.

(5) Glendening, E. D.; Badenhoop, J. K.; Reed, A. E.; Carpenter, J. E.; Bohmann, J. A.; Morales, C. M.; Landis, C. R.; Weinhold, F. NBO 6.0. (Theoretical Chemistry Institute, University of Wisconsin, Madison, WI, 2013), http://nbo6.chem.wisc.edu/.

(6) Hansch, C.; Leo, A.; Taft, R. W. *Chem. Rev* **1991**, *91*, 165–195.

(7) Blanksby, S. J.; Ellison, G. B. *Acc. Chem. Res.* **2003**, *36* (4), 255–263.

(8) Marenich, A. V; Cramer, C. J.; Truhlar, D. G. *J. Phys. Chem. B* **2009**, *113* (18), 6378–6396.

(9) MacroModel, version 11.7, Schrödinger, LLC, New York, NY, 2017.

(10) Harder, E.; Damm, W.; Maple, J.; Wu, C.; Reboul, M.; Xiang, J. Y.; Wang, L.; Lupyan, D.; Dahlgren, M. K.; Knight, J. L.; et al. *J. Chem. Theory Comput.* **2016**, *12* (1), 281–296.

(11) Santiago, C. B.; Milo, A.; Sigman, M. S. *J. Am. Chem. Soc.* **2016**, *138* (40), 13424–13430.

(12) Legault, C. Y. CYLView, 1.0b; Université de Sherbrooke: Sherbrooke, Quebec, Canada, 2009; http://www.cylview.org.

(13) Pátek, M.; Smrčina, M.; Nakanishi, E.; Izawa, H. *J. Comb. Chem.* **2000**, *2* (4), 370–377.

(14) Green, O.; Eilon, T.; Hananya, N.; Gutkin, S.; Bauer, C. R.; Shabat, D. *ACS Cent. Sci.* **2017**, *3* (4), 349–358.

(15) Zimmerman, S. S.; Khatri, A.; Garnier-Amblard, E. C.; Mullasseril, P.; Kurtkaya, N. L.; Gyoneva, S.; Hansen, K. B.; Traynelis, S. F.; Liotta, D. C. *J. Med. Chem.* **2014**, *57* (6), 2334–2356.

(16) J., B. K.; Ori, G.; A., S. T.; Doron, S.; J., C. C. *Angew. Chem. Int. Ed.* **2018**, *57* (25), 7508–7512.

(17) Hananya, N.; Green, O.; Blau, R.; Satchi-Fainaro, R.; Shabat, D. *Angew. Chem. Int. Ed.* **2017**, *56* (39), 11793–11796.

(18) Rohde, R. D.; Agnew, H. D.; Yeo, W. S.; Bailey, R. C.; Heath, J. R. *J. Am. Chem. Soc.* **2006**, *128* (29), 9518–9525.

(19) M., S. J.; Francesca, V.; A., A. S.; Rudi, F. *Angew. Chem. Int. Ed.* **2011**, *50* (22), 5075–5080.
